# The Chemical, Physical and Biological Hazards in Bee Products: A Comprehensive Review

**DOI:** 10.3390/molecules31213831

**Published:** 2026-10-23

**Authors:** Małgorzata Makarewicz, Iwona Drożdż, Aleksandra Duda

**Affiliations:** 1 Department of Fermentation Technology and Microbiology, Faculty of Food Technology, University of Agriculture in Krakow, ul. Balicka 122, 30-149 Krakow, Poland; malgorzata.makarewicz@urk.edu.pl (M.M.); iwona.drozdz@urk.edu.pl (I.D.); 2 Centre for Innovation and Research on Prohealthy and Safe Food, University of Agriculture in Krakow, ul. Balicka 104, 30-149 Krakow, Poland

**Keywords:** pesticides, heavy metals, allergens, antibiotic residues, phytotoxins, mycotoxins, microplastic, 5-hydroxymethylfurfural (5-HMF), tutin, triptolide, polycyclic aromatic hydrocarbons (PAHs), health risk, hazard quotient (HQ)

## Abstract

This review consolidates data on the most significant chemical, biological, and physical contaminants found in honey and other bee products that may have a negative impact on human health. It focuses on the various groups of compounds that affect the safety and quality of these bee products, such as pesticides, heavy metals, antibiotic residues, allergens, phytotoxins, 5-hydroxymethylfurfural, polycyclic aromatic hydrocarbons, mycotoxins, and microplastics. The levels of these contaminants and the related hazard risk they pose are presented in one place. The sources of these contaminants, as well as the influence of various factors (e.g., geographic region, apiary location, nectar-producing plant species and measurement method) on their concentrations, are also discussed. The levels of contaminants reported in the literature were compared with current standards and regulations to determine the actual risk to consumer health by calculating the hazard quotient. A risk assessment matrix summarising the risk arising from different groups of contaminants in specific types of apicultural products and showing the possibility of cumulative exposure is provided. Gaps in the current state of knowledge and directions for future research were also identified.

## 1. Introduction

Natural products, particularly those with proven health-promoting properties, have long attracted significant consumer interest. Bee-derived products occupy a prominent position among them due to their high nutritional value and their role as valuable dietary supplements. They are also a rich source of bioactive compounds, including vitamins, enzymes, polyphenols, macro- and microelements, organic acids, terpenoids and alkaloids [1,2,3,4]. Furthermore, they exhibit a wide range of biological activities, including antimicrobial, anti-inflammatory, antitumour and immunostimulatory effects, as well as protective properties against various harmful environmental and physiological factors [5,6]. Due to these characteristics, bee products have been widely used for centuries in traditional medicine, particularly in apitherapy [7].

**Figure 1:**
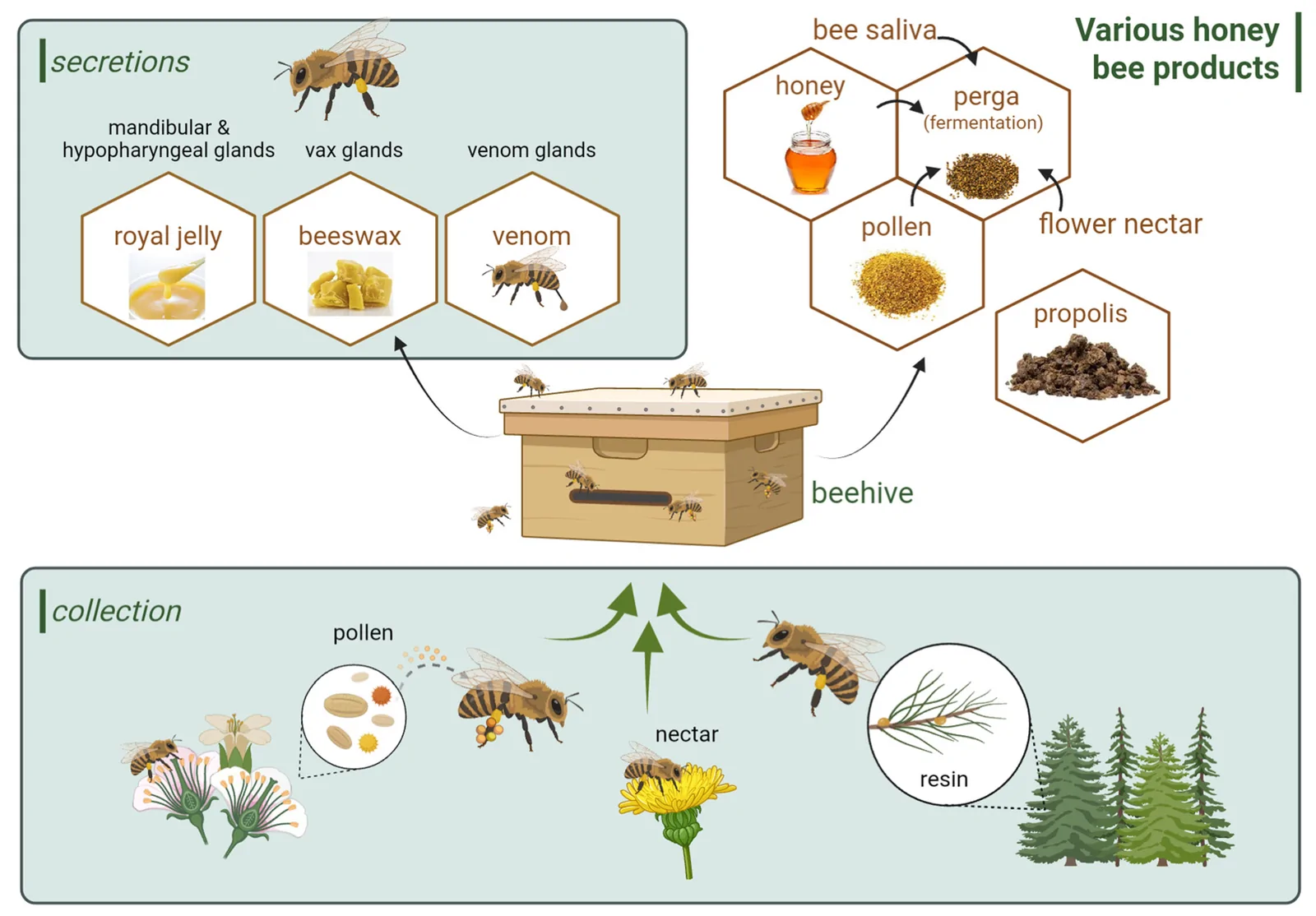
Various bee products and their sources and formation. Created in BioRender. Duda, A. (2026) https://BioRender.com/hebs023 (accessed on 19 October 2026).

The most commonly used bee products with these beneficial properties are honey, bee pollen, bee bread (perga), propolis and royal jelly (Figure 1) [7]. Pollen is a product harvested by bees and transferred to the hive in the form of pollen loads known as “bee pollen”. Pollen is packed into the honeycomb cells and enriched by the bees with honey, as well as with digestive enzymes and organic acids from the secretions of the salivary glands. In such a form, under anaerobic conditions, pollen undergoes a lactic acid fermentation in order to preserve it. This natural product, obtained from the fermentation of bee pollen mixed with bee saliva and flower nectar inside the honeycomb cells of a hive, is called bee bread, perga, or ambrosia [8,9]. Propolis is a resinous substance collected and transformed by honey bees from various plant sources, which they use as a multifunctional material in the construction and maintenance of their hives. Propolis is believed to have many antibiotic, antifungal, antiviral and antitumour properties; however, it can also act as an allergen [10,11]. Royal jelly is produced by the mandibular and hypopharyngeal glands of young worker bees. This creamy, viscous, white, and highly acidic substance has the unique ability to transform an ordinary female worker larva into a queen bee with an extended lifespan and egg-laying capabilities. Royal jelly is composed of water (50–70%), proteins (9–18%), carbohydrates (7–18%), and lipids and fatty acids (3–8%), as well as water-soluble vitamins, polyphenols, hormones, nucleotides, and sterols [12].

Numerous scientific reports provide evidence to support the use of bee products in the prevention and treatment of various diseases. However, despite their well-documented health benefits, consuming bee-derived products may pose significant health risks, primarily due to the potential presence of chemical (e.g., allergens, phytotoxins, pesticides, antibiotics, and heavy metals) and microbiological (e.g., microorganisms and mycotoxins) contaminants [13,14].

Consumers expect food products to be safe (i.e., free from biological, chemical, or physical agents that could endanger human health, or containing such agents only at recognised safe levels). Therefore, ensuring the quality and sanitary safety of bee products is a crucial part of their production and distribution.

In the era of globalisation, consumers have access to a wide variety of bee products from different geographical regions and producers around the world. However, due to inconsistent or absent legal regulations across countries, the quality and safety standards of such products can vary considerably. Consequently, conscientious and responsible producers are facing growing challenges in guaranteeing the health and safety of bee products at every stage of the production process. It is also crucial to educate consumers about the potential health risks of consuming low-quality honey and other bee products, as well as products that are unregulated or from unreliable sources.

The quality of bee products can be significantly affected by various contaminants, which can be categorised as either environmental (indirect) or beekeeping-related (direct). Indirect contamination occurs when bees collect nectar, water or pollen and transfer toxic substances to the hive, while direct contamination may result from the improper production, transport or storage of final products. It is worth emphasising that, to date, numerous papers have focused on either a single bee product and one [15,16,17] or more groups [18,19,20,21] of the contaminants present in it, or on a single group of contaminants in one [22,23,24] or more types of bee product [25,26,27]. This review takes a completely new approach to the topic, bringing together various beekeeping products and different contaminants in one place. This enables readers to assess multiple contaminants occurring simultaneously in specific bee products. The review therefore compiles data on the most serious chemical, biological, and physical contaminants detected in honey, bee pollen, perga, propolis and royal jelly that negatively affect human health. It also discusses the sources of these contaminants and the influence of various factors, such as geographic region, apiary location, nectar-producing plant species, and measurement method. The levels of contaminants reported in the literature were compared with current standards, and the actual risk to consumer health was determined by calculating the hazard quotient (HQ). Finally, gaps in current knowledge and future research directions are suggested.

In the search for the publications and other relevant documents required to prepare this review, all available databases, platforms and search engines were utilised. These included Web of Science, Scopus, Google Scholar, ScienceDirect, PubMed, ResearchGate, Academia, JSTOR, DOAJ, EBSCO, AGRICOLA, Embase, Archive.org, Science.gov and Refseek, amongst others. Searches were also carried out on the websites of organisations such as the WHO, FAO and EFSA, as well as on the websites of individual journals and publishers (e.g., Elsevier, Springer Link, Oxford Academic Journals, MDPI, ACS Journals, AIP Journals, APS Journals, RSC Journals, SAGE Journals, De Gruyter, Taylor & Francis and Wiley Online Library). Various combinations of the following keywords were used in the search: pesticide(s) (along with the listing of specific chemical substances used in agriculture and fruit growing), insecticide(s), acaricide(s), fungicide(s), herbicide(s), polychlorinated biphenyls, antibiotic(s) (oxytetracycline, enrofloxacin, tetracycline, β-lactam antibiotics, ampicillin, sulphonamides, aminoglycosides, chloramphenicol, etc.), allergy, hypersensitivity, allergen(s), phytotoxin(s), toxin(s), mycotoxin(s), ochratoxin A, zearalenone, trichothecene(s), aflatoxin(s), fumonisin(s), *Clostridium botulinum*, botulin, heavy metal(s), cadmium (Cd), copper (Cu), lead (Pb), zinc (Zn), nickel (Ni), manganese (Mn), mercury (Hg), chromium (Cr), cobalt (Co), iron (Fe), arsenic (As), 5-hydroxymethylfurfural (5-HMF), polycyclic aromatic hydrocarbons (PAHs), pyrrolizidine alkaloids (PA), tropane alkaloids (TA), grayanotoxin(s), matrine alkaloids, *Gelsemium*, gelsedine-type alkaloids, nicotine, tutin, triptolide, microplastic(s), nanoplastic(s), absorption, level, concentration, contamination, hazard, risk, health, honey, mad honey, bee products, propolis, bee bread, royal jelly, beeswax, bee pollen, bee venom, pollen, flower nectar, bee, beehive, apiary, pollinators, limit of quantification (LOQ), level of concern (LOC), maximum residue limits (MRLs), reference dose (RfD), average daily intake, acceptable daily intake daily (ADI), estimated daily intake (EDI), hazard index (HI), hazard quotient (HQ), cancer risk, harmful impact, microbiome, microbiota, symptoms, IgE, skin allergy tests, prick-to-prick test, anaphylaxis, cross-reaction(s), major royal jelly protein (MRJP), expansins, oxidative stress, nephro-, hepato-, neuro- and immunotoxicity.

The main criteria for selecting literature sources were: access to the full text of the publication; data available in English, Spanish or Polish; and a publication date between 2010 and 2026. In exceptional cases, older publications were used, for example, when discussing the earliest research on a given topic. The findings of original studies were used to prepare this paper; however, review articles were frequently cited in the theoretical introduction in order to reduce the number of references. Database searches were carried out between September 2025 and August 2026.

## 2. Chemical Contamination of Bee Products

### 2.1. Pesticides

Although consumer awareness of the negative health impacts of various pesticides is growing, these substances are primarily identified and analysed in raw materials and plant-based products, such as fruit and vegetables [28,29,30,31,32]. The contamination of honey and other bee products with various pesticides, including herbicides and insecticides is a serious and widely recognised problem for producers, but consumer awareness in this area still needs to be improved [33]. Pesticides are chemicals or biological substances used to prevent, control, or eliminate large populations of weeds, insects, and fungi, among others [14,34]. In 2019, the World Health Organization (WHO) classified pesticides into five classes based on their acute oral toxicity (LD_50_) [35]. Thus, extremely hazardous pesticides belong to Class Ia, highly hazardous ones belong to Class Ib. These are followed by Classes II and III, which are moderately and slightly hazardous, respectively (Table 1). The final class, Class U, refers to substances that are unlikely to cause acute hazard to humans or mammals. However, Class U pesticides can still be highly toxic to honeybees (e.g., specific synthetic pyrethroids or insect growth regulators).

**Figure 2:**
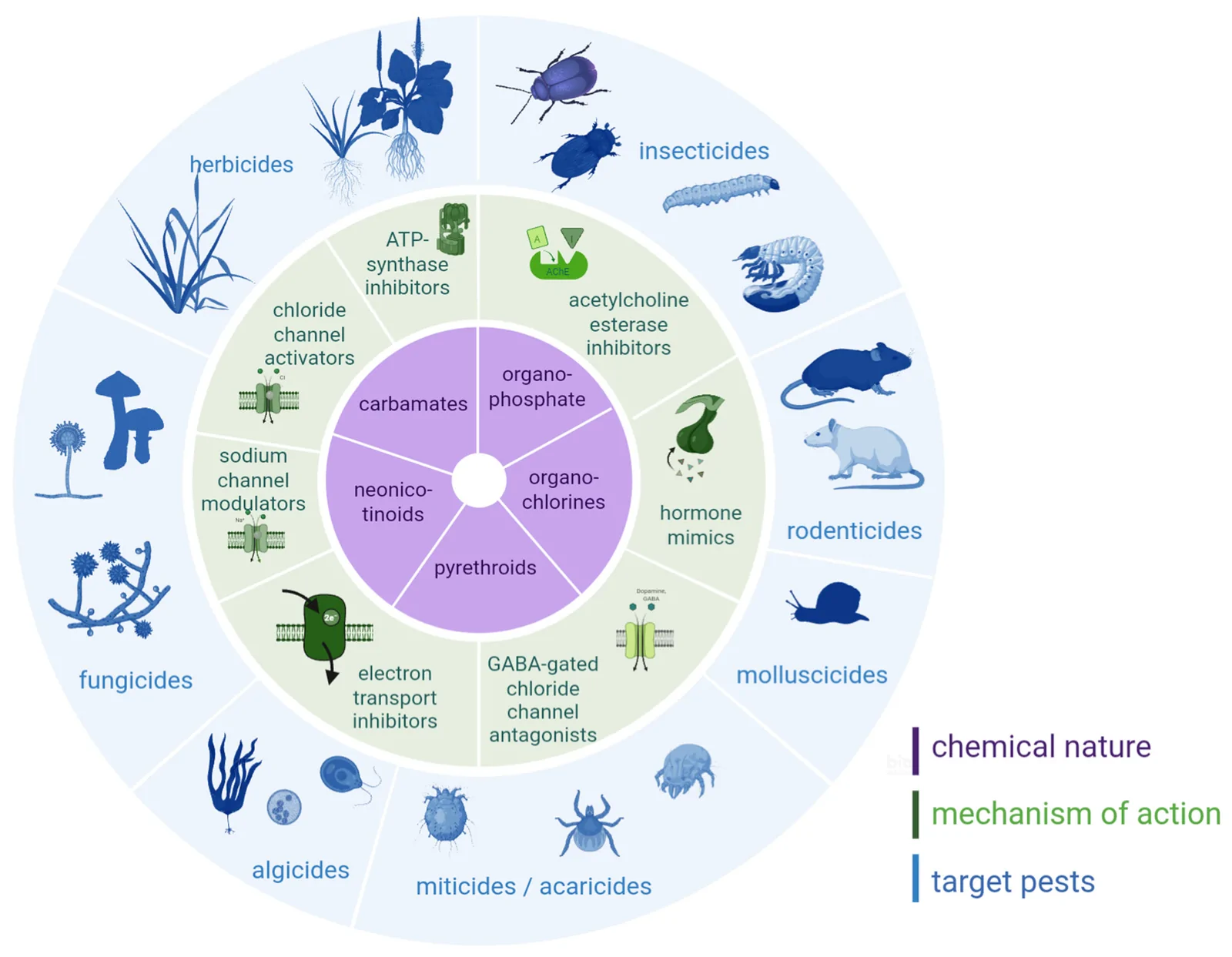
Classification of pesticides that may affect bees and the quality of bee products according to their chemical nature, mechanism of action, and target pests. Created in BioRender. Duda, A. (2026) https://BioRender.com/hebs023.

Pesticides vary in their chemical structure and mechanism of action (Figure 2). Some must first enter target organisms, such as weeds, insects or fungi, and bind to a specific receptor site before becoming toxic and destroying the target organism [36]. Others display immediate toxic effects upon contact with their target organism. Pesticides can be classified according to several criteria. According to the IRAC (Insecticide Resistance Action Committee)/MoA (Mode of Action) classification, pesticides are categorised based on their mechanism of action [37,38]. Examples include acetylcholine esterase inhibitors (e.g., carbamates and organophosphorus compounds), nicotinic acetylcholine receptor agonists (e.g., neonicotinoids), sodium channel modulators (e.g., pyrethroids, pyrethrins, and dichlorodiphenyltrichloroethane (DDT)), GABA-gated channel modulators (e.g., phenylpyrazoles and cyclodiene organochlorines), chloride channel activators (e.g., avermectins), inhibitors of mitochondrial ATP synthase (e.g., azocyclotin), and inhibitors of mitochondrial complexes I–IV of the electron transport chain, to name a few [39]. This classification primarily applies to insecticides. Another criterion is the type of pest being controlled, i.e., the group of organisms that the product affects. Examples include rodenticides, fungicides, insecticides, herbicides, acaricides, miticides, algicides, and general-purpose pesticides. Pesticides may also be classified as chemical, physical or biological (biopesticides). Further categorisation can be based on origin (natural, synthetic or chemical), chemical structure (e.g., organochlorines (OC), organophosphates (OP), carbamates (C), pyrethroids (PY), phenylpyrazoles (PP), triazine derivatives (T), and neonicotinoids (NN)), or the target site of action (e.g., nervous and muscular cells, the respiratory system, the intestinal tract, or growth and developmental centres) [36,39,40,41].

**Table 1:** Hazard classes of pesticides according to WHO recommendations, along with examples of compounds that may be toxic to honey bees or accumulate in bee products [35,42,43].

Class	Hazard Level Description	Acute Oral LD50 for Rats (mg/kg bw)	Examples of Pesticides Found in Bees or Bee Products	Primary Use and Context in Apiculture /Chemical Structure
Ia	extremely hazardous	<5	Parathion * Chlorethoxyphos *	Insecticide, acaricide/OP Insecticide/OP
Ib	highly hazardous	5–50	Dichlorvos * Coumaphos *	Insecticide/OP Insecticide, acaricide/OP
II	moderately hazardous	50–2000	Fipronil * Chlorpyrifos * Imidacloprid * Bifenthrin * Acetamiprid	Insecticide, acaricide/PP Insecticide/OP Insecticide/NN Insecticide, acaricide/PY Insecticide/NN
III	slightly hazardous	>2000	Atrazine * Malathion Glyphosate Tau-fluvalinate	Herbicide/T Insecticide, acaricide/OP Herbicide/OP Insecticide, acaricide/PY
U	unlikely to present acute hazard	>5000	Azoxystrobin	Fungicide/S

* Pesticide currently being withdrawn, already withdrawn or banned in many countries due to toxicity. NN—neonicotinoids, OP—organophosphate pesticide, PP—phenylpyrazoles, PY—pyrethroids, S—strobilurins, T—triazine derivative.

**Figure 3:**
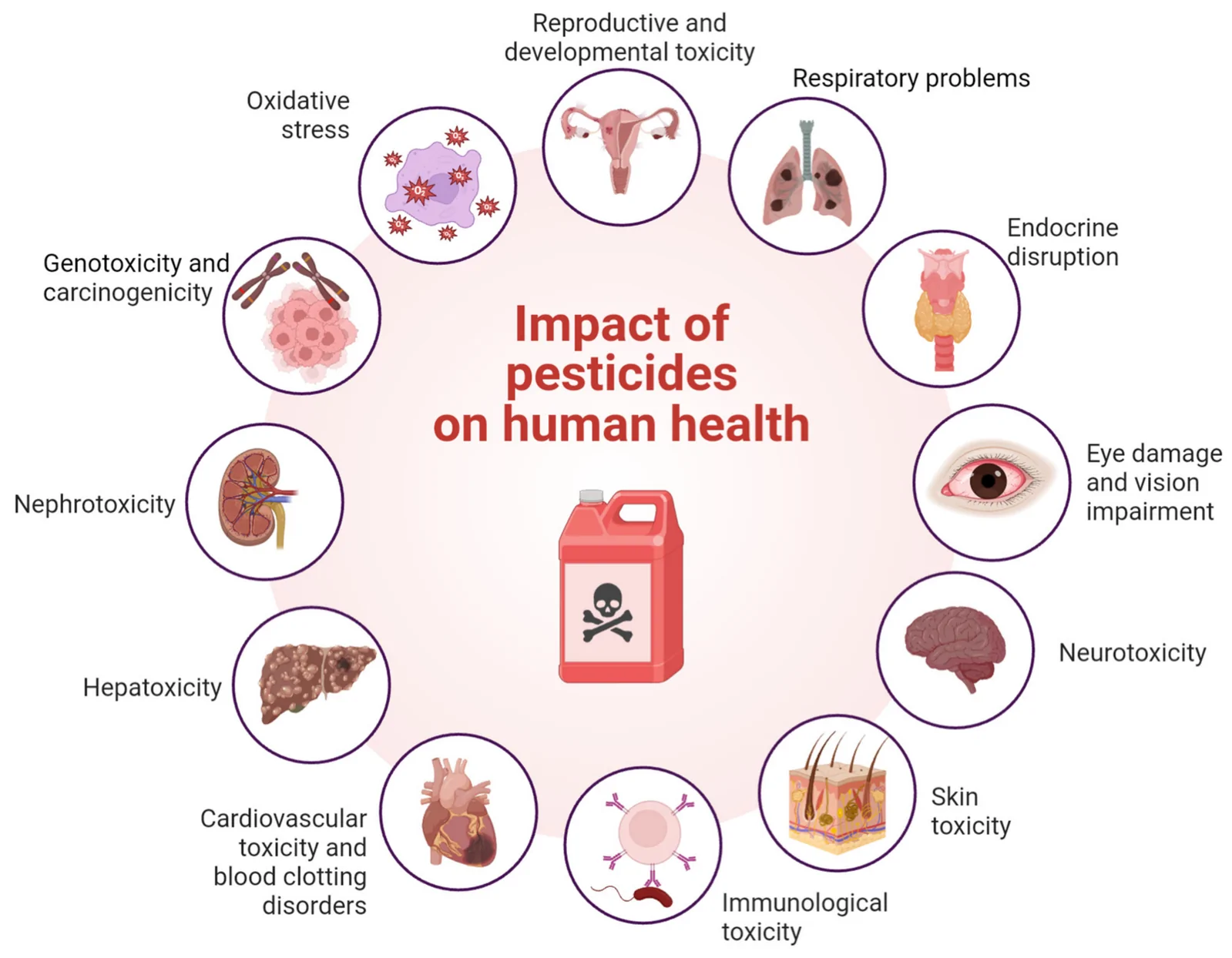
The impact of pesticides on various human organs and systems (based on [44,45,46,56,57,58,59,60,61,62,63,64,65,66], Created in BioRender. Duda, A. (2026) https://BioRender.com/hebs023).

Regardless of their chemical structure or mechanism of action, some pesticides are recognised as being harmful to humans. Exposure through ingestion, inhalation or dermal contact can cause a variety of health problems (Figure 3). These substances can enter the human body indirectly, through contaminated food, or directly—during pesticide application [36]. The same applies to pesticides and bees, pesticide residues can be found in the digestive tracts of bees and, consequently, in their products, such as honey, perga and pollen. The groups at highest risk of direct exposure are farmers and agricultural workers (e.g., those applying sprays), as well as their families and children. Once inside the human body, insecticides are capable of bioaccumulation, biomagnification and bioconcentration [36].

Human exposure to insecticides can lead to various adverse health effects, including skin disorders, congenital defects, malignant tumours, genetic disturbances, respiratory problems, blood clotting disorders, neurological and neurodegenerative diseases, endocrine disruption, coma and even death (Figure 3) [44,45,46]. Numerous studies have revealed the harmful impact of pesticides on human health, proving that they can induce both acute toxicity and chronic disease [40,45,47,48]. Symptoms of acute toxicity commonly include headaches, dizziness, nausea, abdominal pain, diarrhoea, panic attacks and nervous system depression. In severe cases, symptoms can include coma or death. Chronic toxicity, on the other hand, develops following prolonged exposure to pesticides. Symptoms may manifest over an extended period or appear later after initial exposure [40,46]. The type of substance, the dose, the duration and frequency of exposure, exposure to other hazardous substances at the same time, and individual susceptibility all play a significant role in each case.

The nervous system is particularly susceptible to the toxic effects of various chemical compounds, and the mechanisms by which neurotoxic chemicals can act vary. They may: (i) induce myelopathy; (ii) affect neurotransmission; (iii) cause neuropathies; or (iv) act on axons, resulting in axonopathies. The scientific community recognises that neurotoxins, including pesticides, lead to neuronal cell death and degeneration, resulting in irreversible neuropathies. Furthermore, pesticides can induce oxidative stress, calcium overload, and damage to the cytoskeleton and mitochondria and cause alterations in enzyme levels [40,49].

A particularly serious consequence of pesticide exposure is its impact on the human endocrine system [50]. Pesticides can notably affect the female reproductive system, leading to miscarriages, reduced fertility, low birth weight, and preterm birth [51,52]. Pesticides can also exhibit teratogenic effects, disrupting hormone secretion and causing oestrogenic effects or disturbing the hormonal balance by interfering with reproductive hormone pathways (e.g., gonadotropins, FSH, LH) [28,40,53]. Pesticides can also bind to androgen receptors, activate them and mimic hormonal activity, thereby influencing hormone production, transport, and metabolism [54,55,56]. Furthermore, they may damage developing gametes and impair early foetal development. In males, exposure to pesticides can result in reduced testicular mass, decreased testosterone levels, impaired sperm motility, disturbances in steroidogenesis and spermatogenesis, and degeneration of Sertoli cells [40,57,58].

Pesticides (e.g., organophosphates, organochlorine compounds, carbamates and neonicotinoids) have been reported to induce genotoxicity, meaning they can damage genetic material (DNA), as well as alter or disrupt the translation or polymerisation of the proteins and enzymes involved in forming chromosomes and chromatin. Genotoxicity can ultimately lead to genetic mutations [62,64,65], and an increased mutation rate can lead to the development of various cancers [33,59,67,68,69,70], leukaemia and non-Hodgkin lymphoma [61]. It should be noted that, according to the IARC classification, certain pesticides are listed as either Group 2A (probably carcinogenic to humans, e.g., atrazine, malathion and glyphosate) or Group 2B (possibly carcinogenic to humans, e.g., parathion and dichlorvos). In addition, pesticides can induce cytotoxicity, i.e., damage at the cellular level, affecting cellular physiology [45,71,72]. Numerous changes in cellular biochemistry have been observed in the presence of organophosphate compounds, including alterations in malondialdehyde (MDA) and glutathione (GSH) levels, as well as reductions in catalase activity, which manifests in the form of oxidative stress [73]. A negative correlation has been reported between cholinesterase and MDA, and a positive correlation between cholinesterase and reduced glutathione [66]. These findings suggest that organophosphate compounds promote free radical and reactive oxygen species (ROS) generation, as indicated by elevated MDA levels and reduced GSH and catalase levels. ROS can cause further damage to important cellular compounds, leading to disorders of tissues and organs. Pesticides have been proven to exert nephrotoxic [74,75], hepatotoxic [76,77,78,79], immunotoxic [60], and respiratory dysfunction [63]. They have also been shown to increase the risk of Alzheimer’s disease [80,81], Parkinson’s disease [82,83,84], and diabetes [85,86,87], as well as to promote the development of antibiotic resistance in microbial communities [61]. The negative impact of pesticides is not limited to humans, but extends to other animals including, of course, honey bees. It has been demonstrated that pesticides cause increased mortality in honey bee colonies and reduce the populations of other pollinators [88,89,90].

It is estimated that, in recent years, more than 25 million agricultural workers worldwide experience accidental pesticide poisoning each year. According to Rana et al. [49], approximately 44% of the pesticides used globally each year are insecticides, 30% are herbicides, 21% are fungicides, and 5% belong to other pesticide categories. Modern agriculture and national governments are unlikely to completely abandon the use of pesticides or to prohibit them. However, to ensure the sustainable development of agriculture, protect the environment, and safeguard human health, research efforts should increasingly focus on developing and implementing biopesticides [40]. An alternative to using pesticides in agriculture is organic farming. This is based on the principles of sustainable development, rural area advancement, animal welfare, and food safety. Consequently, there has been a growing global consumer interest in products originating from organic cultivation and livestock production [91]. In the European Union, Regulation (EU) 2018/848, with subsequent amendments [92], defines the rules governing the production and labelling of organic food. This document also specifies requirements for apicultural practices, including feeding the bees and using veterinary medicinal products. According to the regulation, apiaries should be situated at a sufficient distance from potential sources of contamination of bee products or poor bee health. Moreover, an organic apiary should be situated in such a way that the sources of nectar and pollen within a 3 km radius consist essentially of plants grown using organic methods or natural vegetation. Alternatively, the sources may consist of crops treated only by methods with a low environmental impact, as set out in Articles 28 and 30 of Regulation (EU) No 1305/2013 [93], which must not endanger the eligibility of beekeeping as an organic activity. These requirements do not apply to areas where there is no flowering or to dormant hives. We should remember, however, that bees can fly over long distances, so it cannot be entirely excluded that organic bee products may still contain pesticide residues. The half-life of various pesticides and their metabolites, which can reach several decades, is also a significant factor. This means that pesticides can still be detected in food even if they have been withdrawn from the market for many years and are no longer in use.

The EU regulations on maximum residue limits (MRLs) for pesticides apply only to honey, with limits ranging from 0.01 to 1 mg/kg, and there are no specific guidelines for other apicultural products [94]. The global average annual consumption of honey stands at 0.43 kg per capita per year [95], which translates to a daily consumption per capita of 11.78 × 10^−4^ kg of honey. Assuming an average body weight of 70 kg, the average daily intake (AvDI) of honey is 16.8 × 10^−6^ kg/kg of body weight. Taking into account the oral reference dose (RfD) values provided by the U.S. EPA, the FAO, and the European Commission, as well as the AvDI of honey and other bee products and the highest reported concentrations of individual pesticides in the literature (see Table A2 in Appendix A.2), it was demonstrated that the hazard quotient (HQ) is lower than 1 for the vast majority of the analysed pesticides. The exceptions are carbaryl, coumaphos and phosmet, which are present in bee pollen; the respective HQ values for these are 2.62, 2.32 and 6.61 respectively. Since the consumption of bee pollen is almost exclusively limited to individuals undergoing apitherapy, it can be concluded that the consumption of honey and bee products poses a low risk to consumer health.

In contemporary times, both producers and consumers have become more aware of the harmful effects of excessive pesticide usage in agriculture and horticulture on the health of bees, humans and other living organisms. Pesticide residues have been identified in nearly all types of honey and other bee products. In a recent review, Sharma et al. [14], summarised data on the occurrence of various pesticides, including insecticides, acaricides, fungicides, herbicides, and neonicotinoids in honey, propolis, bee bread, and pollen samples collected from different countries around the world. The study found that all bee products, except royal jelly, were primarily contaminated with bifenthrin, triadimefon, lambda-cyhalothrin, and chlorpyrifos.

The development of modern detection methods and the need to routinely monitor the environment, living organisms and food for contamination enabled the identification of bees and bee products as potential bioindicators of the presence of pesticides in the environment. In the experiments by de Oliveira’s team [96], a GC-MS/MS method was developed and validated to determine the residues of 26 different pesticides in bee pollen, in accordance with the SANCO recommendations from the European Union. Environmental monitoring was performed by analysing 145 pollen samples collected from ten hives at the Embrapa experimental apiary in Jaguariúna (São Paulo State, Brazil). The selection of the 26 analysed pesticides was based on ANVISA and PNCRC reports, as well as surveys of neighbouring farms where the sporadic use of abamectin, carbendazim, mancozeb, spirodiclofen, imidacloprid, thiophanate-methyl, dimethoate, beta-cyfluthrin, glyphosate, deltamethrin, and 2,4-D (2,4-dichlorophenoxyacetic acid) was reported. The study demonstrated that the GC-MS/MS method with an ion trap enables accurate and precise assessment of pesticide concentrations in bee pollen. The authors emphasise that proper sample preparation is crucial for diagnostics. Sorption methods have shown that pesticides accumulate in bee pollen. Bioallethrin and pendimethalin were among the substances detected in pollen, identified in four and eighteen samples, respectively, at concentrations below 25 ng/g. Furthermore, for malathion, abamectin, and aldrin, a direct correlation was observed between the affinity of the pesticides for bee pollen and their octanol–water partition coefficients. These results may indicate which pesticides are most likely to accumulate in bee pollen, thus confirming its use as a bioindicator of environmental pollution by various pesticides. Similar studies on pesticide residue contamination in bee pollen were conducted by Carrera’s team [97]. Thanks to the optimisation of the comprehensive LC-MS/MS method, 41 pesticides were identified in 34 samples of bee pollen.

Researchers have detected pesticides or their residues in honey bees and their products at varying concentrations. Pesticides tend to accumulate in bees’ bodies in greater amounts than in the honey they produce. It is thought that bees fulfil a protective function (biobarrier) against pesticide contamination and other toxic pollutants, which explains the lower concentrations detected in bee products [98,99].

It should be noted, that the contamination of honey and bee products with pesticides may result from agricultural spraying with substances that are highly persistent pollutants. Some of these compounds, such as polychlorinated biphenyls (PCBs), remain in the environment even though their production ceased 45 years ago. They can contaminate plants and, consequently, bees, honey, and other bee products. PCBs have been identified at various concentrations in honey bees, honey, pollen, and beeswax [100,101,102]. The most frequently identified insecticides in honey from European countries were organochlorine (OC) compounds, such as lindane and its isomers, hexachlorocyclohexane (HCH), aldrin, dieldrin, endrin, DDT isomers, heptachlor and heptachlor epoxide, methoxychlor, and endosulfan. Similar to PCBs, many OCs are no longer used in agriculture, yet they still persist in the environment. In addition to organochlorine compounds, organophosphorus (OP) pesticides, such as dialifos, trichlorfon (metrifonate), and dichlorvos, as well as carbamates, have been detected in bee products [14,100].

In horticulture and agriculture, insecticides can be applied to protect crops, as well as to control bee diseases and pests. Their use is not always controlled and does not always follow approved protocols. The application of insecticides such as celazole, acaricides (amitraz), coumaphos, bromopropylate, tau-fluvalinate and flumethrin is particularly hazardous. These insecticides are used to control the fungus that causes ascospheriosis (*Ascosphaera apis*) and the *Varroa destructor* mite, which is responsible for the most severe bee disease, varroosis [44,89,103]. Preparations used to control these pathogens accumulate in beeswax, pollen, and bee bread [104,105].

Seed coatings can contain neonicotinoid insecticides, used to control bee parasites, which often exceed permissible limits. Neonicotinoid compounds exhibit high affinity for nicotinic receptors located on cholinergic neurons and affect the brain of honey bees as well as their immunity [106]. Acaricides, which are effective against the mite *V. destructor* mite, can also negatively impact bee reproduction, olfactory functions, and honey production. Amitraz and fluvalinate are commonly used to combat this mite, but they can reduce honey yield, impair colony reproduction, and negatively affect bees’ learning and memory [107].

Due to their health benefits, bee products are becoming increasingly popular among consumers, hence demand for these products is growing on global markets. That is why honey adulteration and the sourcing of products from contaminated areas are becoming increasingly common. It is the fundamental responsibility of beekeepers to produce bee products that protect public health. To prevent pesticide residues in bee products, the use of insecticides should be limited during flowering periods and prohibited entirely during honeybee foraging flights. Programmes for monitoring honey should be developed and implemented to ensure that consumers can safely use honey and other bee products [108].

Another bee product that is widely used by health-conscious consumers due to its health-promoting properties is bee pollen [109]. Unfortunately, it may also contain pesticides and other substances. As pollen is the primary source of nutrients for bees, its contamination with pesticides affects bee exposure to these harmful substances [110]. The pesticide content of bee pollen can vary widely depending on the botanical origin of the sample, the geographical region, the method and timing of pesticide application, the doses applied, the physicochemical properties of the active substances, and environmental conditions (temperature, humidity, water activity, and soil properties), which can change even between seasons [19,25,111,112,113]. The most frequently detected active pesticide compounds in bee pollen are chlorpyrifos, fluvalinate, carbendazim, coumaphos, and thiacloprid [111,114,115]. Trifloxystrobin, coumaphos, chlorpyrifos-ethyl, boscalid, myclobutanil, mandipropamid, amitraz, dimethomorph, fenhexamid and cyprodinil were mainly present in bee pollen samples collected in European countries. In contrast, imidacloprid, thiamethoxam, fenpropathrin, bifenthrin, chlorpyrifos, chlorothalonil, boscalid, myclobutanil, coumaphos, fluvalinate, carbendazim, carbaryl, azoxystrobin, phosmet and triadimefon were found in higher concentrations in samples from non-EU countries [19,111,114,115,116,117,118]. More than 350 pesticides were identified in pollen samples from various countries worldwide [112,119]. It should be highlighted that approximately 85% of bee pollen samples, from both conventional and organic apiaries, were found to be contaminated with pesticides. Furthermore, pesticide concentrations exceeded the maximum residue limit (MRL) in 40% of samples [112]. In seven samples of Polish bee pollen harvested between August 2018 and July 2019, substances exceeding MRLs were detected, including: chlorpyrifos (detected in 85.7% of samples), azoxystrobin (28.6%), biphenyl, boscalid, cypermethrin, cyprodinil, fenvalerate, tebuconazole, and propamocarb hydrochloride [25]. Similar results were obtained for Brazilian bee products [120]. More than 33% of the tested bee products were contaminated with at least one pesticide. The most frequently detected chemical classes were organophosphates (67 cases), chlorinated organic compounds (35 cases), and neonicotinoids (30 cases), with the most frequently detected compounds being monocrotophos, imidacloprid and methomyl. The concentration of fipronil reached 2.099 mg/kg, meaning that it was 400 times higher than the European Food Safety Authority’s maximum residue limit (0.005 mg/kg) for honey.

Rondeau and Raine [121] prepared a report addressing the risk to the health of honey bees from exposure to fungicides. They analysed 76 scientific publications and found that residues of fungicides had been reported in honeybee hives. Furthermore, the presence of 90 different fungicides and their metabolites was reported in honeybee bodies, as well as in bee products, including honey, bee bread, pollen, and beeswax. The risk posed to honey bees by fungicide residues using the EPA risk quotient (RQ) method was estimated. The results obtained indicated that none of the fungicides exceeded the Level of Concern (LOC) established by regulatory agencies for acute risk. However, concentrations of three fungicides (flutriafol, pyraclostrobin, and vinclozolin) exceeded the chronic risk threshold established by the European Food Safety Authority (EFSA) for honey bees, and twelve exceeded this threshold for bumble bees and solitary bees.

According to various studies [111,122,123], pesticide residues in bee pollen generally do not pose a chronic risk to consumers, but they may cause acute pesticide poisoning. Therefore, it is important to emphasise the need for quality and safety regulations for this product at a national legislative level once again. Such regulations are already being introduced, with the most hazardous active substances in plant protection products being gradually withdrawn from the market. For example, chlorpyrifos was used as an organophosphorus insecticide, posing risks to bees and humans, and has therefore been withdrawn from use in the EU (Commission Implementing Regulation 2020/18/EU). Fluvalinate (tau-fluvalinate), an acaricide used in beekeeping that can be detected in bee products, is one of six active substances (along with fluazinam, fluopyram, diflufenican, mefentrifluconazole and flonicamid) for which the Danish Environmental Protection Agency decided to withdraw approval by July 2025 [124]. Carbendazim, a fungicide that inhibits cell division, has been withdrawn from the EU market (Commission Implementing Regulation 542/2011/EU) [125], as has the carcinogenic thiacloprid (Commission Implementing Regulation 2020/23/EU) [126]. Moreover, international standards have been established to regulate the production, quality, and testing of royal jelly (ISO 12824:2016 [127]), bee propolis (ISO 24381:2023 [128]), and pollen (ISO 24382:2023 [129]).

According to a study by Végh et al. [19], royal jelly is the least contaminated bee product, probably as a result of the filtration by nurse bees during secretion. On the other hand, various pesticides can accumulate in bee bread, propolis and beeswax, reaching concentrations of over 10^3^ μg/kg. Analysis of 25 beeswax samples from the Extremadura region of Spain, a key beekeeping area, revealed the presence of 18 insecticides, seven fungicides and four herbicides [130]. These included compounds that are not authorised for use, such as endrin (up to 218.57 µg/kg) and chlorfenvinphos (15–16 µg/kg). The compounds most frequently identified in beeswax were acrinathrin (5.27–122 µg/kg) and tau-fluvalinate (6.5–138.79 µg/kg). Therefore, beeswax can act as a product that accumulates pesticide residues and subsequently contaminates honey. Consequently, it is essential to monitor pesticide residues in commercial hive wax and assess their impact on bee health. With regard to human health, it is worth noting that beeswax is approved in the European Union as a safe glazing agent (E901) and that the amount consumed is negligible. In industrial production, only 450 g of wax is needed to coat 100,000–160,000 apples, meaning each fruit contains just one or two microscopic drops of the substance. Furthermore, if the consumer washes the fruit before eating it, the intake of beeswax is reduced to zero. Therefore, this bee product was omitted from the HQ calculation, as its average intake is negligible in terms of the pesticides it contains.

The presence of pesticide residues in honey depends on the plants that the bees collect pollen from. In the Czech Republic, almost 90 samples of various types of honey, including pine, multifloral, sunflower, acacia, linden and rapeseed, were examined for the presence of pesticide residues [131]. The authors found no residues of the tested pesticides in pine honey. The hazard quotient (HQ) value for pine honey was 0, indicating that it is the safest for consumption. The most frequently detected pesticides in other honey types were chlorpyrifos (ranging from 15.1 µg/kg in linden honey to 22.3 µg/kg in multifloral honey), clothianidin (from 12.0 µg/kg in acacia honey to 22.0 µg/kg in rapeseed honey), dimethoate (from 8.9 µg/kg in multifloral honey to 18.9 µg/kg in rapeseed honey), and thiamethoxam (from 4.2 µg/kg in linden honey to 15.6 µg/kg in rapeseed honey). The authors calculated the estimated daily intake (EDI) based on the assumption that the average European adult consumes 0.828 kg of honey per year and has an average body weight of 70.8 kg. The lowest EDI was found for acacia honey (1.28 × 10^−4^ mg/kg body weight per day), while the highest EDI was calculated for rapeseed honey (2.65 × 10^−4^ mg/kg bw per day). The HQ values calculated by the authors (EDI/ADI, where ADI is the acceptable daily intake) were all lower than 1, indicating that none of the honeys pose a health risk to humans.

It should be remembered that pesticide concentrations below the legal limit may still pose a health risk due to their potentially synergistic toxicity. This is because honey rarely contains just one pesticide; several or even several dozen are usually present. Moreover, consumers do not solely consume honey. Many of the fruits and vegetables that form part of a typical diet may also be contaminated with pesticides. The level of contamination depends heavily on the country of origin of a given product and the applicable legal regulations (limits). Among the reports of limit exceedances submitted to the RASFF, products from Turkey and India were the most frequently mentioned. More than half of the reports regarding pesticide levels are classified as grounds for rejection at the border. The most frequently reported pesticides were chlorpyrifos, dimethoate, carbendazim and omethoate [132]. The most contaminated raw materials include sweet peppers (22.4%), spinach, kale, collard greens and mustard greens, as well as strawberries, grapes, peaches, cherries, nectarines, pears, apples, blackberries and blueberries.

Therefore, when assessing the risk, it is necessary to consider not only data on pesticide residue levels in honey and bee products, but also in other ingredients used in meal preparation, and calculate the hazard index (HI), which is the sum of the HQs of the chemicals in a mixture (diet). Undoubtedly, the presence of pesticide residues in bee products lowers their quality and diminishes their health-promoting properties or may even have a negative impact on human health. Moreover, the fact that a given dose of pesticides does not have a harmful effect on humans does not mean that it is not toxic to bees, even at low exposure levels. Therefore, there is an urgent need to continuously monitor pesticides in various bee products and expand the European Union’s pesticide database to include a wider range of such products [25].

### 2.2. 5-Hydroxymethylfurfural (5-HMF) and Furfural

In addition to pesticide residues, bee products may contain other harmful compounds, such as furfural (with a reference dose RfD of 3 × 10^−3^ mg/kg bw/day) and 5-hydroxymethylfurfural (5-HMF). As early as the last century, studies indicated that they induced a significant number of chromosome aberrations and a significant reduction in mitotic activity in cultured Chinese hamster V79 cells [133]. Although the mutagenic and carcinogenic effects of HMF itself have not conclusively been confirmed, further studies indicate that its metabolite, 5-sulfooxymethyl-2-furaldehyde (SMF), formed under the influence of sulfotransferases (SULTs), may exhibit genotoxic and mutagenic effects. SMF may cross-link DNA in target cells, thereby exerting its toxic effects. The mutagenicity of SMF could be further enhanced by the addition of extra chloride ions to the assay medium, resulting in the formation of 5-chloromethylfurfural (CMF). CMF was more mutagenic and cytotoxic in bacteria than SMF [134].

There is no widely established oral reference dose (RfD) for 5-HMF in the US EPA or the IRIS databases, but the Codex Alimentarius standard and European legislation (Council Directive 2001/110/EC [135]) set a maximum residue level (MRL) at 40 mg of 5-HMF per kg of honey and 80 mg/kg for tropical honeys. Prolonged or excessive intake of these compounds is believed to cause many side effects, including membrane irritation, nausea, liver and kidney disorders, headaches, weakness, and central nervous system disorders, in addition to leading to mutations and cancer development [136,137,138].

5-HMF is primarily produced during the Maillard reaction and is naturally present in many carbohydrate-rich products with a low pH, especially those containing fructose, such as honey. As 5-HMF forms mainly through exposure to high temperatures or prolonged storage, its presence in honey can be considered an important indicator of honey quality, freshness, and adequate processing [139]. In fresh honey, the level of 5-HMF is usually negligible, but overheating (above 90 °C) or prolonged storage at room temperature (25 ± 2 °C) can significantly increase its concentration [113,140]. Honey produced in subtropical regions may also contain higher levels of 5-HMF without having been overheated or adulterated, solely due to the naturally high local temperatures [141]. Fresh honey samples from Malaysia stored for 3–6 months had 5-HMF levels ranging from 2.80 to 24.87 mg/kg, meeting the requirements of Council Directive 2001/110/EC for tropical honeys. However, the same honey samples stored for 12–24 months contained much higher 5-HMF concentrations (128.19–1131.76 mg/kg) [142].

The presence of furfural and 5-HMF has been quantified in various Polish bee products, including honey, bee bread and pollen [143]. The highest concentration of furfural was detected in bee bread sourced from north-eastern Poland, while the highest 5-HMF level was found in bee bread from central Poland, probably due to varying storage times and conditions. In all these samples, the content of 5-HMF was below the limit of 40 mg/kg. On the other hand, among 26 samples of honey of various botanical origins purchased from European shops, honeys from France and the majority from Spain met the requirements for 5-HMF content. In contrast, for honeys from Poland, Italy, and Slovakia the level of 40 mg/kg established for saleable honey was exceeded in most cases [144]. The highest reported content of 5-HMF in Slovakian honeydew (678.77 mg/kg) exceeded the permissible level by a factor of 17. Many other studies have demonstrated that the level of 5-HMF in honey depends on the plant from which it was produced. High 5-HMF levels were reported, among others, in orange blossom honey (54.47 mg/kg), wildflower honey (15.06–82.63 mg/kg) and acacia honey (2.31–103 mg/kg) [145], whereas low levels were demonstrated in linden honey (0.95 mg/kg), buckwheat honey (1.72 mg/kg) and rapeseed honey (0.86 mg/kg) [146], as well as in chestnut (n.d. −4.1 mg/kg) and acacia honeys (8.4–16.2 mg/kg) [147].

To summarise the presented data, we can estimate the actual health risk to consumers using the tolerable daily intake (TDI) of 2 mg/kg body weight/day instead of the non-existent reference dose. Assuming that an adult weighing 70 kg consumes 1.178 g of honey per day (see Appendix A.2.2), with an EU-permitted 5-HMF concentration of 80 mg/kg (the limit for tropical honey), the average daily intake of 5-HMF would be 0.09424 mg (1.178 × 10^−3^ kg of honey × 80 mg HMF/kg). Converting this to body weight yields an EDI of 0.001346 mg/kg bw/day (0.09424 mg 5-HMF/70 kg). This means that HQ is less than 1 (0.001346/2 = 0.000673). The HQ will remain below 1 even when considering the highest 5-HMF concentration detected in honey (1132 mg/kg) and body weights of 5 kg (infant) or 12 kg (child up to 3 years of age). Therefore, honey itself does not pose a health risk to consumers, even if it is contaminated with 5-HMF. However, it is important to bear in mind that 5-HMF is present in much higher concentrations in products that we consume far more frequently, such as bread, biscuits, coffee, chips, etc.

### 2.3. Polycyclic Aromatic Hydrocarbons

Another class of organic compounds that contaminate honey and other bee products are polycyclic aromatic hydrocarbons (PAHs), which are composed of multiple fused aromatic rings. These toxic pollutants are primarily generated by the incomplete combustion of fuels or organic biomass, as well as during various industrial activities, such as vehicle exhaust, gas-fired or oil-fired heating, incinerators, and industrial emissions. As they are highly persistent and can accumulate in the food chain, PAHs constitute an important health problem.

Current EU regulations do not set a maximum permitted level of PAHs in honey. However, with regard to supplements containing propolis or royal jelly, it has been established that the concentration of PAH4 (the sum of benzo[a]pyrene, benzo[a]anthracene, benzo[b]fluoranthene, and chrysene) must not exceed 50 μg/kg, with a maximum of 10 μg/kg for benzo[a]pyrene alone [148]. Some published results show that these limits may be exceeded. For example, the sum of PAHs in Polish bee products, such as bee bread and pollen, ranged from 324.0 to 866.4 μg/kg, with benzo[a]anthracene and chrysene being detected in the samples [143]. The authors concluded that the risk of cancer was acceptable at typical levels of honey consumption containing a specific concentration of PAHs. However, they also found that high levels of PAHs in bee bread and pollen may increase the risk of cancer if consumed regularly.

Three beehive matrices were sampled from various apiaries in western France in 2008 and 2009 and analysed for the presence of PAH4 [26]. Studies revealed that the PAH4 concentrations were significantly affected by the landscape context. The samples of honey contained the lowest levels of PAH4 (0.03–5.80 μg/kg, mean = 0.82 ± 1.17 μg/kg), higher levels of PAH4 contamination were observed for bee samples (0.32–73.83 μg/kg, mean = 7.03 ± 17.83 μg/kg), and the highest for pollen samples (0.33–129.41 μg/kg, mean = 7.10 ± 22.28 μg/kg). The concentration of PAHs analysed in honey samples from different regions in Italy ranged from values below the limit of detection (LOD) to 35 μg/kg, depending on the harvest year and region [149].

The systematic review analysed data from 29 studies published between 2000 and 2025 that examined the concentration of the sum of polycyclic aromatic hydrocarbons (ΣPAHs) in honey [16]. The tests did not confirm any correlation between the ΣPAHs content and the type of flower from which the honey originated. There were also no differences between monoflower and multiflower honeys. As predicted, ΣPAHs levels depended on the geographic region of the honey’s origin, with the highest levels observed in urban and industrial areas. Samples from Lebanon were characterised by a mean ΣPAHs level of 166.83 μg/kg (based on QuEChERS–SPME extraction combined with GC–MS/MS detection). European honeys generally exhibited lower ΣPAH levels (e.g., contaminations in Italian and Austrian honeys were reported at ~0.1 μg/kg and, in some cases, was even below the limit of detection), while Turkish honeys were moderately contaminated (with ΣPAHs ranging from 1.71 μg/kg to 10.12 μg/kg). Most researchers analysed the 16 PAHs prioritised by the US Environmental Protection Agency (EPA), with the most toxic compound, benzo[a]pyrene, being the most frequently detected. Other PAHs frequently identified in the tested honeys included chrysene, pyrene, fluorene, phenanthrene, benzo[a]anthracene, naphthalene, anthracene, fluoranthene, and benzo[b]fluoranthene. In 19 of the 29 studies (65.5%), especially in samples from urban or industrial regions in Africa, Latin America, and parts of Asia, the cancer risk (CR) was greater than 1 × 10^−4^, and these samples were therefore classified as ‘at risk’. However, it should be emphasised that the PAHs levels analysed in these studies were determined using different methods with varying sensitivity. Based on our assumptions about the average daily intake of honey, bee bread, and bee pollen (Appendix A.2), the HQ calculated was still less than 1, even at the highest observed PAH concentrations in these products.

### 2.4. Residual Antibiotics

In the US and Canada, only three antibiotics—oxytetracycline, tylosin, and lincomycin—are approved for use in honey bees [150]. Commission Regulation (EU) No 37/2010 on pharmacologically active substances [151] sets their maximum residue limits (MRLs) in foodstuffs of animal origin, but these limits have not yet been established for beekeeping products. This means that their use is not regulated for the prevention and treatment of diseases affecting honey bees; therefore, a ‘zero tolerance’ approach is required, and honey containing antibiotic residues is not permitted to be sold in Europe. This regulation also contains a list of pharmacologically active substances that are prohibited, such as: chloramphenicol, chloroform, chlorpromazine, colchicine, dapsone, dimetridazole, metronidazole, nitrofurans, and ronidazole [151]. However, some countries, such as Switzerland, the United Kingdom, France, and Belgium, have set their own limits for antibiotics in honey (the level above which a sample is considered non-compliant), which are typically between 0.01 and 0.05 mg/kg for each group of antibiotics [152,153]. Regulation (EU) 2019/6 of the European Parliament and of the Council stipulates that antibiotic medicinal products should not be used prophylactically, except in exceptional cases, and only for administration to individual animals [154]. In this regard, it is generally accepted that the use of antibiotics to treat bee diseases is not permitted in most European countries. Nevertheless, numerous studies have reported antibiotic residues in honey and other bee products, applying not only to samples of honey imported from other regions (China, Chile, and the Americas) but also to samples of European origin. For example, tetracycline-derived residues were found in 29% of Greek honeys samples examined, with 20.3% of these containing more than one of the tested derivatives [155]. While the majority of samples had residues ranging from 18 to 55 μg/kg of honey, some samples were contaminated at levels exceeding 100 μg/kg. Although no maximum residue limit (MRL) has been established for honey, EU Regulation 37/2010 [151] specifies MRLs for tetracyclines (including tetracycline, oxytetracycline, and chlortetracycline) in the range from 100 µg/kg (in milk and muscle) to 600 µg/kg (in kidneys). Unlike MRLs, which apply to legally approved veterinary drugs, reference points for action (RPA) are established for antimicrobial agents that are prohibited due to their carcinogenic or highly harmful effects, for which a safe limit cannot be calculated (EU Regulation 2023/411) [156]. Such substances, which are prohibited in food-producing animals, include chloramphenicol (RPA = 0.15 μg/kg), nitrofurans and their metabolites (RPA = 0.5 μg/kg for each substance), and malachite green and leucomalachite green (RPA = 0.5 μg/kg for their sum). Exceeding the RPA level in a product means that it is deemed non-compliant with EU law, which results in the entire batch of such goods being detained and withdrawn from the market to prevent it from reaching consumers, after which it is destroyed. In addition, the regulatory authority notifies the Rapid Alert System for Food and Feed (RASFF), alerting other member states. Veterinary services then conduct an investigation to determine the origin of the prohibited substance in the product (whether it was intentionally added or resulted from cross-contamination).

Of the 75 honey samples purchased in Switzerland and analysed, 13 (17%) were found to contain residues of antibiotic chloramphenicol (CHL). This was particularly true of honeys containing Asian honey admixtures (38% of samples tested were positive), indicating that CHL is still being used in some Asian countries and that these honeys should not be introduced into the European market, as they exceed the specified RPA values. CHL concentrations in these honey samples ranged from 0.4 to 6.0 μg/kg, with only six samples not exceeding the Swiss MRL [157]. Similar results were obtained when honey from the Belgian market was analysed. Very few samples of honey produced by Belgian (mainly Flemish) beekeepers contained residues of the three veterinary drugs: streptomycin in four out of 248 samples (1.6%), tetracycline in two out of 72 samples (2.8%), and sulfonamides in three out of 72 samples (4.2%). No residues of β-lactams (50 samples) or chloramphenicol (93 samples) were found [158]. However, antibiotic residues were quite often detected in imported honey: streptomycin in 51 out of 108 samples (47.2%), tetracyclines in 29 out of 98 samples (29.6%), sulfonamides in 31 out of 98 samples (31.6%), and chloramphenicol in 40 out of 85 samples (47.1%). The most frequently detected antibiotics in the analysed samples of Turkish honey were: dihydrostreptomycin (levels ranging from 4.40 to 76.66 μg/kg), streptomycin (from not detected to 6.20 μg/kg), erythromycin (from not detected to 358.11 μg/kg), sulfadimidine (up to 19.64 μg/kg), and enrofloxacin (in two samples, from 4.85 and 7.03 μg/kg) [159]. As with tetracycline, there are no MRLs for honey and bee products for other antibiotics. For comparison, the MRLs in milk were set at 4 μg/kg for ampicillin, 40 μg/kg for erythromycin, 100 μg/kg for sulfonamides, enrofloxacin, gentamicin, and for the sum of enrofloxacin and ciprofloxacin, 150 μg/kg for kanamycin, and 200 μg/kg for streptomycin.

According to Rodrigues et al. [153], the following classes of veterinary pharmaceuticals residues were detected in bee products between 2002 and 2023: sulfonamides (sulfadimethoxine, sulfadimidine, sulfamethazine, and sulfathiazole), nitrofurans (the metabolite nitrofurazone), aminoglycosides (streptomycin and dihydrostreptomycin), macrolides (tylosin and erythromycin), lincosamides (lincomycin), nitroimidazoles (metronidazole), quinolones (enrofloxacin and ciprofloxacin), and tetracyclines (oxytetracycline), as well as chloramphenicol residues. The analysed honey samples originated from various countries, including Argentina, Australia, Chile, China, Slovakia, Spain, Hungary, India, Israel, Italy, Lithuania, Poland, Portugal, the Czech Republic, Romania, Turkey, and Ukraine.

Antibiotics were also detected in 84.0% of honey samples from China, with two or more antibiotics being present in 56.2% of samples simultaneously. The predominant compounds were quinolones (69.1% of honey samples), sulfonamides (44.7%), tetracyclines (26.6%) and phenicols (1.1%). The median antibiotic residue concentration was 0.09 mg/kg, with higher concentrations detected in cheaper honeys and those from non-commercial crops [160]. Residues of sulfadimethoxine, tylosin A, tylosin B, and sulfamethazine were detected in 23%, 6%, 9%, and 6% of the Canadian honey samples, respectively, and the levels were below the regulatory limits in Canada [161]. Residues of methacycline, oxytetracycline, tetracycline, and its metabolite, 4-tetracycline, were detected in the honey samples examined by Yang [162]. Among 49 samples of honey from Georgia, only two contained streptomycin (at concentrations of 117 and 35 μg/kg), and one contained gentamicin C1 (32 μg/kg) [163]. Nectar and honey samples from the southern part of Tamilnadu (India) contained streptomycin (4–17 and 11–29 µg/kg), ampicillin (2–29 and 3–44 µg/kg), and kanamycin (17–34 and 26–48 µg/kg), respectively [164]. High-quality honey in terms of antibiotic residue content was demonstrated in the tested samples from Brazil [165] and Portugal [166]. Among 55 samples of honey purchased from local markets, only three (with two containing honey from non-EU countries) were positive and contained sulfonamides (sulfamethazine and sulfathiazole) and tetracyclines (oxytetracycline and tetracycline) at concentrations lower than 2 μg/kg [167]. Mahmoudi et al. [168] used the ELISA (enzyme-linked immunosorbent assay) method to determine antibiotic residues in 135 honey samples from the Qazvin province in Iran. Enrofloxacin was the most frequently detected antibiotic (20.7% of samples), with the highest concentration being 10.8 μg/kg. Penicillin (4.4 μg/kg) and chloramphenicol (0.1 μg/kg) placed second and third, respectively. The authors showed that honey samples collected in autumn had the highest level of antibiotic residues (71.85%).

The presence of antibiotic residues in honey is primarily the result of poor beekeeping practices. The main causes of residues of antibiotics or other antibacterial substances in bee products include non-compliance with withdrawal periods, incorrect dosing, unauthorised or illegal use of antibiotics by beekeepers, and improper application of cleaning and disinfection agents [169]. Supporting evidence for these assertions can be found in the results obtained by Kuliçi et al. [170], who investigated the knowledge of Albanian beekeepers. They found that a high proportion of respondents (56.6%) used antibiotics to treat bees. Almost 40% of those surveyed had very limited knowledge of antibiotics and how to use them, admitting to using them without following the instructions on the label. Similar conclusions were drawn by Wang et al. [160], who reported that levels of antibiotic residue in honeys of different origins depended on the production location, retail price, and commercial category of the nectar plants. The researchers found that antibiotic residue levels were higher in honeys from less developed regions of China, which may be associated with lower awareness of safe antibiotic use and weaker management of antibiotic application in apiculture. Honey samples from other countries were also found to contain antibiotic residues. Residues were detected in certain honeys produced in Eastern Europe, as well as in honeys imported from India that were contaminated with prohibited antibiotics (nitrofuran and chloramphenicol). The presence of tetracycline, streptomycin, and ciprofloxacin was also confirmed [153]. These findings suggest a serious potential health risk to consumers, given that antibiotics are often misused, and administered at incorrect times and dosages, or used to treat inappropriate conditions. Therefore, it can be concluded that the key issue lies in beekeepers’ insufficient awareness of the risks associated with antibiotic use and their lack of adequate knowledge about it. As antibiotics are still used in many countries around the world to treat bacterial diseases in apiculture, it is also important to educate honey sellers on the potential consequences of blending cheaper Chinese honey with honey from European apiaries, as this can significantly impact its quality and safety for consumers. Therefore, to minimise the risk of exposure to antibiotic residues, it is advisable to choose honey from a known origin and a reliable, tested source. Ideally, this should be from Europe, as the food quality regulations in force there help to ensure appropriate standards.

Mosca et al. [171] conducted interesting research that demonstrated the presence of high concentrations of oxytetracycline (OTC) in honey, even eight weeks after the antibiotic was last administered to honey bees. Remarkably, OTC residues were also detected in honey samples collected from colonies that had not been treated with the antibiotic (the control group). The authors suggested that this was likely the result of antibiotic transfer by honey bees from treated to untreated hives, indicating cross-contamination. This study also showed for the first time that administering antibiotics to honey bees can lead to environmental contamination, particularly of flowers, during foraging activity. In fact, OTC residues were detected in flowers located a considerable distance from the apiary, even nine days after the last administration of antibiotics to the bees [171]. In other study [172], tetracycline hydrochloride (TC) was artificially introduced into hives three times: in a syrup (0.5 g TC/L, group S) or in powdered sugar (0.5 g/10 g, group P). The control group (C) consisted of hives that were not exposed to TC and were located 20–45 metres from groups S and P. An additional control group (DC) consisted of hives that were not exposed to TC and were located 3 km away. Honey samples were collected at various times from all hives and were analysed immediately or stored in the laboratory at temperatures of 4, 20, or 35 °C. The day after the final TC application, the average TC concentration in honey collected from the brood chamber was 10 times higher in group S (40.7 mg/kg) than in group P (4.34 mg/kg). Interestingly, after eight days, TC residues were detected in all hives, including those in group C. This suggests that they were contaminated with TC as a result of drift. Notably, no residues were detected in any of the hives in group DC at any point during the study, confirming that maintaining an adequate distance protects against cross-contamination. After 146 days, TC was still present in the collected honey (at concentrations of 1.54 mg/kg, 0.35 mg/kg, and 0.15 mg/kg for groups S, P and C, respectively). After 504 days, no TC residues were present in the honey, except in one hive from group C (0.026 mg/kg). The half-life of TC in honey collected from hives was half that of honey stored in the laboratory under similar conditions (i.e., 35 °C in the dark), being 65 days versus 121 days. TC was quite stable in honey stored at 20 °C, with a half-life of 242 days. These results highlight the importance of the stipulations set out in EU regulations [154], which state that, in the case of bees, the veterinary surgeon must determine the appropriate withdrawal period by assessing the specific situation of a particular hive or hives on a case-by-case basis, in particular, the risk of residues in honey or other foodstuffs collected from hives and intended for human consumption.

It should be noted that, following administration to bees, antibiotics primarily accumulate in honey, but due to their physicochemical properties, they may also migrate into beeswax and persist there for extended periods. Chemical residues present in beeswax can subsequently contaminate honey in following beekeeping seasons. Consequently, improper beekeeping practices may lead to the accumulation of antibiotic residues in bees and in bee products as a result of migration from contaminated combs [171]. However, it should be noted that the presence of antibiotic residues in bee products may not always result from poor beekeeping practices. Highly interesting findings were reported by Savarino et al. [169] and Mosca et al. [171]. The authors linked the fact that honey bees, in their search for food and water, can forage at distances of up to 6 km from the hive with agricultural production activities. It was demonstrated that honey contaminated with antibiotics frequently originated from areas where livestock farming was practised. The identified substances are commonly used in veterinary medicine to treat infections in cattle, sheep, and pigs. Consequently, these drugs may be excreted by animals in their faeces or urine, enter the environment, be subsequently absorbed by plants from the soil, and be distributed into various organs and tissues, including flowers. It was also highlighted that certain antibiotics are administered to livestock through drinking water. If this water is accessible to bees, it may constitute an additional and significant source of contamination for bee products.

Antibiotics used in food-producing animals can affect consumer health due to their presence in food products in trace amounts, commonly referred to as residues. Some of these compounds can have direct toxic effects on consumers, while others can cause allergic or hypersensitivity reactions. For instance, β-lactam antibiotics (including antibiotics such as penicillin and cephalosporin) can induce skin eruptions, dermatitis, gastrointestinal symptoms, and even anaphylaxis at very low doses [173,174,175,176,177,178].

Oxytetracycline is a broad-spectrum antibiotic used to treat various infections, as well as a growth promoter for livestock. Symptoms of chronic exposure to oxytetracycline include a proinflammatory effect, haematological alterations, hepatotoxicity, and photosensitivity [171,179,180,181,182,183]. The compound may adversely affect calcium-rich organs such as teeth and bones; in children under seven, it can cause brown discolouration of the teeth [184,185]. While some toxicological studies suggest that this drug is not mutagenic, carcinogenic or teratogenic, others demonstrate that tetracycline antibiotics can have cytostatic, cytotoxic and mutagenic effects on cultured human blood lymphocytes [186,187]. A study by Li et al. [188] found that tetracycline use was associated with a modestly increased risk of basal cell carcinoma, but was not associated with melanoma or squamous cell carcinoma. As tetracycline antibiotics target bacterial translation, they can also affect eukaryotic mitochondria causing a mitonuclear protein imbalance and impaired mitochondrial respiration. Indeed, doxycycline treatment has been shown to disrupt mitochondrial proteostasis and metabolic activity, which can subsequently promote tumour formation [189]. Tetracyclines can also interact with nitrite(III) and nitrate(V) ions, or with ammonium, leading to the formation of N-nitrosamines, which possess strong carcinogenic properties [190,191,192]. On the other hand, it has been demonstrated that certain tetracycline antibiotics (doxycycline, minocycline, and tigecycline) possess anti-cancer properties by inhibiting proliferation, preventing metastasis, and promoting autophagy or apoptosis [188]. Therefore, the carcinogenic or anticarcinogenic action of tetracycline antibiotics depends on the type of cancer and the individual cell line [193,194]. Prolonged exposure to erythromycin has been associated with an increased risk of severe vomiting in infants, as a result of pyloric stenosis [183,195]. Moreover, erythromycin has been reported to have slight teratogenic effects. For example, congenital heart defects have been observed in infants born to women who took erythromycin during the early stages of pregnancy [196,197]. Enrofloxacin, an antibiotic belonging to the fluoroquinolone class, may contribute to increased embryonic mortality, induce cell death and DNA damage, as well as skeletal, reproductive, immune, neurological and digestive disorders [183,198,199,200,201]. Ampicillin, a derivative of penicillin and a β-lactam antibiotic, is widely used in cattle, pigs, poultry, and honey bees to control infection and as a feed or water additive to prevent certain diseases. Repeated contact with β-lactam residues has been shown to cause allergic reactions (ranging from mild symptoms to life-threatening anaphylactic shock), asthma attacks, exfoliative dermatitis, anaemia, thrombocytopenia, purpura, eosinophilia, leukopenia, and agranulocytosis [183,202,203,204,205]. Chloramphenicol is a bacteriostatic antimicrobial agent that was formerly used in veterinary medicine but is not currently authorised for use in the United States, Canada or the European Union (EU) [151,206]. The decision to ban the use of CHL in food production was influenced by results demonstrating its adverse health effects. It has been found to be potentially carcinogenic, rendering it unsuitable for use in food-producing animals, including honey bees. Research indicates that CHL exhibits both carcinogenic and genotoxic effects in humans, and its toxicity to the blood, bone marrow, kidneys, and liver has been confirmed. The most severe consequence of exposure to CHL is aplastic anaemia, a rare, unpredictable, dose-independent, and typically fatal idiosyncratic disorder [183,207,208,209,210]. Another compound, streptomycin, also exhibits significant adverse effects, such as impairment of vestibular mechanisms in the inner ear leading to a loss of balance (ototoxicity), neurotoxic effects in newborns, fever and skin rashes [211,212,213]. Among other antibiotics that are associated with major congenital malformations (MCMs) including organ-specific malformations, are clindamycin, doxycycline, quinolones, macrolides and phenoxymethylpenicillin [214].

Similarly, prolonged exposure to very low doses of certain antibiotics can result in adverse reproductive and teratogenic effects [215,216]. The excessive or uncontrolled use of antibiotics in apiculture can promote the emergence or amplification of antibiotic resistance within the honey bee microbiome and the surrounding environment. This resistance can likely be transferred to the human microbiome, thereby altering the qualitative and quantitative composition of the intestinal microbiota [170,178,217]. The intestinal microbiota play a vital role in human physiology and health [218]. Certain antibiotics have been shown to eliminate over 99% of beneficial gut bacteria, with recovery potentially taking several months. This can result in dysbiosis and gastrointestinal disturbances [219]. Excessive antibiotic exposure can lead to the emergence of bacterial strains that are resistant to antimicrobial agents and can persist in the human gastrointestinal tract for years. Furthermore, some epidemiological studies have demonstrated that chronic, low-dose exposure during critical developmental periods, such as the prenatal and infant stages, can disrupt the maturation of the microbiome, thereby influencing metabolic and microbiome programming and increasing the risk of becoming overweight or obese later in life [217,220]. Gut microbiota dysbiosis plays a significant role in the proliferation of opportunistic pathogens and the development of various diseases, including pseudomembranous colitis, colorectal cancer, inflammatory bowel disease, irritable bowel syndrome, inflammation, and obesity, as well as serious neurological and neurodegenerative conditions, such as autism, anorexia, depression, Alzheimer’s disease, and Parkinson’s disease [216,220,221,222,223,224]. A major issue that still requires extensive research is elucidating the toxic effects of antibiotic residue mixtures on both the human organism and the environment. While the toxicological consequences of individual antibiotics are relatively well characterised, the combined toxicity of antibiotic mixtures appears to be far more complex and difficult to predict.

In conclusion, the most common reason for the detection of antibiotic residues in bee products is their administration by beekeepers to bee colonies, primarily in countries with less stringent sanitary standards. Beekeepers use antibiotics to quickly treat American foulbrood, as well as to stimulate or protect the bees during intensive royal jelly production. This previously led to a temporary ban on imports of such honey and royal jelly from China to the EU and the US, which was lifted in 2004 [100]. Consumers should take this into account when deciding to purchase bee products from these countries.

### 2.5. Heavy Metals and Metalloids—Potentially Toxic Elements

Bee products, including honey, pollen, propolis, and beeswax, are valuable bioindicators used to monitor environmental pollution, particularly with regard to the accumulation of so-called heavy metals [98,225,226]. Heavy metals are chemical elements (metals and metalloids) characterised by high density (usually above 5 g/cm^3^) and are generally categorised into essential metals (such as Cu, Zn, Co, Mn, and Fe), non-essential metals (including Ba, Al, Li, and Zr), and toxic metals (such as Hg, Cd, Pb, Cr(VI), Sn, and the metalloid As) [227,228]. Exposure to heavy metals can result in a wide range of harmful and potentially lethal side effects, but their toxicity largely depends on their chemical form. On the other hand, essential metals are required, albeit in very low concentrations, for organisms to function properly, and deficiencies can cause serious diseases. However, exposure to higher concentrations of these elements, including Mn and Cr, may have a negative impact on health, which also depends on the route of exposure and the chemical form of the element.

Numerous studies have demonstrated that honey and other bee products may contain various toxic or potentially toxic elements, such as cadmium (Cd), copper (Cu), lead (Pb), zinc (Zn), nickel (Ni), manganese (Mn), mercury (Hg), chromium (Cr), cobalt (Co), iron (Fe), and arsenic (As) [27,149,229]. The accumulation of these metals in bee products is closely related to the degree of environmental contamination, particularly in terms of soil, surface water, and air quality, in areas where nectar-producing plants are present. The origin of heavy metals can be categorised as either natural or anthropogenic [230]. Natural sources include geochemical processes such as the weathering of parent rocks, soil erosion, volcanic activity, and the release of metals during paedogenic transformations. In contrast, anthropogenic sources encompass industrial activities (mining, metallurgy, and energy production), vehicular emissions, the uncontrolled discharge of municipal and industrial wastewater, and the use of mineral fertilisers and pesticides in agriculture, as well as urban dust emissions. It is widely acknowledged that human activity is the primary factor determining the presence and distribution of heavy metals in the environment. When collecting nectar, pollen, honeydew, balsamic substances, and water, honey bees (*Apis mellifera* L.) may simultaneously transport anthropogenic contaminants, including heavy metal ions, into the hive. These pollutants may subsequently infiltrate bee products. Additionally, trace amounts of toxic elements can appear in honey as a consequence of improper handling practices during processing, storage, or transportation. According to recent studies, elevated concentrations of heavy metals can be lethal to honey bees, and their presence in bee-derived products poses a potential health risk to consumers [108,229]. This is why the location of the apiaries is so important.

The permissible limits for heavy metals in foodstuffs are regulated by the relevant European Union legislation, including Commission Regulation (EU) 2021/1317 of 9 August 2021 amending Regulation (EC) No 1881/2006 regarding maximum levels of lead (Pb) [231] and Commission Regulation (EU) 2021/1323 of 10 August 2021 [232] regarding maximum levels of cadmium (Cd). It should be emphasised that, while the European Commission has established a safe limit for lead at 0.10 mg Pb/kg of fresh honey, these regulations do not specify a maximum allowable level of cadmium in honey. There are also no limits for the aforementioned metals with regard to other bee products.

The level of metals varies between different types of bee products. The mean concentrations (mg/kg) of heavy metals in honey, propolis, beeswax and pollen were as follows: honey: Fe (1.32) > Zn (1.31) > Pb (0.46) > Ni (0.18) > Cr (0.16) > Cu (0.14) > Co (0.12) > Mn (0.05) > Cd (0.03); propolis: Fe (8.0) > Zn (1.13) > Mn (0.59) > Pb (0.13) > Ni (0.07) > Cu (0.06) > Co (0.05) > Cr (0.03) > Cd (0.02); beeswax: Fe (1.31) > Pb (0.41) > Ni (0.407) > Zn (0.25) > Mn (0.12) > Co (0.10) > Cu (0.07) > Cr (0.05) > Cd (0.002), and bee pollen: Fe (2.2) > Zn (0.75) > Ni (0.25) > Pb (0.16) > Cu (0.05) > Mn (0.045) > Co (0.04) > Cr (0.01) > Cd (0.002) [225]. As can be seen, the Pb level in honey exceeds the safe value established by the European Commission for lead (0.10 mg Pb/kg), which may result in human poisoning. This is particularly important for children, as the CDC has stated that no safe blood lead level in children has been identified.

The concentration of heavy metals in bee products is influenced by various factors. For instance, the content of metals in bee pollen and monofloral honeys depends on the plant visited by the bees [226]. Bee pollen from *Salix* spp. was shown to contain higher amounts of Zn, Fe, Mn, Al, Cr, Cd, Ni than *Facelia* spp., *Taraxacum* spp. and *Malus* spp. bee pollens. In contrast, the highest Cu level was reported for *Facelia*, while the highest Pb level was found in *Taraxacum* pollen. Rapeseed honey contained the highest levels of Mn, Cu, Fe, and Mg, while the Zn level was the highest in linden honey. In another study [233], 23 monofloral honeys were tested, and the highest concentrations of heavy metals were reported in honey made from *Mentha* sp. (3.61 mg Fe/kg, 1.16 mg Zn/kg, 0.87 mg Ni/kg, 0.33 mg Pb/kg, and 0.036 mg Cr/kg), while honey produced from *Cyperus capitatus* had the highest level of Mn (0.67 mg/kg) and *Astragalus* sp. honey had the highest level of Cu (0.36 mg/kg).

The analysis of heavy metals can also be used to confirm the geographical origin of honey. No chromium, cobalt, cadmium, silver, bismuth, indium or tellurium were detected in the Turkish honey samples. Higher concentrations of Pb, Ni, Mg, Na, K and Mn were found in samples from the Burdur province, while the Cu level was statistically lower in Antalya than in other regions. No differences were observed in the levels of Al, Zn, Fe, Sr, Ba, Ca and Ga between regions [234]. In another study, the heavy metal content was analysed in composite honey samples obtained by blending five different multifloral honeys from specific regions of Pakistan: Faisalabad (FSD), Azad Kashmir (KSH), and Gujranwala (GRW) [235]. Although the cadmium level was below 0.01 mg/kg in all these samples, the concentrations of other metals were highly differentiated. The composite FSD honey contained the highest amounts (mg/kg) of: Fe (6.23 ± 0.04), Al (0.08 ± 0.32), Bi (0.11 ± 0.02), Mn (0.72 ± 0.06), Pd (0.22 ± 0.15), and Sr (2.72 ± 0.09). The KSH honey was characterised by the highest contents of Co (0.89 ± 0.03) and Zn (1.23 ± 0.06), while the GRW honey exhibited the highest level of Cr (0.56 ± 0.05).

According to the authors, the differences in the chemical composition of honey between regions could be attributed to geochemical soil composition and geographical differences. Significantly higher concentrations of Pb, Cd, and Fe were reported in honey samples from former mining areas compared to those from protected zones, suggesting pronounced industrial contamination [236]. The maximum recorded values were obtained for Pb (0.607 mg/kg), Cd (0.02 mg/kg), Fe (12.131 mg/kg), Cu (0.545 mg/kg), and Zn (6.170 mg/kg). Similar results were presented by Bayir and Aygun [237], who showed that the concentrations of Cr, Fe, and Zn in honey samples, and Cr, Cu, and Fe in bee pollen samples, were significantly lower in rural areas than in urban areas. The results obtained for honey and other bee products from apiaries situated in different locations in southern Poland (all within the region of the Małopolska Voivodeship) [209] confirm the correlation between the level of heavy metals in bee products and their presence in the air, water, and soil. This is connected with the combustion of coal and detritus, as well as dust fallout. Consequently, the concentration of heavy metals in bee products could serve as an effective monitoring tool for environmental contamination, as well as a means to verify the geographical origin of the products. It is therefore advantageous that a requirement has been introduced to indicate the geographic origin of honey in honey blends offered for sale [238]. This will enhance safety and traceability, while also making it easier to detect potential food fraud or adulteration.

As with other pollutants, heavy metals have the capacity to enter the bee body and be transferred by bees to the hive and bee products [210]. It seems reasonable to postulate that increasing environmental pollution caused by heavy metals may be one of the factors contributing to the decline in bee populations in recent years [239]. The results from other studies are in compliance with this hypothesis. The proximity of highways and industrial areas has affected the level and type of metal contamination in beeswax [240,241]. Honey collected from areas near a thermal power plant had significantly higher levels of Pb, Cd, and Zn compared to honey from an organic apiary. These metals originated from contaminated forage plants and the nectar collected from them by the bees [242]. Similarly, in honey collected by bees from plants growing near highways and industrial areas, the level of heavy metals correlated negatively with the distance from roads, with median concentrations of the heavy metals decreasing in the following order: Cu > Ni > Cr > Pb > Cd [241]. The samples of beeswax from the highway region (R1) contained the highest concentrations of metals, in the following order [240]: Ca > Zn > Fe > Cr > Cu. In wax samples from the urbanised area (R2), the order of metals was Ca > Fe > Cr > Zn > Cu, whereas in the industrialised region (R3), it was Ca > Fe > Zn > Cr > Cu > Mn. A significantly lower content of all the tested heavy metals was reported for the ecologically clean region (R4), with the order being: Fe > Zn > Mn > Cu > Cr. Cadmium was absent in all analysed samples (R1–R4), and nickel and lead were also not detected in R4 wax. The results obtained also showed that the concentrations of metals detected in beeswax were lower than those in honey bee bodies. This is consistent with the findings of other authors. In their study, Premkumar et al. [243] found that the zinc concentration in honey was significantly lower than in bees, with copper levels being 43 times lower and cadmium and lead levels nearly five times lower. This means that bees are particularly vulnerable when exposed to heavy metals.

Many scientific articles also indicate that other bee products, such as pollen, propolis, and wax, typically contain higher concentrations of heavy metals than honey [27,108,119,240]. First of all, this could be due to differences in the way of producing the bee products. Bee pollen and propolis tend to be the best indicators of environmental contamination as they are less processed by bees than wax and honey. Moreover, in the case of pollen, the higher level of pollutants is attributed to its direct contact with environmental elements, including polluted air and plant surfaces. In other cases, this phenomenon can be explained by the detoxification function of the honey bee’s body acting as a natural biofilter—mechanical and chemical contaminants are partially removed during nectar processing in the bee’s crop. However, due to the limited efficiency of the insect excretory system, a portion of toxic metals accumulates in the tissues of worker bees [239,244]. It has been demonstrated that *Lactobacillus* spp. and other lactic acid bacteria can accumulate lead, arsenic, cadmium, copper, and chromium [245,246,247,248], suggesting that the gut microbiome of honey bees plays a crucial role in reducing the burden of heavy metals within their bodies [249]. It is therefore in the best interests of beekeepers to identify suitable apiary locations. This will ensure the production of high-quality products that are uncontaminated by environmental pollutants.

Exposure to heavy metals can result in a wide range of harmful and potentially lethal side effects; however, their toxicity depends, to a large extent, on their chemical form. For example, Cr(III) is mildly toxic (IARC Group 3), whilst Cr(VI) is a highly toxic, carcinogenic substance (IARC Group 1). Acute lead toxicity is characterised by symptoms such as abdominal pain/colic, vomiting, constipation, peripheral neuropathy, cerebral oedema, and encephalopathy. These symptoms can lead to seizures, coma, and death [231]. Adverse effects of lead exposure occur at blood lead levels (PbB) below 50 ng/mL, with the most common health outcomes being neurological, renal, cardiovascular, haematological, immunological, reproductive and developmental effects. An increased risk of lung cancer was reported at PbB ≤ 100 ng/mL, whereas an increased risk of all cancers, respiratory tract cancer, stomach cancer, intestinal cancer, cancer of the larynx and glioma was observed at PbB > 100 ng/mL [228,250].

Fakhri et al. [251] reported interesting findings when they conducted a meta-analysis of heavy metal concentrations in honey samples from 28 countries. The authors found that levels of heavy metals in honey were relatively consistent regardless of geographic origin, with the highest mean concentrations observed for Fe, Mn, and Pb. The descending order of metal concentrations was as follows: Fe > Mn > Pb > Cr > Cu > Ni > Cd > As > Hg. A significant correlation was observed between increased Pb levels in honey and the presence of anthropogenic pollution sources around the hives. Health risk assessments indicated that heavy metal concentrations in honey were within safe limits (calculated hazard quotient (HQ) and total hazard quotient (THQ) were both lower than 1, and the carcinogenic risk (CR) was lower than 1 × 10^−6^). However, the authors highlighted that consumers in Turkey, Iran, and Bulgaria might be exposed to a higher health risk than those in other countries.

From a toxicological perspective, the heavy metals that pose the greatest risk to public health include lead, cadmium, arsenic, chromium, iron, cobalt, copper, zinc, nickel, and mercury [228]. It is important to note that some of these elements, such as Cu, Cr, Zn and Fe, perform essential biological functions and are required in trace amounts for proper metabolic activity. However, when their concentrations exceed physiological thresholds, they exhibit pronounced toxic effects. A good example is iron. Although it is a key micronutrient, the human body lacks an active mechanism for eliminating excess iron other than through bleeding. Therefore, consuming too much iron can be hazardous to health. Side effects include a strong pro-oxidative effect (the Haber–Weiss reaction, which leads to the production of reactive oxygen species and induces oxidative stress) and a type of oxidative cell death known as ferroptosis. This can result in the dysfunction of internal organs and systems, primarily the liver, heart, pancreas, kidneys, bones, immune system, nervous system and reproductive system. A dose exceeding 60 mg Fe per kilogram of body weight can be fatal for a child [252]. High concentrations of iron in food are particularly dangerous for people with undiagnosed hemochromatosis [253]. This means that monitoring iron levels in food, including honey, is very important. In light of the increasing fortification of food products and dietary supplements with iron, the European Food Safety Authority has recently updated its guidelines. The maximum safe daily intake of total iron is now set at 40 mg for adults, 10 mg for children aged 1–3 years, and 35 mg for adolescents (15–17 years). Due to their higher requirements, infants up to 11 months of age have a daily dose of 25 mg [254]. This means that monitoring iron levels in food, including honey, is crucial. The highest reported Fe levels in bee pollen (811 mg/kg) and perga 565 mg/kg) pose a significant health risk for consumers, with HQs of 32.29 and 11.51, respectively (Table A2 in Appendix A.2).

The most important health risks to humans resulting from the presence of heavy metals in food include [119,228,255,256,257,258,259,260,261,262,263,264,265,266,267,268,269,270,271,272,273]: (i) gastrointestinal disorders (nausea, vomiting, abdominal pain) (Zn, Cu, Fe, Pb); (ii) haematological and cardiovascular disorders (anaemia, hypertension, atherosclerosis, ischemia, ventricular arrhythmias, myocardial infarction, coronary artery disease) (Co, Cd, Pb, As, Hg, Cd, Ni); (iii) hepatic disorders (liver cirrhosis) (Zn, Cu, Co, As, Ni); (iv) neurological disorders (Alzheimer’s disease, Parkinson’s disease, Huntington’s disease, amyotrophic lateral sclerosis, multiple sclerosis), as well as cognitive or psychiatric impairments (Cu, Pb, Hg); (v) nephrotoxicity, abnormal kidney functioning or damage (Zn, Cr, Pb, As, Cd, Ni); (vi) immune system dysfunction (Zn, Hg, Ni); (vii) respiratory system dysfunction (Zn, Cr, Pb, Cd, Ni); (viii) endocrine disorders and reproductive and developmental disorders (Cu, Cr, Hg, Cd, Ni); (ix) allergic reactions (Zn, Pb, Ni); (x) generation of reactive oxygen species (ROS), increased mutagenicity and cancerogenic impact (various types of cancer, leukaemia) (Cu, Fe, Cr, Pb, As, Hg, Cd, Ni). Among other effects of heavy metals exposition are motor disorders, osteoporosis, diabetes, teratogenic effects, inhibition of collagen synthesis, coma.

The main mechanisms of heavy metal toxicity involve the generation of reactive oxygen species (ROS), inhibition of enzymatic activity, disruption of redox homeostasis, and impairment of endogenous antioxidant defence systems, in addition to causing genomic instability and impairment of DNA repair [274]. So-called Redox active metals include Co, Cr, Mn, Fe, and Cu. They damage cells by directly generating ROS, typically by up-regulating Haber–Weiss and Fenton reactions [255,275]. Non-redox active metals such as Cd, Ni, Hg, Zn and Al cause cell damage by indirectly increasing ROS levels through glutathione depletion, inhibition of antioxidant enzymes, binding of active thiol groups of proteins, and activation of ROS-producing enzymes such as NADPH oxidase [256,275]. Metal ions can also interfere with DNA and nuclear protein synthesis, leading to structural damage to genetic material, initiation of carcinogenesis, apoptosis, and modulation of the cell cycle [257,258,259,274]. Moreover, nickel is essential for the colonization and growth of several bacteria, fungi and protozoa, such as *Cryptococcus*, *Helicobacter*, *Staphylococcus*, *Clostridium*, *Vibrio*, *Mycobacterium*, *Yersinia*, *Escherichia*, *Proteus*, *Ureaplasma*, *Klebsiella*, *Pseudomonas*, *Corynebacterium*, *Trypanosoma cruzi*, *Leishmania* and many more, that infect the human body and cause various diseases [276].

Although there are no regulations regarding the maximum permissible concentrations of metals in honey (except for Pb) and bee products, an attempt was made to assess whether these elements pose a risk to consumer health by taking into account the RfDs provided by various agencies and the measured concentrations. As shown in Table A2 (Appendix A.2), lead may pose a health risk to consumers, as the HQ is 3.36829 for bee pollen and 1.65009 for perga. A hazard quotient above 1 was also calculated for cadmium (Cd) in perga (3.18759) and arsenic (As) in bee pollen (12.63), suggesting a potential health risk, especially for consumers undergoing apitherapy. The levels of other metals in honey and bee products do not seem to pose a health risk.

### 2.6. Mycotoxins

Another source of chemical contamination associated with bee products arises from the possible presence of moulds. It is worth mentioning from the very beginning that honey is an environment that is exceptionally unfavourable for the growth of moulds, primarily due to its low water activity (high osmotic pressure caused by the presence of sugars). Consequently, the calculated HQ value for honey is always below 1. Honey’s density also does not favour mycelium growth nor the production of mycotoxins. If mycotoxins are present in honey, they are usually found in trace amounts resulting from cross-contamination from honeycomb cells. Among bee products, propolis and royal jelly are generally considered in the context of inhibiting mould growth and preventing the synthesis of mycotoxins by moulds rather than being considered as their source. Propolis extracts contain phenolic compounds and flavonoids that can block the expression of genes responsible for the biosynthesis of aflatoxins in *Aspergillus fungi* [277,278]. The situation is entirely different when it comes to bee pollen and perga. As these products contain a high proportion of protein and fat and readily absorb moisture from the environment, mould fungi can easily colonise them. Many of these microorganisms (e.g., *Aspergillus*, *Penicillium*, *Fusarium*, and *Byssochlamys*) are capable of synthesising a wide range of mycotoxins, including aflatoxin B_1_ (AFB_1_), aflatoxin B_2_ (AFB_2_), ochratoxin A (OTA), patulin (PAT), zearalenone (ZEN), deoxynivalenol (DON), penicillic acid (PA), sterigmatocystin (STE), gliotoxin, and fusarenon X (FX), which are well known for their toxic effects and their adverse impact on human and animal health [279,280]. The optimum conditions for mycotoxin production are a_w_ > 0.98 and temperatures ranging from 25 to 35 °C, whereas the optimum pH depends on the species and strain of the mould and usually oscillates between 4.0–5.0. This means that, after harvesting, these products must be dried quickly and thoroughly and must also be stored at the correct temperature in airtight packaging.

According to the literature, the mycotoxins most frequently identified in bee products are AFB_1_, DON, ZEN, fumonisins, OTA, and T-2 toxin [281,282,283,284,285]. Of the known mycotoxins, aflatoxin B_1_ is considered the most hazardous to human and animal health. It is recognized as one of the most potent naturally occurring carcinogens (IARC Group 1), for which no exposure level is regarded as safe. Studies conducted by Carrera et al. [281] have shown that bee pollen and bee bread pose a particularly high risk of mycotoxin contamination among bee products. The authors reported the presence of mycotoxins in all fresh and dry bee pollen samples tested, which were collected from 28 countries involved in the study, including Poland, Italy, Spain, the United States, China, Russia, and India. The highest determined concentration of DON was 135 μg/kg, with 28% of the samples exceeding the permissible safety limit. The highest levels of other mycotoxins were 622 μg ZEN/kg, 5.3 μg AFB_1_/kg, and 7.8 μg OTA/kg. T-2 toxin was not detected in the analysed samples.

Regarding the carcinogenic potential of aflatoxins and ochratoxin A (IARC Group 2B), no level of exposure is considered safe, so no RfDs have been established. The Margin of Exposure (MoE) is therefore usually calculated to assess exposure to these toxins, defined as the ratio of the benchmark dose lower confidence limit 10% (BMDL_10_) to the level of exposure (EDI). According to the EFSA, the risk is of low concern for public health when the MoE ≥ 10,000, while MoE < 10,000 constitutes a high public health concern. In mentioned studies [281], the MoE values calculated for AFB_1_ in dry bee pollen often yielded values below 10,000. The lowest values were recorded in pollen from Hungary, with MoE values of just 209 and 238 for boys and girls, respectively. Similarly low values were found for pollen samples from France (MoE = 238 for elderly women), Cyprus (MoE = 278 for female adolescents), and Slovenia (MoE = 263 for female toddlers). Regarding OTA, values below 10,000 were observed for the aforementioned pollen samples, ranging from 7242 to 9656. Slightly better results were obtained for fresh pollen, but this still means that consuming bee pollen can pose a risk to consumer health. Although OTA was not detected in perga, the reported AFB_1_ levels indicated a health risk, with 84.6% of samples having an MoE of less than 10,000 (ranging from 181 to 6528).

Monofloral pollen was found to exhibit lower mycotoxin concentrations compared to multifloral pollen [281,282]. The obtained results are of considerable importance in terms of food safety and consumer health, particularly given the growing popularity of bee pollen consumption. Although the presence of the aforementioned mould species has also been reported in honey, it should be emphasised that the detection of fungi in food does not necessarily indicate the presence of mycotoxins, just as the removal of fungal cells does not guarantee the elimination of these compounds due to their high physicochemical stability.

Aflatoxins have been detected in bee pollen samples collected from various geographical regions, including Serbia, Turkey, and China, with AFB_1_ levels reaching up to 17.32 μg/kg [15,286,287,288,289]. However, many papers report that aflatoxin levels in bee pollen are below the detection limit of the methods used (e.g., ELISA, HPLC with fluorescent detection, LC-MS/MS) [290,291,292,293,294].

AFB_1_ exhibits the ability to induce genetic material damage, including induction of DNA mutations, chromosomal aberrations, and degenerative changes within the liver [295,296]. After ingestion, AFB_1_ is metabolised by cytochrome P450 enzymes in the liver, resulting in the formation of highly reactive metabolites [297]. The most dangerous of these is aflatoxin B_1_-8,9-epoxide, which covalently binds to DNA. This leads to mutations in the TP53 tumour suppressor gene, resulting in the loss of function of the key p53 protein, which is responsible for maintaining genome integrity, facilitating DNA repair, and inducing apoptosis in response to cellular damage [283,298]. Aflatoxins also interfere with numerous cellular processes, including post-translational protein modifications, as well as the methylation of proteins and nucleic acids. They affect mitochondrial DNA function and cellular respiration pathways, contributing to increased production of reactive oxygen species [299]. The most severe tissue damage occurs in the liver, kidneys, and central nervous system. The clinical manifestations depend on the dose and frequency of toxin exposure. Two forms of poisoning are distinguished: acute intoxication caused by high doses of aflatoxins and chronic intoxication resulting from prolonged exposure to low concentrations [299,300]. The latter model suggests that the long-term consumption of bee products containing even trace amounts of aflatoxin may have negative health consequences. Symptoms of acute poisoning include pulmonary oedema, internal haemorrhages, severe abdominal pain, nausea, vomiting, jaundice, convulsions, and coma. Chronic exposure, on the other hand, leads to the development of hepatic cirrhosis, allergic reactions of the skin and respiratory tract, growth and developmental impairments, cognitive dysfunction, and oedema of the lower limbs [283,301,302]. Chronic exposure to aflatoxins also induces immunosuppression by impairing immune system function. This reduces the number and activity of immunocompetent cells, such as T and B lymphocytes and macrophages, thereby hindering the elimination of neoplastic and preneoplastic cells [303]. A weakened immune response increases susceptibility to viral infections, including HIV infection [304]. Moreover, a significant correlation has been observed between aflatoxin exposure (including during the prenatal period) and growth retardation in infants, suggesting the potential impact of these toxins on physiological development during early childhood or even in the prenatal period [297]. This is another reason why honey and bee products should be given to children with great caution, if at all. Children are more susceptible to the effects of aflatoxins. The numerous potential causes of the observed growth retardation and adverse health effects associated with exposure to aflatoxin include the following [297,302,303,304,305,306]: nutritional deficiencies resulting from the increased intestine permeability, as well as impaired absorption and metabolism of essential macro- and micronutrients required for normal growth and development; chronic inflammation, which may lead to metabolic disturbances and a reduced ability of the body to utilise nutrients efficiently; impaired immune system function, leading to increased susceptibility to infections, often associated with elevated metabolic demands and a simultaneous decrease in nutrient absorption capacity, which may ultimately contribute to growth inhibition; hormonal dysregulation involving disturbances in the hypothalamic–pituitary–adrenal axis, suppressing the release of IGF-1 from the liver and other endocrine systems that play a key role in growth and maturation processes; and prenatal and lactational exposure, as mothers exposed to aflatoxins may transmit these toxins to their offspring both transplacentally and through breast milk.

Long-term exposure to aflatoxins has also been linked to the development of neurodegenerative and dementia-related disorders. It is believed that these toxins may adversely affect the integrity of the blood–brain barrier by disrupting the phospholipid structure of cellular membranes, thereby increasing barrier permeability and enhancing the susceptibility of neural tissue to neurotoxic agents. Aflatoxins also form stable complexes with melanin, preventing its proper utilisation in the body. As a consequence, depigmentation of the skin and dysfunction of the substantia nigra in the central nervous system may occur, potentially contributing to the development of pathological changes characteristic of Parkinson’s disease. Moreover, aflatoxins may induce and exacerbate allergic reactions, including rhinitis, allergic skin lesions, bronchial asthma, and hypersensitivity pneumonitis. Exposure to these toxins leads to an enhanced immune response to airborne allergens, particularly to the spores and conidia of filamentous fungi [304].

Ochratoxin A exhibits a broad spectrum of toxic effects, with nephrotoxicity being the predominant manifestation [307,308]. OTA is considered one of the major etiological factors responsible for numerous acute and chronic renal disorders, which are characterised by degenerative alterations in proximal tubular epithelial cells and progressive interstitial fibrosis. Damage to tubular structures leads to impaired renal excretory function, manifested by increased urine output (polyuria); the presence of glucose (glucosuria) and proteins (proteinuria) in the urine; and elevated activity of enzymes such as γ-glutamyl transferase, alkaline phosphatase, and leucine aminopeptidase (enzymuria), which serve as markers of tubular cell injury [309,310,311,312,313]. OTA undergoes biotransformation in the liver via cytochrome P450 enzymes, leading to the formation of reactive metabolites that interfere with hepatocellular metabolic pathways. These processes result in the increased production of reactive oxygen species (ROS), DNA damage, and the activation of apoptotic cascades, which may ultimately contribute to the development of chronic liver diseases [314,315,316]. Furthermore, several studies have indicated the teratogenic potential of OTA. Experiments conducted on rat models have demonstrated increased prenatal mortality, reduced neonatal body weight, and developmental malformations of the skeletal system, visual organs, and craniofacial structures [296,317,318,319,320].

T-2 toxin (364.7 μg/kg and 265.4 μg/kg) and ZEN (361.6 μg/kg) were detected in sunflower, rape, and poppy pollen from Slovakia, respectively [321], while DON and ZEN (120 and 280 μg/kg, respectively) were detected in bee pollen from the Brassicaceae family in Lithuania [285]. Zearalenone is a non-steroidal mycotoxin with oestrogenic activity, produced primarily by fungi of the genus *Fusarium*. This compound is capable of interacting with the endocrine system through binding to 17β-oestradiol receptors, causing a hormonal imbalance manifested as hyperestrogenism and reproductive dysfunctions. Consumption of food contaminated with ZEN poses a significant health risk due to its structural similarity to endogenous oestrogens. Prolonged exposure to ZEN may result in excessive stimulation of oestrogen receptors and consequently promote the development of hormone-dependent cancers, such as breast, ovarian, and endometrial carcinomas [296].

Trichothecenes constitute a group of cyclic sesquiterpenes containing an epoxide ring, produced by fungi belonging to various genera, such as *Fusarium*, *Trichoderma*, *Stachybotrys*, and *Trichothecium* [322,323]. The mechanism of trichothecene toxicity is primarily based on the inhibition of protein biosynthesis through interaction with the peptidyl transferase centre of the 60 S ribosomal subunit in eukaryotic cells. Additionally, these toxins induce oxidative cellular damage, leading to disturbances in nucleic acid synthesis, the destabilisation of cellular structures, and the activation of apoptotic pathways [300]. Trichothecenes readily penetrate the mucous membranes of the gastrointestinal tract, resulting in rapid absorption and wide distribution throughout the body, affecting multiple tissues and organs. Exposure to these toxins is associated with gastrointestinal dysfunction and the occurrence of anorexia. This mechanism may be direct or indirect, affecting hypothalamic centres responsible for appetite regulation and the secretion of anorexigenic factors. Studies have shown that trichothecenes can modulate the activity of enteroendocrine cells, leading to the increased secretion of appetite-regulating hormones such as cholecystokinin (CCK), peptide YY (PYY), glucagon-like peptide-1 (GLP-1), and 5-hydroxytryptamine (serotonin). These compounds contribute to the sensation of satiety and the suppression of appetite [324,325]. A particularly hazardous member of this group is the T-2 toxin, which is characterised by its high lipophilicity and systemic toxicity, resulting from its ability to easily cross biological barriers. The T-2 toxin exhibits hepatotoxic effects by inducing hepatocyte apoptosis, as well as nephrotoxic, cytotoxic, and immunosuppressive activities [326]. Exposure to high doses of the T-2 toxin may lead to the development of alimentary toxic aleukia (ATA), manifested by neurological symptoms (dizziness, loss of balance, headaches, apathy, seizures), haematological disorders (bleeding from the nose, gums, skin, and mucous membranes), damage to the bone marrow and thymus, necrosis of mucosal tissues, and gastrointestinal dysfunction (nausea, vomiting, diarrhoea, abdominal pain). In severe cases, T-2 toxin poisoning may result in shock and death [300,324,326,327,328,329,330].

Exposure to mycotoxins has also been shown to lead to the dysregulation of the intestinal microbiota ecosystem, the impairment of mucosal barrier function, and the disruption of local immunoregulation, which, at the systemic level, may result in the development of systemic toxicity and the progression of chronic mycotoxicosis [325].

To conclude, the official legal acts that govern permissible levels of mycotoxins in food within the European Union [148] rarely mention bee products. In only a few instances are honey (for lead) or food supplements containing bee pollen or propolis (for polycyclic aromatic hydrocarbons and pyrrolizidine alkaloids) specifically listed. The limits set out in these regulations for aflatoxin B_1_ range from 0.1 to 12 µg/kg depending on the food matrix, for total aflatoxins from 4 to 15 µg/kg, for OTA from 0.5 to 20 µg/kg, for patulin from 10 to 50 µg/kg, for DON from 200 to 1750 µg/kg, for ZEN from 20 to 400 µg/kg and for fumonisins B_1_ + B_2_ from 200 to 4000 µg/kg. When comparing results from various publications, it is clear that honey poses virtually no health risk (HQ < 1). Furthermore, analysis of individual mycotoxins in other bee products also usually shows an HQ < 1. However, pollen and bee bread may be heavily contaminated with multiple mycotoxins. In these cases, the concentrations of individual mycotoxins detected may exceed permitted levels established for other food products intended for direct consumption. Furthermore, cumulative hazard indices (HI), particularly for DON + ZEN, may exceed 1 for certain consumer groups, such as children and the elderly. It is also crucial to monitor the concentrations of the two most dangerous mycotoxins, AFB_1_ and OTA, as these have proven and potential carcinogenic effects. Even trace amounts of these compounds in bee products may pose a high health risk to individuals undergoing apitherapy, as expressed by an MoE below 10,000.

## 3. Physical Contaminations of Bee Products

### Microplastics

Microplastics surround us everywhere and in every situation. They have been found in every environment and corner of the world. They are present in food; various types of water, including natural reservoirs, tap water and bottled water; the air we breathe; the soil; and plant raw materials and come both from industrial pollution and private households [331,332,333,334,335,336,337,338,339]. Microplastics are classified as physical contaminants, in the same way as pieces of glass or metal fragments that get into food. It should be emphasised, however, that the smallest plastic particles can cause effects similar to those of chemical pollutants. As microplastics break down, they may release degradation products, chemical additives contained within the plastics, or other environmental pollutants they have adsorbed.

The fact that there is no single type of ‘microplastic’ makes it challenging to detect them and determine how they affect the human body and health. Microplastics exist in a wide range of sizes, shapes, and chemical compositions, each of which may influence cells and tissues differently. Several forms of microplastics can be distinguished based on size. Typically, microplastics are categorised as nanoplastic when the plastic particles measure ≤1 µm or as microplastic when they measure less than 5 mm. However, other classifications are also used [340,341,342]. Based on shape, fragments, beads, fibres, spheroids, granules, pellets, foam (polystyrene), and flakes can be recognised. Direct sources of microplastics include items made from polyvinyl chloride (PVC, accounting for ~20% of the total), polystyrene (PS, ~20%), polycarbonate (PC, ~15%), polyethylene (PE, ~12%), polyethylene terephthalate (PET, ~10%), polypropylene (PP, ~8%), polyamide (PA, ~6%), and polyethersulfone (PES, ~4%), as well as polyurethane (PU/PUR), epoxy resins, silicones, polyester, and many others [334,340,343].

As microplastics are present in the air, soil, and water, they can easily enter plant and animal tissues (including bees), thereby entering the food chain and the human body [332,344,345,346,347,348].

Various studies have suggested that microplastics primarily enter the human body via digestion but also through inhalation or dermal penetration [334,349]. Depending on their size, such particles can then accumulate in various tissues and organs in the human body. Current data suggest that the average person may ingest between 39,000 and 52,000 microplastic particles per year [333]. Microplastics in the gastrointestinal tract are likely to be absorbed by immune cells, with nanoplastics being absorbed more easily due to their smaller size. Furthermore, nanoplastic particles may interact with biomolecules to form a protein corona, which may influence absorption and be altered during digestion [349]. Microplastics can also enter the body through polluted air, albeit to a lesser extent. During respiration, approximately 26–130 airborne microplastic particles may enter the body [350]. Particles that are inhaled into the lungs and measure between 0.1 and 10 µm are particularly harmful because they can easily cross the alveolar barrier and enter the circulatory or lymphatic systems. Once in the bloodstream, these particles can circulate throughout the body and migrate to other organs; they can even cross the blood-brain or placental barriers [349,351,352].

While this is a less significant route of entry into the human body, it still poses a potential threat to bees, which may ingest microplastics in this way in environments contaminated with micro- and nanoplastics. Nanoplastic particles smaller than 100 nm from our clothing, water, personal care products or cosmetics can also penetrate human skin, especially damaged skin [349]. The absorption of microplastics through the skin generally occurs through damaged tissue. Therefore, it should be kept in mind that cosmetics or medicines designed to alleviate skin problems and made from honey, propolis or bee bread may also introduce microplastics into the body [353]. Microplastics present at sites of skin injury may cause local toxicity, mechanical damage, or disrupt the integrity of the skin barrier [349,354]. Once introduced, microplastics can accumulate in various organs of the human body, including the liver, colon, lungs, placenta, skin, and hair, as well as in faeces and physiological fluids such as blood, saliva, sputum, breast milk, and bronchoalveolar lavage fluid [349]. Micro- and nanoplastics have also been detected in atherosclerotic plaques in the carotid artery, suggesting a potential role in inflammatory responses and an increased risk of cardiovascular disease [355]. Concentrations of microplastics in the brain tissue of individuals with neurodegenerative diseases were 7–30 times higher than those found, for example, in the liver or kidneys [356]. Microplastics have also been found in the lymphatic and urinary systems, as well as in meconium, sputum, and urine [357].

Besides the fact that microplastics have a negative impact in their own right, they can also carry toxic chemicals added during their production, such as chlorinated paraffins, phthalates, phenols and bisphenols. These compounds negatively impact honey bees [358] and can also contaminate honey and other bee products [359,360].

The most significant sources of microplastics in the human body are water, various beverages, food (particularly seafood, such as oysters, shrimp, crabs, and fish), plant raw materials and some herbivorous animals or their products (honey being one of examples). Other sources include food additives such as sugar (including sugar administered to bees in apiaries) and salt are other sources [361,362,363,364,365,366,367,368,369].

For the past 20 years or so, microplastics have increasingly been recognised as a source of pollution in both aquatic and terrestrial ecosystems and among the organisms living in them, including pollinators. Shavali Gilani et al. [22] reviewed the effects of microplastics on honeybee health and the presence of microplastics in honey. Microplastics can affect honeybee health in several ways, by including increasing mortality and influencing sucrose responsiveness and habituation, olfactory learning, colony performance, and body size and growth. Microplastics may alter the composition of bees’ gut microbiota, thereby negatively affecting their immune system and overall health and weakening bees’ resistance to viral infections [370,371,372]. Disrupting the balance of the gut microbiome adversely affects the immune system and impairs the essential neurological signalling pathways for learning and memory processes in bees [372,373]. There is a high likelihood that microplastics can change the bees’ behaviour, impair bees’ ability to forage effectively, leading to mass colony collapse and disrupting ecosystem functioning [373,374]. Of particular concern is the fact that bees may use plastic waste, such as pieces of polyethylene film and polyurethane construction sealant residues, to build and seal brood cells in their nests instead of natural materials like plant leaves or tree resins [375]. Consequently, microplastic particles may subsequently find their way into the products produced by bees, including honey and royal jelly. Although research on the impact of microplastics on honey bees and bee products is still in its early stages [376], it seems probable that microplastics from the environment can accumulate in the tissues of honey bees and other pollinators, affecting their health and, thereby, the quality of their products [375,377,378]. It is also predicted that microplastics will have a negative impact on other pollinating insects, including bumblebees [379].

Table 2 summarises selected studies on the presence of microplastics in honey bee organisms and bee products. The results showing the presence of MNPs in bee bodies provide clear evidence of the impact of environmental pollution, as well as demonstrating the potential of these studies for environmental biomonitoring. In turn, the detection of MNPs in food products highlights the need for further research to assess the actual risk posed to consumers by the presence of these particles in honey and bee products, particularly given their presence in other food products and drinking water. Understanding the key pathways through which bees, honey and other bee products become contaminated by microplastics enables beekeepers to implement sustainable hive management practices that minimise exposure to microplastics. Moreover, legal regulations are required to control microplastic contamination, ensure the safety of honey, protect pollinators, and safeguard human health [22].

**Table 2:** Studies on the presence of micro- and nanoplastics (MNPs) in bee organisms and bee products and the impact of these particles on bee health and behaviour.

Tested Sample	MNP Characteristics	Methods for Detecting	Additional Information	Results (Concentration and/or MNP Impact)	References
Visual methods assessing the MNP concentration, colour and size					
Honey bees (*Apis* *mellifera*)	Spherical polystyrene particles with a diameter of 25 μm (PS-MP), used alone or in combination with tetracycline (PS-MP)	Confocal microscopy, SEM	Simulation of microplastic contamination in bees under laboratory conditions; microplastics as a carrier for antibiotics.	Reduction in the α-diversity of the bees’ gut microbiota; changes in the structure of microorganisms; changes in the expression of antioxidant genes (Cat); detoxification genes (CypQ1 and GstS3) and genes associated with the immune system (Domeless, Hopscotch and Symplekin) in the gut; accumulation and degradation of PS-MP in the large intestine, and interaction with gut bacteria.	[380]
Honey bees (*Apis* *mellifera*), honey, bee pollen	MNPs varied in size, shape, colour, polymer composition; the samples contained fibres of various colours (mainly blue) and their fragments	Stereomicroscopy, with or without preliminary digestion with H_2_O_2_	The studies were carried out in rural and urban areas in 2021 and 2023.	The most common type of microplastic found in bees, pollen, and honey was blue fibres, the quantity of which was higher in 2023 than in 2021.	[381]
Honey, wax, larvae, and honey bees (*Apis* *mellifera*)	Blue polyester microfibres	Dino-Lite AM3111 digital microscope (magnification up to 250×)	The bees were fed sucrose solutions with or without the addition of 50 mg of polyester microfibres per litre for one month.	The microfibres were ingested by adult worker bees and were present in the cuticle, the digestive tract, the larvae, honey and wax (the majority of the microplastics).	[382]
Stingless bee products (*Heterotrigona itama*)—honey, pollen, and propolis	Fibres (80.4%) > fragments > filaments and foam. Fibres and filaments size ranged 25.8–120.5 μm, max. diameter of fragments measuring 154.1 μm, max. diameter of foam 78.4 μm.	Stereoscopic microscopy	The samples came from six meliponiculture sites (urban, semiurban and rural/garden locations) in the Meratus Geopark in South Kalimantan, Indonesia.	Rural-yard honey (Bumdes Ozi Kelulut) had the greatest contamination level at 60 particles/100 mL, followed by urban-yard pollen (Budidaya Kelulut Kalimantan) at 37 particles/100 mL and rural-yard propolis (Kelulut Dua Puteri) at 49 particles/100 mL. Apart from urbanization, other factors contribute to microplastic pollution (agricultural practices and atmospheric deposition).	[383]
Honey	Fibres and fragments	Filtration with 30% H_2_O_2_ digestion; dissecting microscope at	19 honey samples, mainly European (Germany, France, Italy, Spain) and from Mexico. Honeys were both in solid and liquid form, monofloral or multifloral or not specified.	40 to 660 fibres/kg of honey (mean value of 166 ± 147/kg of honey), (0 to 38 fragments/kg of honey (mean 9 ± 9/kg of honey). Honeys from Germany, Spain and Italy had highest fibre numbers. Microplastic particles were transported by the bees into the hive, or have been introduced during honey processing (e.g., from plastic bags used by beekeepers to sugar) or both.	[368]
Honey	Fibres were more abundant and bigger than fragments. Black carbon particles were also encountered.	Filtration with 30% H_2_O_2_ digestion; dissecting microscope	47 honey samples from supermarkets or directly from German small-scale beekeepers.	10 to 336 fibres/kg and 2 to 82 fragments/kg were found in all tested honey samples.	[384]
Analytical methods identifying the chemical composition of MNPs					
Honey bees (*Apis* *mellifera*), honey	MNPs smaller than 5 mm; synthetic microfibres and natural/artificial fibre fragments with a diameter of less than 50 μm	FTIR	The study was carried out in apiaries located in areas with high and low levels of urbanisation in southern Italy.	Polytetrafluoroethylene (PTFE) (from soil and air) was detected in the bees’ bodies, whilst polyethylene-based microplastics (from agricultural usage) and polycaprolactone (PCL) (from biodegradable materials) were found in the honey.	[385]
Honey	4 polymers: EVA, PE, PP and nylon-6, fibres and fragments, 6 different colours (black, brown, green, red, yellow and transparent)	Stereomicroscope and FTIR	Honey was produced in Turkey in 2023.	The average microplastic concentration was 314 ± 353 particles/kg in the 32 honey samples analysed (ranging from 0 to 1280 particles/kg). ADI and HI for monofloral and multifloral honey were, respectively: 438 and 310 particles/year (ADI) and 16.7 and 6.70 (HI).	[363]
Honey	MNPs size from 20 to 541 µm; 2 different shapes (fibres and fragments); 7 different colours (black, blue, transparent, green, red, brown and purple); 5 different polymer structures (EVA, PE, nylon-6, PET and PP)	Stereomicroscope and attenuated total reflection/FTIR	28 samples of honey produced in Kosovo (Serbia) in 2024 were analysed.	Average levels of the microplastic diversity index (90.31 ± 0.25), microplastic load index (2.72) and polymer risk index (75 ± 62) was assessed. Microplastic intake via honey was calculated at 136 ± 75 particles/year.	[386]
Honey bee (*Apis* *mellifera*), bee pollen	5 different synthetic polymers; polyethylene terephthalate (PET) dominated in fibres, while in nonfibrous microparticles, polypropylene (PP), polyethylene (PE), polyacrylonitrile (PAN) and Polyamide (PA) dominated. Fibres > fragments and films. Main colours: black, blue, green and red	Novel in-hive passive sampler named the APITrap Stereoscopic microscopy, FTIR	Five different apiaries in Denmark.	Microfibers and non-fibrous particles detected in bees and in pollen, as well as in APITrap. Microplastic average number ranged from 39 to 67 particles in the APITrap; in 5-g honeybee samples, 6 to 9 particles were detected, while in 5-g pollen samples, 6 to 11 particles were detected. Microplastic in honey bees had average sizes of 387 μm (fibres), 72 μm (fragments) and 363 μm (films). Pollen particles average size were, respectively, 435 μm, 64 μm and 332 μm.	[387]
Honey bees (*Apis* *mellifera*)	Fragments (52% of total MNPs) and fibres (38%) with average equivalent diameter of 234 ± 156 µm and 64 ± 39 µm, respectively; 13 synthetic polymers (PES > PE> PVC > PU)	micro-FTIR	Honey bees from apiaries located in the centre of Copenhagen and from nearby suburban and rural areas were studied.	The highest load (5.5 particles/100 bees) corresponded to urban apiaries, while hives from suburban and rural areas had comparable amount of MNPs (1.0–1.5 particles/100 bees). Worker bees can act as active microplastic samplers.	[388]
Honey bees (*Apis* *mellifera*)	Feeding bees with PS (4.8–5.8 μm) and PMMA (1–40 μm) and their combinations (MIX)	Gas chromatography coupled to mass spectrometry (GC-MS)	Microplastic doses: 0.5, 5 and 50 mg/L, their impact on the immune system and survival of worker bees collected from apiary in Florence (Italy).	Negative effects of PS and PMMA on bee survival and immune response. Even at ecological concentrations, changes in cuticular profiles were observed, but PMMA was the main cause; both microplastics caused a reduction in the abundance of several cuticular compounds	[389]
Honey	Fibres amount ranged from 20 to 178 particles/L of honey, while fragments from 126 to 828 particles/L. The microplastic size of fibres (67.18–5174.01 µm) was bigger than that of fragments (5.63–226.01 µm). Polypropylene, low- and high-density polyethylene and polyacrylamide were detected.	Microfiltration followed by degradation of organic matter with H_2_O_2_; FTIR	14 honey samples packaged by hand and packaged industrially in glass container were collected. They came from urban and rural localisations in Ecuador.	Average value of microplastics found in industrial and craft honey were 54 MPs/L and 67 MPs/L, respectively. Honey processed in highly populated cities presented a greater quantity of microplastic particles greater than 100.	[390]

FTIR—Fourier-transform infrared spectroscopy, EP—epoxy resin, EVA—ethylene-vinyl acetate, PA—polyamide, PAN—polyacrylonitrile, PE—polyethylene, PET—poly(ethylene terephthalate), PMMA—poly(methyl methacrylate), PES—polyester, PP—polypropylene, PS—polystyrene, PU—polyurethane, PVC—poly(vinyl chloride).

Awareness that bee products may be a source of microplastics in the human diet is very important. Consumption of honey and comestibles containing honey results in the ingestion of microplastics, which can exert a deleterious effect on both the physical and chemical composition of the human body. Microplastics present in tissues and cells can induce oxidative stress, trigger inflammation, damage to biological membranes, stimulate genotoxicity and apoptosis leading to disruption of cellular homeostasis, cytotoxic effects, and impairment of cellular functions, potentially contributing to immunological and hormonal dysregulation [349,391,392,393,394]. Sutkar et al. [343] analysed the mechanisms through which microplastic particles can induce oxidative stress, chronic inflammation, and cellular toxicity in multiple systems and organs in the human body, indicating apoptosis, necroptosis, ferroptosis and pyroptosis as the most probable routes. Microplastic toxicity on gastrointestinal tract includes increased pro-inflammatory response, oxidative stress, apoptosis, intestinal barrier dysfunction, broken homeostasis and dysbiosis of gut microbiota. Microplastic particles may also lead to endothelial dysfunction and instability of atherosclerotic plaques, platelet activation, and elevation of blood pressure, thereby increasing the risk of cardiovascular disease development [355,395,396,397,398]. The neurotoxic effects of microplastics (e.g., enhanced risk of cognitive impairment, Alzheimer disease, Parkinson disease, depression episodes) result from their penetration through the blood–brain barrier and disruptions in neurotransmitter function [399,400,401,402,403,404]. Microplastics may also contribute to genomic instability and activate signalling pathways and mechanisms potentially responsible for cellular transformation in the human body; therefore, it is thought that they can promote carcinogenesis [340,405]. Dzierzyński et al. [340] have summarised studies investigating both direct and indirect effects of micro- and nanoplastics on carcinogenesis. Most of these studies were conducted on human cell lines in vitro or on animal models in vivo. Zhao et al. [406], detected microplastics in various forms in human cancers, including the stomach, colon, lung, cervix, and pancreas. Exposure to microplastics has been associated with cancers of the pancreas, liver, biliary tract, colon, prostate and endocrine system [407,408,409]. Microplastics may activate the ROS–NF-κB/NLRP3/IL-1β/MLCK stress signalling pathway, inducing oxidative stress and the increased production of free radicals. This can potentially contribute to inflammation and lead to intestinal barrier damage, gut microbiota dysbiosis and microbial metabolism disruption [410]. Moreover, microplastics can exert effects at the cellular level, exhibiting toxicity towards lysosomes, mitochondria, the endoplasmic reticulum, and the cell nucleus [411]. Although there are no studies directly investigating the effects of microplastics on the human reproductive system, it is known that they can accumulate in the reproductive tissues of various animals (e.g., the semen, testes, uterine tubes, and placenta) and cross the placental barrier [412,413,414], potentially leading to fertility disorders. Pechcińska and co-authors [349] suggest that microplastics could disrupt embryogenesis by inducing oxidative stress, abnormal energy metabolism, and immunological disturbances, though this requires further investigation.

Microplastics can influence the gut microbiota in various ways, such as colonising microbial surfaces and leaching chemicals that affect microbial growth and diversity [415]. These particles can modify the composition of the gut microbiota, leading to dysbiosis and impaired function, thereby fostering numerous diseases [218,223,416,417,418]. Actinobacteria constitute a minor component of the gut microbiome, but this phylum includes genera capable of biodegrading polypropylene, polylactic acid polymer, polyurethane, and polyethylene. It has been demonstrated that polylactic acid can be degraded by the following actinobacterial genera: *Actinomadura*, *Amycolatopsis*, *Kibdelosporangium*, *Micromonospora*, *Nocardiopsis*, *Nonomuraea*, *Saccharothrix*, *Streptoalloteichus*, *Streptomyces*, *Thermomonospora*, and *Thermopolyspora* [419]. This suggests a potential dual role for Actinobacteria in maintaining gut health and contributing to microplastic biodegradation, although further research is required [349]. Furthermore, microplastics serve as carriers of hazardous substances, such as phthalates, bisphenol A, and heavy metals [340], which may also disrupt hormonal balance, promote carcinogenic processes, or exert toxic effects on the reproductive system.

In light of the evidence that microplastics accumulate in the human body and can exert adverse health effects, there is an urgent need to design and implement mitigation strategies. In the context of honey production, regulations should focus on limiting the use of the hazardous additives and packaging in plastic containers, as well as establishing limits for microplastic content in honey and other bee products.

## 4. Biological Contaminations of Bee Products

### 4.1. Microbiological Contamination

From a microbiological perspective, honey is considered a highly safe food product. This is primarily attributed to its unique antimicrobial properties, which include hyperosmolarity, low water activity, an acidic pH, as well as the presence of enzymes (glucose oxidase, lysozyme) as and apidaecins. The latter are proline-rich peptides that are secreted into honey by the hypopharyngeal glands of honey bees (*A. mellifera*) and inhibit the growth of various bacteria, particularly Gram-negative species [420]. As a result, most microorganisms are unable to proliferate and vegetative cells may not survive in the honey matrix for more than 30 days. Therefore, the presence of a considerable number of vegetative bacteria in honey indicates recent contamination [220,249,421,422].

The microbiota of honey can originate from various sources, which are typically categorised as primary or secondary. Primary sources include both mutualistic and pathogenic microorganisms inhabiting the bee gut, as well as environmental microbiota derived from soil, air, dust, plants, and flowers, which nectar, pollen, and plant sap are collected from by bees. Secondary sources, in turn, are associated with the stages of honey harvesting, processing, and storage, involving contact with personnel, equipment, and production surfaces [423,424]. The most important and most frequently identified (both via culture and molecular methods) bacteria found in the gut of adult honey bees are *Snodgrassella alvi*, *Gilliamella apicola*, *Lactobacillus* sp., *Bifidobacterium asteroides*, and *Frischella perrara* [425]. Those bacteria exhibit symbiotic and probiotic effects and are responsible for maintaining homeostasis in the bees’ guts. They synthesize enzymes that degrade various carbohydrates, resulting in the breakdown of mono-, di-, oligo-, and polysaccharides [371]. Moreover, the presence of other bacteria, such as *Apibacter invictus*, *Apilactobacillus kunkeei*, *Bartonella apis*, *Bombella apis*, *Bombilastobacillus mellis*, *Commensalibacter*, *Enterobacter cloacae*, *and Pantoea* sp.), as well as fungi, including *Hanseniaspora uvarum*, *Ascosphaera apis*, *Penicillium* sp., *Cladosporium* sp. and *Starmerella bombicola*, has also been demonstrated in the gut of honey bees [426,427,428,429,430]. Initially, the honey microbiome consists of microorganisms originating from nectar, pollen, and the digestive tract of bees. However, as honey matures, most transient contaminants are eliminated. This process, characterised by acidification, water evaporation, and an increase in sugar concentration, favours the selection of osmotolerant, xerotolerant, and acidophilic microorganisms, leading to the establishment of a stable microbial community.

In mature honey, bacteria from the Lactobacillaceae and Bacillaceae families predominate, while members of the Enterobacteriaceae, Bifidobacteriaceae, Acetobacteraceae, and Microbacteriaceae families occur in smaller amounts [431,432]. The detection of bacterial genera such as *Enterococcus*, *Escherichia*, *Shigella*, *Vibrio*, *Propionibacterium*, *Staphylococcus*, *Faecalibacterium*, *Corynebacterium*, and *Campylobacter* in honey indicates secondary contamination due to non-compliance with hygiene standards and good apicultural practices [220,249,423]. The presence of *Salmonella* spp., *Shigella* spp. and *Escherichia coli* is usually linked to environmental contamination, polluted pollen, or inadequate decontamination of processing equipment and packaging materials. Staphylococci, particularly *S. aureus*, can be introduced into honey through human handling under unhygienic conditions. This pathogen can cause various infections, including pneumonia, pharyngitis, endocarditis, meningitis, and bronchitis [433]. Therefore, maintaining high hygiene standards during the production of honey and bee products and ensuring that they are appropriately packaged, transported and stored significantly impacts the microorganisms present in these products, including the potential presence of undesirable and pathogenic species.

Spore-forming bacteria of the genera *Bacillus* and *Clostridium* are commonly found in honey [434,435]. Although most species of spore-forming bacteria are non-pathogenic, some species, including *Bacillus cereus*, *B. licheniformis*, *B. pumilus*, *B. majavensis*, *B. amyloliquefaciens*, *C. botulinum*, and *C. perfringens*, are known to cause foodborne illnesses. The latter two species are of particular microbiological importance, as they are capable of producing multiple protein toxins. *C. perfringens* is an anaerobic bacterium found in soil, but it is also a component of the physiological intestinal microbiota of humans and animals. It produces several toxins, including α-, β-, ε- and ι-toxins; NetB; and enterotoxin CPE, which are associated with histotoxic infections and food poisoning. Natural reservoirs of *C. botulinum* spores include soil, sand, sediment, and dust, from which they may be transferred to the surfaces of flowers, pollen, and nectar [249,436]. The presence of these bacteria has been detected in both pollen and numerous honey samples [249,423,435]. While the risk of infection from consuming honey is low for adults, honey poses a dietary risk factor for infant botulism, caused by the botulinum neurotoxin produced by *C. botulinum*. Studies have shown that honey may contain *C. botulinum* spores [437]. However, the toxin itself is absent due to honey’s high osmotic pressure and intrinsic antimicrobial properties. Infants, lacking a fully developed intestinal microbiota and possessing an immature immune system, are particularly susceptible to intestinal colonisation by *C. botulinum*, which can lead to a toxic infection known as infant botulism [438]. This disease, occurring in children under 12 months of age, results from the germination of *C. botulinum* (and occasionally *C. butyricum* or *C. baratii*) spores in the intestinal tract, followed by the in situ production of botulinum neurotoxin [436]. Once absorbed, the toxin is transported via the bloodstream to neuromuscular junctions, where it inhibits the release of acetylcholine, resulting in muscular paralysis. The average incubation period is between 2 and 4 weeks, with symptoms including a weak suck reflex, constipation, lethargy, hypotonia, respiratory distress, and ptosis [220,439].

The composition and abundance of the microbiota identified in various honey samples depend on the plant species from which bees collect nectar and pollen. Studies have shown that fir and cotton honey have significantly greater microbial diversity than fir-oak honey and *Arbutus unedo* honey [431]. The dominant bacteria identified in the analysed fir honey were from the genera *Lactobacillus* (primarily *Apilactobacillus kunkeei*), *Bradyrhizobium*, and *Pseudomonas*, accounting for over 40% of the total bacterial species identified. In contrast, cotton honey was dominated by *Pseudomonas*, *Streptococcus*, *Lysobacter*, and *Meiothermus* (nearly 40% of the total bacterial count), while in honey from *A. unedo*, the top three genera were *Lysobacter*, *Chryseobacterium*, and *Paenibacillus* (almost 60%). Since the microbiota profile depends heavily on the species of nectar-producing plant, it can therefore be used as an indicator of honey authenticity. This is particularly important in an era of adulterated single-flower honeys and the addition of samples from heavily polluted areas to honey from organic regions.

In addition to bacteria, honey and other bee products also contain yeasts and moulds. Yeasts, especially osmophilic species, are the dominant fungi in honey and are primarily represented by *Zygosaccharomyces*, *Debaryomyces*, and *Candida* [432,434]. The most commonly isolated moulds belong to the genera *Aspergillus*, *Fusarium*, *Cladosporium*, *Alternaria*, and *Penicillium* [290,432,440]. As described in chapter 2.6, these fungi are characterised by their ability to synthesise a wide range of mycotoxins, which are well known for their harmful impact on human health.

### 4.2. Allergens

Allergens are usually harmless substances, but they can induce specific IgE-mediated type I hypersensitivity reactions in hypersensitive individuals. Allergens can be divided into outdoor or indoor, extrinsic or intrinsic, as well as biological or chemical allergens [441]. Outdoor allergens can come from biological sources such as pollen and moulds [442]. Indoor allergens are generated by dust mites, dogs, cats, cockroaches, and moulds. Indoor allergens have a particularly profound impact on the human body because we spend up to 90% of our time indoors [443]. Allergens can also be categorised according to how often they cause allergic reactions. This classification helps determine which allergens are the most dangerous. These include plant pollens, house dust mites (*Dermatophagoides pteronyssinus*, *D. farinae*), pets (more specifically, the proteins present in their saliva, skin and fur), fungal spores, food allergens (e.g., milk, egg, nut, fish, soy, wheat proteins), drugs, insect venoms *Hymenoptera*, and natural latex, as well as environmental pollutants (suspended dust, ozone, nitrogen oxides) that can modify the structure of allergens and increase their immunoreactivity [444,445,446].

The most common inhalant allergens are the pollens of wind-pollinated plants, such as birch (Betulaceae), ragweed (*Ambrosia artemisiifolia*) and mugwort (*Artemisia vulgaris*). However, allergic symptoms can also be triggered by the pollens of insect-pollinated plants [447]. The release of inhalant allergens varies seasonally. Pollen released from trees (such as birch, cedar, oak, and pine) is dominant in spring, while pollen from grasses is primarily released in the late spring and summer. Pollen from weeds (such as ragweed, sagebrush and pigweed) occurs mainly in the late summer and autumn [111,443]. This means that bees visiting plants for nectar at a particular time of year are also more likely to collect pollen from those same plants. Honey bees then transfer this pollen to the hive, where it undergoes various transformations to produce bee pollen or perga. Therefore, bee pollen contains a large number of pollen grains (some sources state 400,000–6,400,000 per gram), which are produced by various allergenic plant families [448]. It has been proven that, despite the impact of bee salivary enzymes, the plant pollens present in bee pollen or perga retain their allergenicity [448,449]. Consequently, it poses a real risk to consumers who are allergic to certain types of plant pollen [450,451]. In monofloral honeys, pollen from the same plant predominates. For example, *Brassica napus* pollen was found in rapeseed honey, *Tillia* sp. pollen in linden honey, *Calluna* sp. pollen in heather honey, and *Fagopyrum* sp. pollen in buckwheat honey. However, pollen from other plants, such as Asteraceae, *Anthiscus*, and *Phacelia*, was also present [452]. Moreover, pollen from anemophilous plants such as *Salix*, *Pinus*, *Betula* and *Artemisia* was also detected in monofloral honeys [452].

Plant allergens are most commonly proteins or glycoproteins with a molecular weight of 5–80 kDa; however, low-molecular-weight chemicals, such as haptens (<5 kDa), can also act as allergens [453]. Group 1 allergens, also known as β-expansins, are recognised as the main allergens in grass pollen, with a high frequency of serum IgE reactivity. Allergens related to the major birch pollen allergen Bet v 1 are widely distributed, and they are produced by trees belonging to the Fagales order. These are PR-10 proteins, whose function is the transport of lipid hormones within the plant. The main allergens in ragweed pollen (*A. artemisiifolia*) are Amb a 1, Amb a 2, and Amb a 11. They are classified as pectin lyases, which are also found in other perennials, weeds and even deciduous trees, where they constitute Group 1 allergens. The most sensitising allergens in this group include Cup s 1 (from *Cupressus sempervirens*), Cup a 1 (from *Cupressus arizonica*), Art v 6 (from mugwort, *A. vulgaris*), Cry j 1 (from Japanese cedar, *Cryptomeria japonica*), and Jun a 1 (from mountain cedar, *Juniperus ashei*) [454]. The major pollen allergens from the olive tree (*Olea europaea*) are Ole e 10 (10.8 kDa) and Ole e 9 (46.4 kDa) and Ole e 1 (18–20 kDa) [455,456,457]. As Ole e 10 shares IgE epitopes with other plant proteins, it is considered a panallergen, exhibiting cross-reactivity with pollens from the Oleaceae, Gramineae, Betulaceae, Cupressaceae and Asteraceae families [458]. Ole e 1, on the other hand, is similar to the major allergen of *Plantago lanceolata* (Pla l 1), which is associated with hay fever [459]. In addition to pollen from plants, mould spores (mainly from the genera *Aspergillus* and *Cladosporium*) have also been found in bee products and can trigger severe allergic reactions [460,461].

For people with a genetic predisposition to allergies, pollen components trigger a cascade of specific immunological responses. Upon initial entry into the body, the allergen is captured and processed by antigen-presenting cells (APC), primarily dendritic cells. They subsequently present the processed allergen via major histocompatibility complex class II (MHCII) molecules to T-cell receptors (TCR) on naïve T-helper cells, favouring their differentiation into Th2 cells. When activated, these Th2 cells provide the necessary signals to B lymphocytes that stimulate them to differentiate into plasma cells. These cells preferentially produce immunoglobulin E (IgE) antibodies rather than IgG antibodies. Allergen epitopes are specific protein segments recognised by either the T-cell receptor (when presented on MHCII) or surface-bound IgE on B lymphocytes. The newly synthesised IgE antibodies enter the circulation and bind to FcεRI receptors on mast cells and basophils, thereby sensitising the individual. Subsequent exposure to the allergen results in multivalent cross-linking of the allergen to at least two adjacent receptor-bound IgE molecules. This cross-linking event triggers immediate degranulation, stimulating the release of histamine and other inflammatory mediators [462]. This leads to serum leakage, smooth muscle contraction, and a variety of clinical symptoms, including urticaria, angioedema, allergic rhinitis, lacrimation, bronchospasm, asthma, diarrhoea, hypotension, and anaphylaxis [453].

Numerous scientific studies indicate that allergic reactions can develop and intensify in sensitised individuals throughout their lifetime. This clinical progression is often referred to as the ‘atopic march’ and typically involves progressing from an asymptomatic sensitised state to a clinically manifested disease. In some cases, it can even result in life-threatening anaphylaxis [443,463].

From the perspective of allergic reactions, the occurrence of cross-reactions between allergens derived from different plant species is of paramount importance. Cross-reactivity arises from similarities in the structure of proteins found in the pollen of different plants, or due to the presence of so-called panallergens [448]. Some of the most extensively studied and thoroughly understood panallergens characterised by strong cross-reactivity are found in the pollen of plants belonging to the Asteraceae family, such as belonging to profilin family Amb a 8 from ragweed and Art v 4 a from mugwort [464,465]. Mugwort pollen cross-reacts with pollen from several plants that are popular with bees, such as sunflowers (*Helianthus annuus*), dandelions (*Taraxacum officinale*) and goldenrod (*Solidago virgaurea*) [111]. Other examples include Bet v 2 (birch pollen allergen from *Betula verrucosa*) that cross-reacts with pollen from plants belonging to various taxonomic groups, including apple (*Malus*), celery (*Apium graveolens*) and soya (*Glycine max*). Pollen from sunflower (*Helianthus annuus*), often found in honey, shows cross-reactivity with pollen of mugwort, dandelion, goldenrod, as well as with marguerite (*Chrysanthemum leucanthemum*) pollen [111,119,457,465,466]. Furthermore, cross-reactivity between airborne allergens (such as plant pollen, fungal spores, house dust mites and animal dander) and food has frequently been observed in patients with respiratory allergies. This may result in a food allergy, with symptoms ranging from mild mouth irritation to severe anaphylaxis [454]. The most commonly observed cross-reactions include birch–apple, celery–mugwort–spices, mugwort–peach, mugwort–chamomile, mugwort–mustard, cypress–peach, ragweed–melon–banana, quinoa–melon, *Alternaria*–spinach, house dust mites–prawns, cat–pork and bird–egg [454,457,465,467,468,469,470].

Consequently, consuming food products containing pollen grains from specific plants, such as honey, bee pollen and royal jelly, may trigger food allergy symptoms in patients with a respiratory pollen allergy. It is therefore essential to obtain a detailed medical history and check whether patients admitted to the intensive care unit with severe anaphylactic reactions to honey and bee products have experienced previous allergic reactions. Primary IgE-mediated sensitisation to inhaled allergens, which manifest as respiratory symptoms, can result in food allergies when the body is exposed to allergenic components that cause cross-reactions.

Allergic reactions to honey or bee products are extremely rare, with a prevalence of less than 0.001% in the general population **[471].** However, scientific literature has reported documented cases of symptoms ranging from mild rashes to anaphylactic shock. Table A1 (in Appendix A.1) summarises the reported cases of anaphylactic reactions and allergies to honey, bee pollen, propolis, bee bread, and other bee products. Allergies are usually associated with proteins found in pollen from various plants or in secretions from bees. Major Royal Jelly Protein 1 (MRJP1) is a dominant protein found in honey and royal jelly. Other potential honey allergens include phospholipase A2 (Api m 1, 16 kDa), hyaluronidase (Api m 2, 44 kDa), acid phosphatase (Api m 3, 43 kDa), melittin (Api m 4, 3 kDa), icarapin (Api m 10, 50–55 kDa), and vitellogenin (Api m 12, 200 kDa), as well as honey bee venom (HBV), venom dipeptidyl peptidase IV (Api m 5, 100 kDa), Api m 7 (CUB serine protease, 39 kDa), MRJP8 (Api m 11.0101a) and MRJP9 (Api m 11.0201a) [444,452,472,473,474,475].

The allergens present in honey, bee pollen and bee bread are essentially the same as those found in plant pollen but they are usually found in much lower concentrations. The lactic fermentation process that leads to perga formation of bee pollen may cause the hydrolysis of certain proteins. This is probably why no serious reactions have been reported to date following the consumption of bee bread to date. However, although fermentation reduces the allergenicity of perga compared with raw bee pollen [476], it cannot be guaranteed that all allergenic particles have been removed. Various studies have revealed that bee bread contains the pollen grains from numerous plant taxa, depending on the geographical region in which the honey bees are active. Pollens from the following plant families were usually predominant: Asteraceae, Brassicaceae, Convolvulaceae, Euphorbiaceae, Fabaceae, Lamiaceae, Malvaceae, Mimosaceae, Plantaginaceae, Poaceae, Rosaceae, Rubiaceae, Sapindaceae, and Tiliaceae [477,478,479,480]. Consequently, there is still a risk of an allergic reaction in particularly sensitive individuals.

Generally, bee pollen causes the most allergies, followed by honey and royal jelly [451]. The reactions they induce are usually IgE-mediated and manifest as anaphylaxis, itching, urticaria, angioedema, as well as asthma and dermatitis (see Table A1). Propolis, wax, and bee venom are generally applied topically in ointments and cosmetics and cause allergies the least frequently, though cases have been reported [481,482,483,484]. Their mechanism of action is T-cell-mediated or IgE-mediated, manifesting as contact dermatitis or, in more severe cases, anaphylaxis [485,486,487,488,489,490]. Dental technicians and beekeepers are believed to be occupations that are more prone to allergic reactions to propolis [491,492]. To date, several compounds with strong sensitizing properties have been isolated from propolis; these compounds are responsible for the development of contact hypersensitivity. The most important compounds in propolis include caffeic acid esters (1,1-dimethylallyl caffeic acid ester, benzyl caffeic acid ester, cinnamyl caffeic acid ester, caffeic acid phenethyl ester (CAPE), benzyl ester of isoferulic acid, benzyl ester of ferulic acid, phenethyl ester of isoferulic acid, cinnamoyl ester of isoferulic acid, 3-methyl-2-butylene ester of isoferulic acid, 3-methyl-2-butenyl caffeine, phenylethyl caffeine, benzyl caffeine, geranyl caffeine, benzyl cinnamate and benzyl alcohol, methyl cinnamate, ferulic acid, benzoic acid, cinnamic acid, vanillin, nerolidol, and tectochrysin) [486,490,493,494,495].

An allergy to honeybee venom (HBV) is one of the most common causes of anaphylaxis. HBV contains several bioactive components, including allergens such as melittin (Api m 4), phospholipase A2 (Api m 1), acid phosphatase (Api m 3) and hyaluronidase (Api m 2), to name a few [496,497]. HBV also contains histamine, which causes pain and lowers blood pressure, as well as dopamine and catecholamines, which are highly cytotoxic locally. The main HBV allergens cross-react with IgE, potentially yielding false-positive results in allergy diagnostics. As eight of the allergens identified in honey bee venom have also been found in honey, adverse and allergic reactions after consuming honey are sometimes observed in allergic individuals [444].

The anti-allergic properties of honey, bee pollen and royal jelly mean that people very often turn to these products to alleviate their symptoms [498,499,500]. However, for individuals with particularly sensitive immune systems, the components present in these products pose a potential risk of an allergic reaction, ranging from mild symptoms to severe anaphylaxis. As allergic reactions could occur even after the first RJ intake, the cross-reactivity with other allergens is the more probable cause. Main allergens in RJ are MRJP1, MRJP2 and MRJP3, which exhibit cross-reactivity with European (*Dermatophagoides pteronyssinus*) and American (*D. farinae*) house dust mite, snow crab (*Chionocetes* spp.), edible crab (*Cancer pagurus*), German cockroach (*Blatella germanica*), and *Apis mellifera* venom [501]. This can result in RJ allergy in atopic dermatitis individuals.

To improve the safety of bee products, further research is needed regarding the allergens found in them, particularly in honey, bee pollen and royal jelly. A broad public awareness campaign about the potential risks is also required. This campaign should primarily target individuals with inhalant allergies, as they tend to react more strongly to bee pollen in skin tests. They may experience symptoms typically associated with food allergies after consuming bee products due to the cross-reactivity of pollen allergens [448]. Other target groups include individuals allergic to HBV and caregivers of young children. It is also worth noting that if a patient’s allergic reaction is triggered by plant pollen in honey rather than honey proteins themselves, they may exhibit greater tolerance when consuming highly filtered, commercially available honey. Despite obvious allergens derived from plants and bees themselves, honey may contain other allergens, including those resulting from treatments used by beekeepers. Gluten, milk, or soy allergens can end up in honey if the ingredients in supplements or supplemental feed intended for honey bees are added by beekeepers [502].

### 4.3. Phytotoxins

Plants synthesise a wide range of secondary metabolites, which are found in various tissues, such as fruits, flowers, pollen and nectar. These compounds exhibit various biological properties and are a key element in adaptive and defensive strategies against biotic and abiotic stressors. They can act as attractants for pollinators or protective agents against herbivores, fungi and bacteria. To date, over 100,000 plant secondary metabolites (PSMs) have been identified. While many of these compounds have beneficial health effects and are used in pharmaceuticals, many others are highly toxic to humans, even in small quantities. Adverse effects include allergenic, pesticidal, hallucinogenic and allelochemical properties, and exposure to high concentrations can even lead to death. These toxic PSMs are called phytotoxins [503]. Phytotoxins are characterised by great diversity in terms of their toxic mechanism of action, chemical composition and structure. The most significant phytotoxin groups include alkaloids, tannins, terpenoids, glycosides, phenolic compounds, flavonoids, non-protein amino acids, glucosinolates, cardenolides and phytoecdysteroids.

Given that nectar and pollen serve as primary or secondary raw materials in the production of bee-derived products, it is essential to recognise that these products may contain toxic PSMs in quantities that could pose a potential health risk to consumers. Pyrrolizidine alkaloids (PAs) are the most extensively studied group of naturally occurring toxins found in bee products [23,504,505,506]. However, other toxic compounds have also been detected in certain types of honey, including tropane alkaloids, grayanotoxins, matrine-type alkaloids, gelsedine-type alkaloids, tutin, and triptolide [507,508,509,510,511]. In addition, pollen and nectar from certain plant species may contain other alkaloids and toxic metabolites, such as nicotine, scopolamine, and aconitine [512,513].

PAs are structurally diverse compounds that occur naturally in around 3% of flowering plant species worldwide. They are most commonly synthesised by plants belonging to the *Anchusa*, *Heliotropium*, *Echium*, and *Borago* genera (Boraginaceae); the *Crotalaria* genus (Fabaceae); and the *Senecio*, *Tussilago*, *Eupatorium* and *Eupatorium* genera (Asteraceae) and have also been identified in the *Muscari*, *Scilla*, *Argyreia*, *Cremastra*, *Liparis*, *Amphorogyne*, and *Osyris* genera from other families. The highest concentrations of PAs are reported in flowers and seeds, whereas lower levels are typically found in leaves, stamens and roots [504,505,514,515].

Structurally, pyrrolizidine alkaloids are esters containing a necine base (in the form of platynecine, retronecine, heliotridine or otonecine) and one or two necic acids. These can undergo chemical modification, such as hydroxylation, dehydrogenation, halogenation, and acetylation, which increases the number of potential PAs derivatives and their high structural diversity. Additionally, the nitrogen atoms in the necine moiety can be oxidised leading to the formation of N-oxides, which coexist with PAs in most plants. To date, more than 600 pyrrolizidine alkaloids and their corresponding N-oxides have been identified in thousands of plant species [505,514,515]. Based on their structural features and botanical origins, PAs are generally classified into four main types: heliotridine-type (e.g., lasiocarpine and europine), lycopsamine-type (e.g., echimidine and intermedine), monocrotaline-type (e.g., monocrotaline and fulvene), and senecionine-type alkaloids (e.g., senecionine, retrorsine and jacobine) [516].

Toxicological studies suggest that PAs exhibit greater toxicity when the necine base contains an unsaturated bond at the 1,2-position and is esterified with a branched necic acid [113,506]. Exposure to PAs can result in both acute and chronic toxic effects. Acute poisoning from these compounds is primarily associated with liver damage resulting from the hepatotoxic effects of reactive metabolites formed during hepatic bioactivation, which can result in liver enlargement (hepatomegaly) and ascites [504,515]. Chronic, low-level exposure, on the other hand, has been linked to genotoxic, mutagenic, and carcinogenic effects, representing a significant health risk to humans. This is driven by the primary metabolites of PAs, dehydropyrrolizidine alkaloids (DHPAs), which are then hydrolysed to dihydropyran derivatives (DHPs). Both DHPAs and DHPs are able to interact with DNA and proteins, forming various adducts and crosslinks [506,517]. Of the many PAs, riddelliine, lasiocarpine, and monocrotaline have been classified by the International Agency for Research on Cancer (IARC) as possibly carcinogenic to humans (Group 2B) [505]. This classification is based on experimental evidence demonstrating their ability to induce DNA damage and promote tumourigenic transformations in animal models.

The toxicity of PAs primarily depends on their chemical structure, metabolic pathways, detoxification rate, and individual physiological factors. Toxicity only occurs when there is a double bond between positions 1 and 2 and when there are ester groups attached at positions C-7 and/or C-9 of the necine base. No RfD or TDI has been established for these compounds; instead, the MOE is used to calculate risk, although, compared to the hazard index method, the MOE takes into account more factors of uncertainty. The BMDL10 values, which EFSA set at 70 μg/kg bw per day in 2017 [518], and the EDI are usually used in these calculations. The calculations carried out indicated that for certain populations (particularly toddlers), the calculated MoE values for the long-term consumption of honey are below 10,000. In contrast, the acute toxicity of PAs was found to pose no risk in relation to honey consumption. Similarly, acute PA exposure caused by the consumption of pollen-based supplements showed low exposure estimates in the range of 3–44 ng/kg bw per day; therefore, the consumption of pollen-based supplements is also not considered to pose an acute risks to human health.

Pyrrolizidine alkaloids are considered protoxins. Thus, in order to exert their harmful effects, they must be activated through hepatic metabolism via normal oxidative detoxification mechanisms. In this process, tertiary alkaloids are converted into pyrrolic metabolites by the cytochrome P-450 monooxygenase enzyme system. Conversely, the non-toxic N-oxide forms of pyrrolizidine alkaloids may be reduced to their corresponding toxic tertiary alkaloids following ingestion, absorbed, and subsequently activated by the liver. The toxic effects of pyrrolizidine alkaloids stem from their interference with neuronal signal transmission by interacting with neuronal receptors, ion channels, and the enzymes responsible for degrading neurotransmitters and secondary messengers. Furthermore, they can intercalate into DNA, inhibit protein synthesis, induce apoptosis, and suppress the activity of enzymes involved in carbohydrate metabolism [505,515]. PAs can also induce pulmonary venous obstruction and blood vessel damage, while long-term exposure to PAs can result in kidney, gastrointestinal tract, bone marrow and pancreatic damage. This can contribute to the development of megaloblastic anaemia and inhibit mitosis. Furthermore, alkylating agents formed by the biotransformation of PAs in the body can act synergistically with aflatoxins and the hepatitis C virus (HCV), contributing to the development of various liver cancers [504,506,515,519,520,521].

Bee products are a considerable dietary source of pyrrolizidine alkaloids, as the nectar and pollen collected by bees from certain plant species may contain high concentrations of these toxins. The most commonly identified PAs in beekeeping products are senecionine, echimidine, and lycopsamine. Studies indicate that these PAs are frequently detected in bee products, particularly those derived from the pollen and nectar of plants belonging to the *Echium*, *Senecio*, *Eupatorium*, and *Borago* genera, which are widespread and heavily visited by bees [504].

The concentration of PAs differs between honeys from various regions. A mean value of about 10 µg PAs per kg of honey samples was reported for samples from Berlin [522]. Among 50 honey samples collected in Poland in 2017, at least one pyrrolizidine alkaloid was identified in 16 by LC-MS [523]. The concentration of these PAs ranged from below the limit of quantification (LOQ) of 1 µg/kg to 52.4 µg/kg. The PAs detected most frequently were echimidine (in 43.8% of the positive samples), lycopsamine (31.3%), intermedine (25%), retrorsine (25%) and senecionine (19%). The QuEChERS sample extraction method, coupled with UPLC-MS detection, was used to quantify nine different PAs (echimidine, heliotrine, intermedine, lycopsamine, lasiocarpine, retrorsine, seneciphylline, senecionine, and senkirkine) in retail honeys of various origins purchased in Italian local supermarkets [524]. The PAs concentrations ranged from 1 to 169 μg/kg, with higher concentrations found in blends of European and non-European honeys and with many samples exceeding the tolerable daily intake (TDI) of PAs. The TDI for toxic PAs is usually assumed to be 0.1–1.0 μg/kg body weight per day, or 0.1–1.0 μg/day over a six-week period. Of the 50 retail honeys tested by Griffin et al. [525] one or more PAs have been detected in 16% of samples, with an average concentration of 1260 μg/kg.

Analyses have shown that honeys from southern Europe, specifically Italy and Spain, exhibit a higher frequency and higher concentrations of PAs than honeys from Bulgaria, Germany, Hungary, and Romania. This may be attributed to the greater prevalence of PA-producing plant species in the Mediterranean Basin’s flora. Furthermore, multifloral honeys generally contain higher concentrations of PAs than monofloral varieties [113].

When assessing PA content in 92 samples of commercially available honeys in Brazil, distinct PA profiles have been observed depending on the plant sources and geographic origins [526]. Depending on the compound analysed, between 7 and 84 honey samples yielded a positive result. The most abundant compounds were senecionine-N-oxide (8.1–248 µg/kg) and senecionine (20.8–180 µg/kg), which were identified in 92.3% and 84.6% of the samples, respectively. Other PAs identified included lycopsamine, intermedine, monocrotaline, retrorsine, retrorsine-N-oxide and echimidine. Further research on Brazilian retail honeys reported the presence of PAs in 39 out of 80 tested samples, primarily intermedine/lycopsamine (27% of the samples tested were positive), as well as erucifoline (17%) [527]. The highest concentration was revealed for senecionine (141.8 µg/kg). More than 99% of the samples contained at least three PAs.

PAs have been detected in 58% of the 120 samples of Chinese honey, with concentrations ranging from 0.04 to 288.07 μg/kg [528]. The predominant PAs were lycopsamine, echimidine and clivorine, with levels varying across regions of origin. Similar results were obtained by He et al., who analysed 255 honey samples from China [529]. Their study revealed that 58% of samples contained PAs (with total content ranging from 0.2 to 281.1 μg/kg), with lycopsamine, echimidine and monocrotaline being the most abundant (originating from *Senecio* spp., *Echium plantagineum* and *Crotalaria* spp., respectively). Interestingly, Moreira et al. [530] did not detect any PAs in one monofloral and two multifloral Portuguese honeys produced from *Echium plantagineum* L. Although, for some countries of honey origin (e.g., Brazil) [506], the MoE values were lower than the safe threshold of 10,000, it is worth highlighting the differences in methodology. In particular, whether the MoE is calculated for individual alkaloids or for their total, since each type of PA exhibits a different toxic potency, simply summing the concentrations of all PAs may result in both overestimation and underestimation of the risks.

Out of 48 tropical honeys originating from different agro-ecological zones in Ghana, 85% of the samples were positive for PAs, with the highest concentration being 2639 μg/kg [531]. As many as 3917 honey samples and 119 bee pollen samples were analysed for PAs in Dübecke’s study [532]. PAs were detected in 94% of retail honeys available in supermarkets, 66% of raw honeys (i.e., bulk honey not yet packaged in containers), and 60% of bee pollen samples. In another study, 44 samples of bee pollen available in Italian markets were evaluated for the presence of 17 PAs and their N-oxide derivatives (PANO) [533]. PAs/PANOs (mainly echimidine and echimidine N-oxide) were found in 77% of the samples, with concentrations ranging from 1.7 to 3356 μg/kg. The authors calculated that, if the average daily consumption of bee pollen is 5 g for a 60-kg adult and a PA dose of 0.007 μg/kg bw is recommended by the EFSA [534], then samples containing more than 84 μg/kg could pose a health risk.

The maximum concentration of the sum of PAs and PANO (Σ35PAs) in pollen-based food additives, pollen, and pollen products set by the European Union (EU) 2023/915 is 500 µg/kg [148]. Depending on the source of the pollen, numerous studies indicate that these levels may be exceeded many times over. The analysis of 15 samples of bee pollen and 16 samples of bee bread, all of which are commercially available in Brazil, revealed the presence of at least two pyrrolizidine alkaloids [20]. The most frequently identified were senecionine, N-senecionine oxide, monocrotaline and retrorsine. The total PA content ranged from 268 to 263,849 µg/kg, and, more importantly, as many as 77.4% of the samples exceeded the maximum limit set by the European Union (500 µg/kg). Among the 32 Swiss commercial bee pollen samples tested, 31% yielded positive results for PAs, primarily of the *Echium*- and *Eupatorium*-types, with an average concentration of 319 μg/kg [535]. The predominant PAs reported in pollen from *Echium vulgare* were echivulgarine and its N-oxide [536]. In turn, bee bread, aside echivulgarine and echivulgarine N-oxide also contained vulgarine, echimidine, and intermedine congeners. Much higher concentrations of PAs (reaching up to 37,855 µg/kg) were observed in bee-collected pollen samples of unknown origin [532] and PA concentrations up to 16,350 µg/kg have been reported for a pollen sample from Spain containing mainly *Echium* pollen [537]. Thanks to traps that researchers installed (between 15 July and 1 August 2019) in apiaries at 57 locations in southern Germany, they collected bee pollen, primarily from the species belonging to *Borago* sp., *Echium* sp., *Eupatorium* sp. and *Senecio* sp. [538]. Positive results for PAs were found in 52 out of 57 analysed samples, with concentrations of ΣPAs ranging from 0.48 to 48,400 μg/kg of bee pollen. The predominant compounds were echinatine-NO, rinderine-NO, lycopsamine-NO and intermedine-NO.

It has been demonstrated that only a small fraction of PAs from bee bread is transferred into the royal jelly of nurse bees, indicating the existence of a protective mechanism that shields larvae from toxins [539]. Royal jelly contains an average of 15.5 µg/kg of PAs, making it the safest bee product for human health [540].

Tropane alkaloids (TAs) constitute a group of naturally occurring phytotoxins identified in members of several botanical families, including Solanaceae, Brassicaceae, and Erythroxylaceae. These compounds are found in more than 3000 plant species and are responsible for the toxicity of several of them, such as henbane (*Hyoscyamus niger*), deadly nightshade (*Atropa belladonna*), Jimson weed (*Datura stramonium*), and other species belonging to the *Datura* genus. Tropane alkaloids are distributed throughout all plant parts, including fruits and flowers [541,542]. Therefore, there is a risk that bees foraging on toxic plants (primarily in the Solanaceae family) may collect pollen containing TAs. These plants may grow near crops intended for honey production, from which bees typically collect pollen and nectar, resulting in the contamination of honey and other bee products with tropane alkaloids.

Structurally, tropane alkaloids are characterised by a bicyclic tropane skeleton, comprising a pyrrolidine ring and a piperidine ring that share one nitrogen atom and two carbon atoms [542]. To date, more than 200 distinct structures have been identified, among which atropine, hyoscyamine and scopolamine are the most extensively studied [543]. The toxic mechanism of action of TAs is related to their ability to pass the blood–brain barrier and their antagonistic effect on muscarinic acetylcholine receptors, which leads to the inhibition of parasympathetic activity. Characteristic symptoms include reduced secretion of saliva, bronchial mucus, and sweat; mydriasis and accommodation paralysis; cardiac rhythm disturbances; urinary retention; decreased gastrointestinal motility; and reduced gastric acid secretion.

So far, contamination of bee products by TAs has been detected relatively rarely. Further, according to EU Regulation No. 2023/915 [148], tropane alkaloids are not subject to specific regulatory requirements for honey, pollen or other bee products. However, new methods are being developed and there are reports of TAs being detected in honey and bee products. Martinello et al. [544] developed and validated a quantitative method for determining atropine and scopolamine in honey samples. They found TAs in only two out of 47 samples of bee pollen tested. Out of 11 honeys tested by LC-HRMS, only one contained scopolamine (27 µg/kg); the other samples were negative for TAs [545]. Similar results were obtained by Thompson et al. [546], who examined 23 honey samples from different geographical regions for the presence of hyoscyamine and scopolamine. TAs were not detected above the LOQ (0.01 μg/kg) in Canadian and New Zealand honeys. Only two samples from other countries contained hyoscyamine at a level of 0.012 µg/kg, and one of these also contained 0.012 µg/kg of scopolamine. Another study [547] found that none of the 29 honey samples tested, which were of various botanical and geographical origins, contained detectable levels of atropine or scopolamine. Casado et al. [548] tested five commercial honey samples (rosemary, orange blossom, and multifloral) and two retail honeys (multifloral and woodland) purchased in Spain and Israel for the presence of TAs. All of the samples contained atropine, but only the rosemary honey sample contained scopolamine.

Grayanotoxins, also known as andromedotoxin, acetylandromedol, or rhodotoxin, are toxic diterpenes that occur naturally in the leaves, nectar, and pollen of certain plant species belonging to the Ericaceae family, including members of the genera *Rhododendron*, *Pieris*, *Agarista*, and *Kalmia* [507]. These compounds are polyhydroxylated cyclic diterpenoids and their ring system does not contain nitrogen. Approximately 20 different grayanotoxins have been identified in honey and related plant products, among which grayanotoxin I (andromedotoxin) and grayanotoxin III (andromedol) are considered the most toxicologically relevant to humans [549,550]. Plants producing grayanotoxins, particularly yellow rhododendron (*Rhododendron luteum*) and pontic rhododendron (*Rhododendron ponticum*), are widespread along the Black Sea coast, mainly in Turkey, in Nepal, and in some regions of North America, Europe, Japan, and Brazil. Another important source is alpine rhododendron (*Rhododendron ferrugineum*), naturally distributed in the Alps and the Pyrenees. Some beekeepers deliberately produce monofloral honeys derived from these plants, commonly known as ‘mad honey’. This product is traditionally harvested and consumed in Turkey, although cases of poisoning have also been reported in other parts of the world [551]. Most toxicological cases involve honey exported from Turkey, although honey from Nepal has also been implicated. Some cases are accidental, while others are intentional and motivated by the alleged medicinal properties of the honey, such as its aphrodisiac, anti-inflammatory and therapeutic effects in conditions like gastric disorders, arthritis, diabetes, and hypertension [113,551].

The toxicity of ‘mad honey’ is dose-dependent. Usually, the ingestion of approximately 15 to 30 g may induce clinical symptoms of intoxication within half to 4 h after exposure. Acute grayanotoxin poisoning affects the muscular, nervous, and cardiovascular systems. The toxic mechanism involves the failure to inactivate neuronal sodium channels, leading to prolonged opening and the sustained depolarisation of neuronal membranes. This results in excessive vagal nerve stimulation, which underlies the characteristic clinical manifestations. Fatal cases of ‘mad honey’ intoxication are now exceedingly rare due to effective symptomatic management, typically involving intravenous atropine and electrolyte infusion therapy. It should be emphasised that the toxicological risk of consuming honey containing grayanotoxins is considerable, as their concentrations are strongly influenced by environmental, climatic, and seasonal factors, making their toxic potential unpredictable [507,549,551,552,553]. As lower doses of grayanotoxin can cause symptoms such as dizziness, excessive sweating, salivation, nausea, vomiting, blurred vision, seizures, paraesthesia, hypotension and mild bradycardia, these may be mistaken for an allergic reaction. In severe cases of poisoning, symptoms resembling anaphylactic shock may occur, including bradycardia, facial angioedema, loss of consciousness, cardiac arrhythmia, respiratory failure, hypoxia and death if medical assistance is unavailable.

Matrine alkaloids (e.g., matrine and oxymatrine) are a class of naturally occurring quinolizidine alkaloids found in plants of the genus *Sophora* (family Fabaceae), particularly *Sophora flavescens* and *S. alopecuroides*. To date, over 70 distinct matrine-type structures have been isolated from these species. These plants play an integral role in Traditional Chinese Medicine (TCM), and contemporary pharmacological and clinical studies confirm the broad spectrum of biological activity of these compounds. Matrine-type alkaloids exhibit anti-tumour, anti-inflammatory, antimicrobial, hepatoprotective, cardioprotective, neuroprotective and antioxidant effects [554]. However, increasing evidence highlights their toxicological potential, including hepatotoxicity, neurotoxicity, and developmental toxicity in animals [555,556]. These compounds may enter the human food chain incidentally, for instance, through honey derived from *Sophora* nectar. Wang et al. [557] conducted a large-scale study analysing the concentrations of four matrine-type alkaloids (matrine, oxymatrine, sophocarpine, and sophoridine) in 567 honey samples collected across China. Most of the samples were acacia (*Robinia pseudoacacia*) honey, although other monofloral and polyfloral honeys were also included. Matrine-type alkaloids were detected in 61% of the samples, with concentrations reaching 2774 µg/kg. Oxymatrine was the most frequently detected compound (58%), followed by sophocarpine (44%), matrine (37%), and sophoridine (2%). Investigating 112 jujube honey samples, Li et al. found that 62.5% contained matrine and oxymatrine, with concentrations ranging from below the detection limit to 319.3 μg/kg [558].

*Gelsemium* alkaloids are a group of natural compounds present in plants of the *Gelsemium* genus, such as *Gelsemium elegans* (commonly known as ‘heartbreak grass’) and *Gelsemium sempervirens* (Carolina jessamine). *G. elegans* is native to Asia and is widely distributed in southern China, while *G. sempervirens* occurs naturally in Mexico and Central America, where it is often cultivated as an ornamental plant. More than 120 alkaloids have been isolated from *Gelsemium* species, primarily from the stems, leaves, and roots. The most prevalent type included indole alkaloids and hydroxyindole alkaloids. The main monoterpenoid indole alkaloids are gelsedine, gelsemine, humantenine, koumine, sarpagine, and yohimbine. Koumine and gelsemine are particularly relevant from a toxicological perspective due to their high concentrations in plants, rapid absorption, and widespread tissue distribution in humans [559]. These compounds exert neurotoxic, enterotoxic, and respiratory effects, leading to disturbances in the nervous, gastrointestinal, and respiratory systems. Symptoms of acute poisoning can include dizziness, vomiting, abdominal pain, convulsions, coma, and muscle spasms and can result in death in severe cases [560,561]. Conversely, *Gelsemium* species have been used in alternative medicine, where they are believed to have anti-anxiety, analgesic, anti-inflammatory, anti-neoplastic, and immunomodulatory properties, suggesting their potential use in the treatment of anxiety-related mental disorders [562].

The pollen of *G. elegans* can be collected by honey bees while they are foraging. This pollen can then be incorporated into honey, leading to the production of toxic honey, which poses a potential threat to consumer health and safety [563]. The qualitative and quantitative composition of alkaloids in bee products derived from *Gelsemium* is similar to that found in its flowers and buds, but differs markedly from that found in the other parts of the plant [564]. Research on *Gelsemium* alkaloids in bee products is rare and is currently limited to Chinese samples. The concentrations of gelsemine, koumine and humantenmine in all 30 honey samples tested were below LOQ, probably due because the honey was collected outside the *Gelsemium* blooming period [565]. However, another study found 14-(R)-hydroxy-gelsenicine (HE), along with gelsemine, gelsenicine, and humantenirine, in honeys collected in southern China [566]. HGE accounted for over 80% of the total toxic components, with a mean concentration of 17.2 μg/kg. The estimated LD_50_ is 0.125 mg/kg and 0.295 mg/kg for the female and male mice, respectively.

Other interesting phytotoxins that have been found in bee products include nicotine, triptolide and tutin [567,568,569]. Nicotine, a highly toxic alkaloid found in tobacco (*Nicotiana* spp.), may also be present in the pollen and nectar of other plants visited by honey bees and other pollinators [570]. Nicotine is a known bees’ feeding deterrent due to its bitter taste. Moreover, it can also be incorporated by plants from tobacco smoke and traditional nicotine-based products, which are used as insecticides to control sucking and chewing pests [571]. The samples of fresh honey obtained from Tanzanian apiaries located within tobacco growing areas contained a significantly higher nicotine content compared to samples from non-tobacco growing areas (4600 μg/kg vs. 2600 μg/kg). The authors also reported that honey samples from town vendors contained less nicotine (4100 μg/kg) than those from villages vendors (29,800 μg/kg). Slightly lower results were obtained for Palestinian honeys from various regions of the West Bank [572]. Nicotine was detected in 67% of honey samples (14 out of 21), with concentrations ranging from 178 to 9389 µg/kg, and highest values were also reported for honey from beehives localised near tobacco plantations. Nicotine has detrimental effects on all the major physiological systems in the human body, including the cardiovascular, respiratory, renal and reproductive systems. Its carcinogenic properties and role in tumour promotion are well documented [568].

Triptolide is a diterpenoid belonging to the triepoxide group. It is found in the pollen, roots and leaves of *Tripterygium* species, particularly *T. wilfordii* and *T. hypoglaucum*, which are commonly known as thunder god vine. These plants have long been used in traditional Chinese medicine to treat a wide range of diseases, though precise dosing is crucial to avoid toxicity. Triptolide is considered to be the main biologically active compound in these plants, alongside other diterpenoids and alkaloids, such as wilforgine, wilforine, euonine, and peritassine A [569]. The initial symptoms of triptolide poisoning initially include nausea and vomiting, followed by abdominal pain, diarrhoea, palpitations, dizziness, chest tightness, and dyspnoea. In severe cases, anuria, seizures, haematuria, gastrointestinal haemorrhage, and death may occur [573]. Three cases of fatal poisoning were reported in China and were related to the ingestion of raw wild honey by workers, leading to acute renal failure [574]. Honey poisoning associated with triptolide typically occurs in summer, coinciding with the flowering period of *Tripterygium* sp. It has been suggested that the triptolide content decreases during honey storage, which could explain the seasonal pattern of intoxication. Studies indicate that triptolide levels decline by about 50% within three months, likely due to the activity of oxidoreductase enzymes present in honey [569,575]. Perhaps this is why neither protopine nor triptolide was detected in any of the 90 analysed samples of commercially available honey [576].

Tutin is a neurotoxic sesquiterpenoid compound found in certain types of honey produced from the secretions (honeydew) of the passion vine hopper (*Scolypopa australis*). The insects feed on the sap of the New Zealand tutu shrub (*Coriaria arborea*) and honey bees can harvest honeydew containing tutin from the bark and leaves as a supplementary food source. This plant’s natural range is practically limited to New Zealand, where the presence of tutin and its hydroxy metabolite, hyenanchin (hydroxytutin), in honey led to numerous poisoning cases in 2008, sparking widespread concern [567]. Chemically, tutin is a polyoxygenated polycyclic sesquiterpene composed of two epoxide rings and a lactone, with a structure similar to that of picrotoxinin. It is an antagonist of γ-aminobutyric acid (GABA) receptors and an inhibitor of glycine receptors in neurons of both mammals and insects. Therefore, it disrupts normal neuronal signalling, resulting in neurotoxic symptoms. Clinical manifestations of tutin poisoning include nausea, headaches, vomiting and dizziness, while severe cases may involve seizures, loss of consciousness, coma, and death [567,577]. If honey bees collect the honeydew of *S. australis* in late summer, they transfer the toxin into the honey. Thus, beekeepers should make every effort to prevent the bees from feeding on this honeydew, particularly during the main risk period from January to April. Tutin has been found in five Manuka honeys originating from New Zealand and purchased in the United Arab Emirates, but only one sample had a concentration above the LOQ (0.0449 mg/kg) [578]. To minimise the toxicological risk to consumers, the Australia New Zealand Food Standards Authority (FSANZ) has set the maximum permitted concentration of tutin at 0.7 mg/kg in extracted and blended honeys [579].

## 5. Conclusions

The complex composition of various natural bee products can make them difficult to standardise. Nevertheless, they attract considerable interest from researchers and consumers alike. These products are valuable sources of bioactive compounds that can benefit human health, and they are widely used in medicine, food processing, and cosmetology. However, their composition must be monitored for various contaminants and their potential impact on human health, as these compounds may enter bee products from either the hive or the external environment. Currently, regulatory standards for hazardous compounds only apply to honey and rarely to bee pollen and propolis supplements. Consequently, clear, legally binding requirements are needed for the global trade of bee products to ensure their quality and safety. The risks associated with the presence of pesticide residues, antibiotics, or mycotoxins in various bee products may have a measurable impact on human health, and the current system of public health regulation is inadequate. Although there are national biomonitoring programmes that assess bee products and provide valuable information, they cannot be considered a global solution to the problem of pesticides. Microplastics, phytotoxins, antibiotics, pesticide residues and other types of contaminants continue to be detected in bee products.

**Figure 4:**
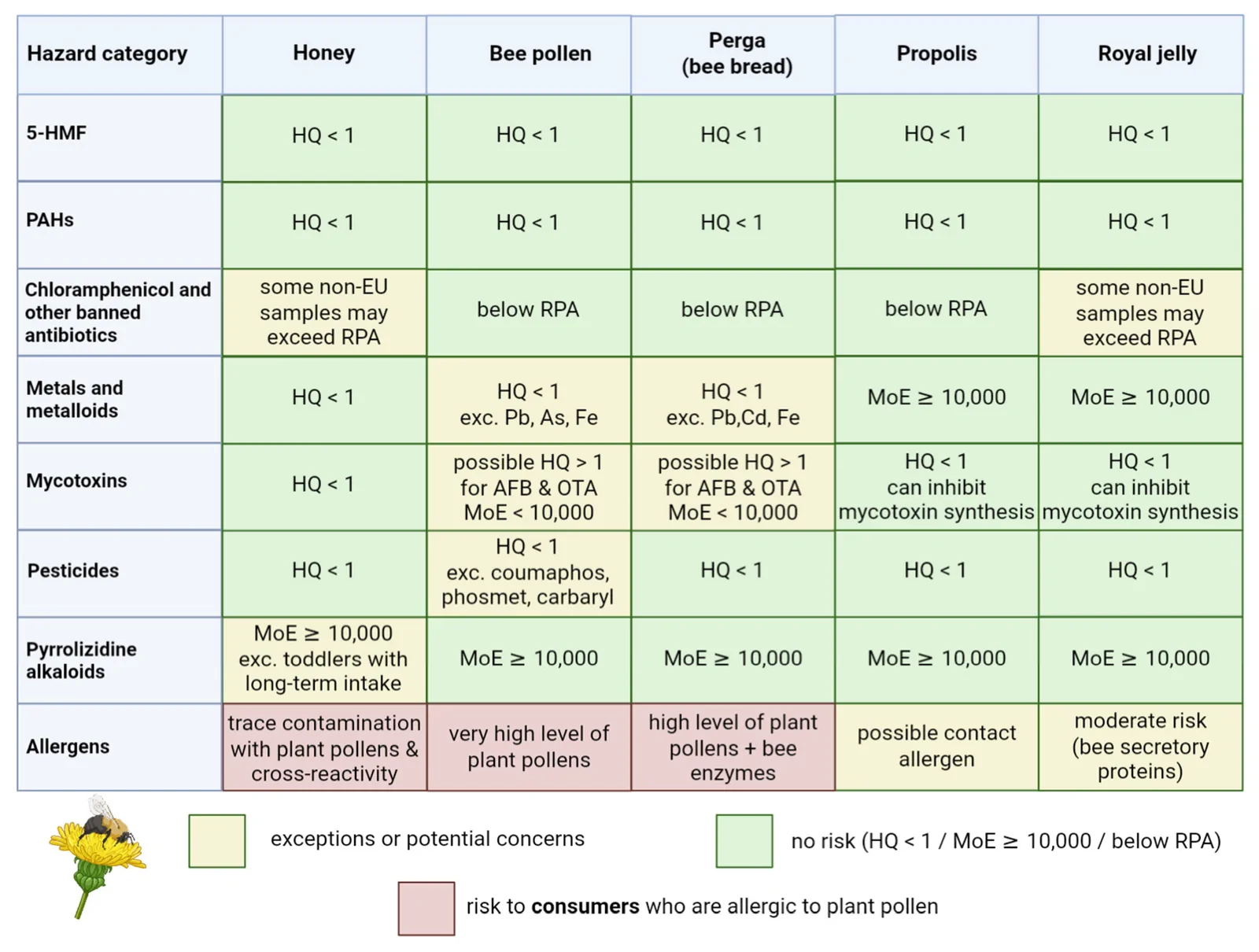
Risk assessment matrix for apicultural products. Green boxes mean that there is no health risk related with the consumption of bee product; yellow boxes highlight products of potential concern related with particular hazardous components (exceptions or threshold exceedances). Abbreviations: HQ—hazard quotient, MoE—margin of exposure, RPA—reference point for action, PAHs—polycyclic aromatic hydrocarbons, 5-HMF—5-hydroxymethylfurfural, AFB—aflatoxin B, OTA—ochratoxin A, Pb—lead, As—arsenic, Cd—cadmium, Fe—iron. Created in BioRender. Duda, A. (2026) https://BioRender.com/hebs023.

A comprehensive analysis of the concentrations of the pollutants under study, the risks associated with their presence and the average daily intake through various bee products made it possible to create a risk matrix (Figure 4). This clearly shows in which products cumulative or mixed exposure to harmful components may occur and which contaminants are or are not significant for a given bee product. This unique risk matrix clearly indicates which bee products pose a real risk to consumers’ health, including both healthy individuals and those with allergies, and explains why.

It can be concluded that most of these do not pose a health risk (Figure 4). Nevertheless, there are some exceptions. In honey, the major risk to sensitive consumer populations is posed by antibiotics and pyrrolizidine alkaloids present in some samples in amounts exceeding legal limits. Cases in which the permissible limits of chloramphenicol and pyrrolizidine alkaloids in honey samples were exceeded do not represent a general trend. Rather, they are the result of a failure to observe precautions, a disregard for regulations, or a lack of awareness, mainly in the case of products from outside the EU. An undeniable risk associated with consuming honey is the presence of *Clostridium botulinum* spores. Although the bacterium is unable to produce botulinum toxin in honey due to its high osmotic pressure and intrinsic antimicrobial properties, the spores can germinate in the intestine after ingestion, particularly in infants who lack a fully developed immune system and intestinal microbiota. Subsequently, *C. botulinum* can colonise the intestine and begin to produce the toxin. Of all the bee products, pollen is the most susceptible to contamination by pathogenic microorganisms (mainly *Bacillus cereus* and related species). Unlike bee bread, it does not undergo fermentation in the hive, so its original purity depends primarily on the hygiene of the harvest, the drying process, and storage conditions. Conversely, propolis and royal jelly possess antibacterial, antiviral and antifungal properties; consequently, their contamination stems from unhygienic harvesting, storage or secondary contamination, for example, during packaging.

Consumption of bee pollen may also be connected with health risks due to the presence of certain pesticides (coumaphos, phosmet and carbaryl), mycotoxins (OTA and AFB_1_) and some metals (Pb, As and Fe). Similarly, some metals (Pb, Cd and Fe) and mycotoxins (OTA and AFB_1_) are responsible for the risks associated with perga consumption. The safest bee products are propolis and royal jelly, especially when purchased from known sources. Further research is required to address the knowledge gap surrounding the health risks associated with microplastics, a relatively new threat.

It should be borne in mind that a wide range of factors can influence the levels of hazardous substances in bee products. Naturally, this depends on the type of product, which is linked to its production method (honey versus pollen, bee bread and royal jelly). Furthermore, even within the same type of bee product, significant importance is attached to the botanical origin of the sample, the geographical region, the location of the apiary (i.e., whether it is close to or far from industrial or polluted areas), as well as the method and timing of pesticide application and the doses applied. The physicochemical properties of the active substances in pesticides and antimicrobial preparations are also important.

Another group of factors influencing the levels of hazardous substances in bee products includes environmental conditions, primarily temperature, humidity, water activity, and soil properties. These can vary within the same year, as well as between successive harvesting seasons. The metal content of honey, bee pollen, and other products can vary depending on the geographical region, soil type, and proximity to industrial plants. Even within the same region, these values can differ depending on the plant species from which the bees collect pollen and nectar, the season, or the year of harvest. A similar situation applies to pesticide residues, where agricultural techniques, as well as the frequency and intensity of rainfall and winds, play a significant role in causing pesticides to persist or spread further during spraying. It should be emphasised that the concentrations of hazardous compounds vary considerably between publications, sometimes by several orders of magnitude, due to differences in analytical methods. Researchers use various methods, including atomic absorption spectroscopy (GF AAS, F AAS and ET AAS), inductively coupled plasma (ICP-OES and ICP-MS), neutron activation analysis (NAA), voltammetry, X-ray fluorescence, and cold vapour atomic fluorescence spectroscopy (CVAFS), as well as automated analysers, to detect and quantify metals. Pesticides, on the other hand, are most commonly analysed using liquid chromatography–mass spectrometry (LC-MS/MS), gas chromatography–mass spectrometry (GC-MS/MS), solid-phase extraction (SPE), liquid–liquid extraction (LLE), matrix solid-phase dispersion (MSPD) and QuEChERS (quick, easy, cheap, effective, rugged and safe). Each of these methods has different limits of detection and is suited to specific types of food matrix.

In this review, the average daily intake (AvDI) of honey was set at 16.8 × 10^−6^ kg/kg bw/day in order to calculate the risk of consuming this bee product. The maximum daily intake for bee pollen and perga was set at 2.787 × 10^−4^ and 1.426 × 10^−4^ kg/kg bw/day, respectively. The AvDI for people using propolis and royal jelly prophylactically was set at 1 × 10^−6^ and 0.21 × 10^−6^ kg/kg bw/day, respectively. However, it should be remembered that the average consumption of bee products per capita used in the calculations may vary significantly between populations depending on factors such as age group and dietary habits, or even due to the availability of bee products (and their price). Consequently, the calculated risk associated with honey consumption may, in exceptional cases, be higher. Regardless of this, to minimise the risk of ingesting harmful substances, it is always advisable to check the composition of the honey (monofloral, multifloral, or blend) and its origin (a blend of European and non-EU honeys) and to avoid pollen, propolis or royal jelly products of unclear origin or those sourced from countries without established maximum residue limits (MRLs) or that take a lax approach to regulations concerning the use of pesticides, antibiotics, etc.

In conclusion, this review provides a consolidated presentation of data on the most serious chemical, biological, and physical contaminants detected in honey and other bee products that may have a negative impact on human health. In addition, the sources of these contaminants and the influence of various factors (e.g., geographic region, apiary location, nectar-producing plant species, and measurement method) are discussed. The contaminant levels reported in the literature were also compared with current standards and regulations, and the actual risk to consumer health was determined. The authors suggest that further research is needed to enable the calculation of the hazard index (HI), which is the sum of the HQ values calculated for all harmful factors, such as pesticides or heavy metals. The lack of data to enable the calculation of the HI represents the greatest gap in our current understanding of the safety of honey and other bee products. This is particularly important because the effect of each of these hazardous components when present alongside other constituents in the same product may be completely different to its effect when considered in isolation. Therefore, it is essential to introduce comprehensive testing of bee products rather than merely monitoring whether individual components exceed quality requirements.

From a consumer perspective, a key finding of this review is that the risk of exposure to hazardous components can be minimised by making informed and careful choices when selecting honey and other bee products from a reliable, tested source with a known origin. Ideally, these products should come from Europe, where food quality regulations ensure appropriate standards.

## Data Availability

No new data were created or analyzed in this study. Data sharing is not applicable to this article.

## Appendix A

### Appendix A.1

**Table A1:** Cases of allergic reactions to bee pollen, honey and royal jelly recorded over the last 30 years.

Patient Data (Country)	Cause, Reaction Time	Main Symptoms, Allergy Test Results	References
Bee pollen			
40-year-old man (Republic of Korea)	1 h after consuming a teaspoon of bee pollen	Symptoms: generalised urticaria, facial swelling, shortness of breath, nausea, vomiting, abdominal pain and diarrhoea. Serum-specific IgE to bee pollen and cross-reactivity with weed pollens (chrysanthemum and dandelion) were shown by ELISA.	[460]
30-year-old woman, history of seasonal allergic rhinoconjunctivitis (Canada)	10 min after taking the second dose of bee pollen supplement	Symptoms: anaphylactic reaction; swelling of the eyelids, lips and throat; difficulty swallowing; hives; shortness of breath; a feeling of faintness, dizziness and weakness. PPT positive for bee pollen supplement (20 mm wheal/41 mm erythema) and Timothy grass (20 mm wheal/37 mm erythema); negative for birch, ragweed, and foods.	[580]
56-year-old woman, with allergic rhinitis and atopy (the USA)	Atopic patient experienced a non-life-threatening anaphylactic reaction after ingestion of small quantity of bee pollen for the first time	Symptoms: throat tightness, neck swelling, dyspnoea, generalised urticaria. PPT positive for bee pollen (7 mm diameter wheal/28 mm diameter erythema). Cross-reactivity: bee pollen caused 52% inhibition of IgE binding to short ragweed and 55% to ryegrass.	[461]
15-year-old boy with a history of seasonal allergic rhinitis (Australia)	30 min after vigorous exercise class and 1 h after breakfast containing bee pollen granules, mixed nuts, 5-grain porridge and mixed seeds.	Symptoms: generalized urticaria, facial angioedema and dyspnoea; previously reported abdominal cramps within minutes after bee pollen consumption. PPT positive to the bee pollen consumed (5 × 7 mm) with exercise being a possible co-factor. PPT positive to bee pollen of overseas origin (15 × 10 mm), despite the absence of travel history, indicating the likelihood of a common pollen grain or cross-allergenicity of pollen grains found in both bee pollens; no reaction to exercises alone or to other foods.	[581]
40-year-old man (the USA)	Reaction after taking bee pollen supplement during an intensive outdoor run	Symptoms: congestion, chest tightness, wheeze, shortness of breath, cough, swelling of tongue and hand, dizziness, hives, nausea. Positive PPT (5 mm wheal and 20 mm flare reaction) to a prepared slurry of the bee pollen supplement.	[582]
26-year-old woman with a history of seasonal allergic rhinitis and prior exercise-induced asthma (the USA)	Within 4 h after bee pollen ingestion	Symptoms: abdominal pain, nausea, dyspnoea, shortness breath, swelling of upper lip and tongue, ‘itching all over the body’ without a rash; development of allergic asthma with subsequent anaphylaxis after bee pollen ingestion; BP 114/78 mmHg, HR 96 bpm, respirations 20/min. Elevated levels of total serum IgE and specific IgE to ragweed, cocklebur, mugwort, sheep sorrel and rough pigweed.	[583]
27-year-old-man Patient with seasonal allergic rhinitis and oral allergy syndrome caused by watermelons, Korean melons, peaches, and plums (Republic of Korea)	Anaphylaxis during consuming a bowl containing half a tablespoon of bee pollen and honey mixed with rice	Symptoms: swelling around the eyes and lips, sensation of swelling in the throat, chest tightness, wheezing, abdominal pain, vomiting, dizziness, BP 92/53 mmHg, HR 80 bpm, respirations 22/min, SpO_2_ 92%. Positive PPT to bee pollen (15 × 7 mm), ragweed 6 × 6 mm), dandelion (9 × 8 mm), mugwort (12 × 10) and various tree pollens; specific IgE to honey bee venom was negative.	[584]
35-year-old man (Malaysia)	15 min after taking bee pollen granules	Symptoms: itchiness, redness and swelling of lips and gums, nausea, dizziness, cough, wheezing, chest tightness; BP 86/56 mmHg. RAT: specific IgE detected for chrysanthemum and dandelion pollens (both class 4) The case was diagnosed as immediate hypersensitivity to bee pollen supplement.	[585]
32-year-old atopic patient and a history of allergic rhinitis (Spain)	Severe anaphylactic reaction a few minutes after ingestion of a preparation containing a pollen compound	Symptoms: generalised itching, widespread erythema, facial swelling, cough, hoarseness, dysphonia. Skin tests revealed an allergy exclusively to *Artemisia vulgaris*, *Taraxacum officinalis* and *Salix alba.* Specific IgE level were classified to the class 3, 2 and 2, respectively. Microscopic analysis of the pollen mixture samples showed that it consisted of 93% pollen, including all three allergenics	[586]
4-year-old boy, with seasonal allergic rhinitis (spring and autumn, 2 months before incident) (Spain)	Immediately after ingesting a small spoonful of bee pollen for the first time;	Symptoms: intense mouth, tongue and pharynx itching, uvula oedema, difficulty swallowing, isolated hives on the thorax. Positive PPT to bee pollen (10 mg/mL) and allergen extracts from locally occurring pollens (*Lolium perenne, Cynodon dactylon*, *Olea europaea*, *Platanus acerifolia*, *Plantago lanceolata*, *Cupressus arizonica, Artemisia vulgaris* and *Taraxacum officinalis*). Serum-specific IgE to bee pollen was 6 kU/L.	[450]
Honey			
65-year-old man with only allergic rhinoconjunctivitis and type I sensitization to various pollen, treated with mitomycin C for urothelial carcinoma (Germany)	Aprox. 2–3 h during first episode and after 90 min during second episode after eating blossom honey, wheat bread, and strawberry jam for breakfast	Experience of grade III anaphylaxis. Symptoms: during first episode: erythema, collapse, dyspnoea, and defecation. During second episode: generalised flushing, hypotension, dyspnoea, tachypnoea, defecation, and tongue swelling. Both episodes required intensive care unit (ICU) stabilisation. PPT positive to blossom honey (10 mm wheal, 22 mm erythema). Specific IgE to honey bee venom (1.56 kU/L)—classified as class 2. Diagnosis of recurrent grade III anaphylaxis triggered by severe type I hypersensitivity to honey.	[587]
28-year-old woman with allergic rhinitis (the USA)	Within 30 min of eating a honey butter chicken biscuit and hash browns. The biscuit contained honey, egg, wheat, onion, and garlic. The use of new toothpaste containing bee propolis had recently been initiated.	Symptoms: hives, vomiting, cramps, diarrhoea. Positive serum-specific IgE tests (kU/L) to honey (31.2), egg white (2.15), garlic (0.22), and negative to potato, onion, parsley, oregano, and wheat.	[588]
19-year-old woman with summer rhinoconjunctivitis to *Compositae* pollen (Italy)	Anaphylactic shock 10 min after eating bread and honey (obtained from bees who had foraged on Compositae flowers)	Symptoms: lip and tongue angioedema, runny nose, cough, dyspnoea, dizziness, followed by collapse; PPT positive to mugwort, ragweed, dandelion, and goldenrod pollen, and to sunflower, lime tree, and Eucalyptus honey.	[589,590]
5-year-old boy, was sensitized to Compositae pollen (ragweed and mugwort) (Italy)	Anaphylaxis 30 min after the ingestion of a meal containing salmon and artisanal honey	Symptoms: generalised urticaria, respiratory distress, SpO_2_ 93%. PPT positive to artisanal honey and negative to various commercial honey. PPT and specific IgE positive (kUA/L) for grass (16.5), ragweed (7.93) and mugwort (35.3) pollens. serum IgE antibodies to bee venom yielded a weak positive result (0.65 kUA/L). The reaction tryptase level was 6.63 μg/L indicating a diagnosis of anaphylaxis.	[591]
40-year-old female, no personal history of atopy and Hymenoptera sting hypersensitivity; suspected allergy to honey (Portugal)	Anaphylaxis 30 min after carrying out the Prick-to-prick tests with several honeys	Symptoms: generalized urticaria, swollen of lips-tongue-uvula, hypotension. PPT positive for 9 various kinds of honey, whereas no allergic symptoms were noted to pollens, bee venom, royal jelly and propolis. Serum total IgE (98 kU/L, specific IgE to honey (1.1 kU/L). Earlier she had 2 episodes: at the age of 36 generalized urticaria 20 min after ingestion of foods with honey (honey cake and a banana with honey) and at the age of 37, she got swollen lips 5 min after accidental contact with a teaspoon with honey traces on it, followed within 10 min by urticaria and angioedema.	[471]
35-year-old man (Iraq)	15 min after consumption of local honey product	Symptoms: abdominal pain, skin irritation, stomach cramps, discomfort, or more severe such as vomiting, watery diarrhoea, dizziness, and fainting. Patient visited the hospital about 6 times within the year. PPT positive to honey.	[592]
27-year-old man (the USA)	Within 30 s of consuming various honey infusions (degustation)	Symptoms: generalized urticaria, skin redness, flushing, presyncope, lip angioedema, enlarged uvula, tachycardia (HR 135 bpm), BP 152/46 mmHg. Positive PPT for wildflower and orange honey, negative for other tested honeys. Negative specific IgE against honey bee.	[593]
14-month-old boy (Turkey)	Anaphylaxis 5 min after ingestion of approximately 5 teaspoons of honey at 14 months of age. The child had previously been given one teaspoon of honey several times until he was six months old.	Symptoms: swollen lips, then, within 10 min, urticaria, angio-oedema, cough and wheezing. PPT (five weeks after the anaphylactic reaction) was positive for the honey that had been consumed.	[594]
20-month-old boy (Republic of Korea)	30 min after eating rice cake containing honey	Symptoms: skin rash and itching starting from his torso, eye swelling; 2 weeks after food challenge tests with 0.5 mL of acacia honey was performed resulting in anaphylaxis, lip oedema and dyspnoea, chest retraction, BP 100/60 mmHg, HR 92 bpm, SpO_2_ 97%. Total serum IgE was elevated at 144.6 IU/mL. A multiple allergen simultaneous test (MAST) for ~60 common inhalant and food allergens (including bee venom, mugwort, and birch) showed no sensitization.	[595]
45-year-old woman with a Mueller grade-4 systemic reaction after a wasp sting, no comorbidities (Turkey)	multiple anaphylaxis episodes, within 1 min of digestion honey, thyme or black pepper (at different times)	Symptoms: widespread itching, urticaria, laryngeal oedema, difficulty in breathing, loss of consciousness, urinary incontinence. Among *Hymenoptera* skin prick tests, *Apis mellifera* showed a 3 × 3 mm wheal and 5 × 6 mm erythema; reactions to thyme, black pepper and wasp were IgE-mediated, but the etiology of her honey allergy was not possible to determine. Increased serum-specific IgE for *A. mellifera* (10.4 kU/L), Vespula (4.58 kU/L), and Ves-v5 (0.42 kU/L). Other specific IgE were below normal value, i.e., <0.35 kU/L (Api-m1 = 0.17, Ves-v1 = 0.3).	[596]
Royal jelly (RJ)			
7-year-old girl (Italy)	10 min after ingestion royal jelly prescribed by physician	Symptoms: lip and tongue oedema, itch. PPT positive to royal jelly (6 mm wheal), other test indicate hypersensitivity to almond and Compositae pollens. Elevated levels (range between 0.12 and 0.19 kUA/L) of specific IgE against honey bee, yellow jacket, white-faced hornet, and bumblebee, but still lower than IgE level for various allergenic plants.	[597]
26-year-old woman with 20-year history of bronchial asthma (Japan)	Anaphylaxis 30 min after taking RJ tablet.	Symptoms: dyspnoea, severe oedema of the face and eyelids, congestion of the conjunctivae and face erythema. PPT positive to RJ (mm wheal/mm erythema): 7/20 × 15 (dilution 1/10), 5/30 × 20 (1/100), and RJ tablet (5/25, dilution 1/10). Prior to the onset of the symptoms, drinks containing RJ had been consumed, but never RJ tablets. RAT: Serum IgE level was elevated (239 IU/mL). IgE antibodies to honeybees were below 0.34 UA/mL.	[598]
26-year-old woman with history of bronchial asthma, allergic rhinitis, allergic conjunctivitis, atopic dermatitis and food allergy (Japan)	Anaphylaxis 15 min after drinking a beverage of crude royal jelly including honey.	Symptoms: wheezing, hypoxemia, generalized erythema, eyelid itching and swelling. PPT performed with the beverage of crude royal jelly including honey (positive, 5 × 6 mm wheal/30 × 20 mm erythema) and with two other beverages including honey (both negative).	[599]
33-year-old man (Japan)	Anaphylaxis after ingestion of RJ and several other medicinal products.	Symptoms: facial followed by generalised itching, erythema, then vertigo, numbness in his fingers, wheals, dyspnoea, wheezing and impaired consciousness. PPT positive to RJ (20 × 20 mm wheal/40 × 40 mm erythema).	[600]
53-year-old woman (Japan)	Haemorrhagic colitis associated with royal jelly intake (for 25 days).	Symptoms: abdominal pain, bloody diarrhoea, BP 128/60 mmHg, HR 70 bpm. Colonoscopy and histopathology revealed mucosal haemorrhage, oedema, and infiltration of inflammatory cells. Total and specific IgE not elevated. Positive drug-induced lymphocyte stimulation test for RJ (stimulation index 147%).	[601]
7 persons (Australia)	Asthma and/or anaphylaxis after RJ ingestion	Symptoms: acute and severe asthma attacks, bronchospasm, wheezing, and shortness of breath, followed by life-threatening anaphylaxis and anaphylactic shock as part of an IgE-mediated hypersensitivity reaction. IgE antibodies to RJ proteins were detected. More than half subjects with allergies to inhalant and/or food allergens revealed IgE antibody reactivity to RJ proteins.	[602]
56-year-old woman (China)	2 anaphylaxis episodes within 1 h after RJ ingestion (within one month).	Symptoms: urticaria, itching, laryngeal oedema, chest tightness, hypotension, collapse. PPT positive to RJ (4 × 4 mm wheal, 37 × 16 mm erythema) and honeycomb (3 × 3 mm and 36 × 20 mm, respectively). The 62 kDa protein belonging to MRJP3 was identified as the allergen responsible for anaphylaxis and cross-reactivity with honeycomb. Serum IgE specific for 60 kDa protein was present in royal jelly and Honeycomb (probably MRJP 3) and for 70 kDa protein shown in honeycomb (probably MRJP 5).	[603]

ECG—electrocardiography, PPT—prick-to-prick test (PPT), HR—heart rate, BP—blood pressure, MRJP—Major Royal Jelly Protein, RJ—royal jelly, RAT—radioallergosorbent test, RR—respiratory rate; bpm—beats per minute, SpO_2_—saturation of peripheral oxygen.

### Appendix A.2

#### Appendix A.2.1. Methodology for Calculating Human Risk

The hazard quotient (HQ) for each toxic compound described in the main text of the manuscript was calculated as follows:

$$HQ=\frac{EDI}{RfD}$$

where

EDI is estimated daily intake (mg/kg bw/day);

RfD is oral reference dose (mg/kg bw/day).

HQ < 1 indicates an acceptable exposure level with minimal non-carcinogenic risk, while an HQ > 1 suggests potential adverse health concerns [604].

EDI was calculated by multiplying the maximum concentration (C_max_) of the compound in a given product type (H—honey, BP—bee pollen, P—perga, PR—propolis, RJ—royal jelly, BW—bee wax) by the average daily intake (AvDI) of that product (kg/kg bw/day).

#### Appendix A.2.2. Assumption for Calculations of AvDI Values Used in the Manuscript

- Honey

Based on FAO data, the calculated average honey consumption in 2019 per capita ranged from 0.01 kg/year (India) to 1.17 kg/year (Turkey), with the global average annual honey consumption standing at 0.43 kg per capita [95]. This means that daily consumption per capita is 11.78 × 10^−4^ kg of honey. These figures, of course, vary significantly by region and depend, among other factors, on dietary habits, cultural customs, as well as the availability of honey in a given country and its price (the need to import honey drives up its price).

The AvDI for honey was set at 16.8 × 10^−6^ kg/kg bw/day (assuming a person weighs 70 kg).

- Bee pollen

There are no data on the annual human consumption of bee pollen. Therefore, the calculations take into account the fact that apitherapy specialists typically recommend treatment regimens involving bee pollen lasting 1 to 3 months, repeated twice a year (in autumn and spring). The standard preventive dose for adults is 20–40 g (about 2–4 teaspoons) per day. Converting these recommendations to annual consumption, we obtain the following:

Minimum treatment (2 × 1 month per year, 2 teaspoons each) corresponds to approximately 1.2 kg of pollen per year per capita.

Intensive treatment (2 × 3 months per year, 4 teaspoons each) corresponds to approximately 7.1 kg of pollen per year per capita.

For further calculations in the manuscript, the maximum AvDI value for bee pollen, which was set at 2.787 × 10^−4^ kg/kg bw/day, was used.

- Perga

Similarly, there are no data on the annual human consumption of perga. Perga is best taken in two or three treatment courses per year, mainly in autumn and spring. Each course typically lasts 1–2 months, followed by a break. The recommended daily preventive dose of perga is 10–20 g (about 1–2 teaspoons). Assuming the shortest treatment course lasts 60 days per year (two 1-month courses) and the lowest dose is taken (10 g), the average annual consumption would be 0.6 kg per person. Maximum consumption, assuming three 2-month treatment courses at a dose of 20 g per day, results in an average annual consumption of 3.65 kg per person.

For further calculations in the manuscript, the maximum AvDI for perga set at 1.426 × 10^−4^ kg/kg bw/day, was used.

- Propolis

Propolis is widely used in cosmetics and skincare products, but in apitherapy, it is also ingested for preventive purposes. Assuming a safe daily dose of pure propolis of 70 mg per day throughout the year, the annual intake would be 25,500 mg. It is worth noting that clinical trials use higher doses, even up to 500 mg per day for targeted treatments.

The AvDI for a person using propolis prophylactically is 1 × 10^−6^ kg/kg bw/day.

- Royal jelly

Royal jelly is not usually considered a standard food but a specialised dietary supplement or pharmaceutical raw material. It is usually taken as part of 1–2 health regimens lasting a total of 4 to 8 weeks (in spring and autumn), with a recommended daily dose of 50–100 mg of pure, fresh royal jelly for adults. In some countries, such as France, higher doses of 250–500 mg are recommended. Taking a total of 8 weeks of standard treatment per year at a daily dose of 100 mg results in an annual consumption of approximately 5.6 g of pure royal jelly per person.

The AvDI for a person consuming royal jelly prophylactically is 0.21 × 10^−6^ kg/kg bw/day.

#### Appendix A.2.3. Reference Doses for Hazardous Chemicals Identified in Honey and Other Bee Products

The Reference Dose (RfD) is an estimate of the maximum amount of a chemical compound that humans can ingest daily throughout their lifetime without experiencing significant health issues. Hence, RfD is not assigned to a specific product category but is linked only to the route of exposure (digestion). RfD values used in the manuscript were set basing on data from official web pages of U.S. EPA [43], EPA Human Health Benchmarks for Pesticides, FAO guidelines, European Commission Pesticides Database (https://ec.europa.eu/food/plant/pesticides/eu-pesticides-database/start/screen/active-substances (accessed on 26 August 2026)) and publications [19,21,25,96,111,117,119,225,234,240,538,604,605,606,607,608,609,610]. If the RfD has not been specified in current legislation, historical data, the Acceptable Operator Exposure Level (AOEL) or the Minimal Risk Level were used for the calculations.

It should be noted that different sources may provide different RfD values. Therefore, if different values were found, the lowest of these was used for calculations. These differences stem from the fact that they are based on studies demonstrating harmful effects on various organs, tissues, or systems. For example, a different dose of a substance can have toxic effects on the nervous system, while another dose can affect the endocrine system, digestive tract, reproductive system, or circulatory system.

Table A2 summarises the most significant metals and pesticides detected in honey and bee products [27,117,131,148,149,226,229,237,611,612,613,614,615,616,617,618,619,620,621]. The table shows the RfD values and the Maximum Residue Limits (MRLs) for pesticides and the Maximum Levels (ML) for metals as specified by statutory regulations. The concentration of each substance is given as the highest value reported for a given product type, which was then used to calculate the hazard quotient (HQ).

**Table A2:** The maximum reported concentrations (C_max_) of metals and pesticide residues in various types of bee products, together with their respective oral reference doses (RfDs), maximum residue limits (MRLs) for pesticides and maximum levels (MLs) for metals and metalloids, as well as the calculated hazard quotients (HQs). HQ values greater than 1.0 are highlighted in bold.

Chemical	RfD (mg/kg bw/day)	MRL or ML (mg/kg)	C_max_	HQ
*Metals and metalloids (concentration in mg/kg)*				
Cadmium (Cd)	0.003 (food) 0.0005 (water)	SUP: 1.0	H: 3.81 BP: 2.97 P: 67.06 PR: 76.69 BW: 0.284	0.02134 0.36603 **3.18759** 0.02556 -
Lead (Pb)	0.00035–0.004 (depending on region)	H: 0.1 SUP: 3.0	H: 3.41 BP: 4.23 P: 4.05 PR: 160.1 BW: 5.43 RJ: 2.80	0.16368 **3.36829** **1.65009** 0.45743 - 0.00168
Copper (Cu)	0.04	–	H: 58.1 BP: 26.3 P: 21.43 PR: 33.4 BW: 40.9 RJ: 12.78	0.02440 0.18325 0.07640 0.00084 - 0.00007
Chromium (Cr(VI))	0.003–0.005 (depending on region)	–	H: 18.8 BP: 6.94 P: 1.74 PR: 48.9 BW: 56.28 RJ: 2.97	0.10528 0.64473 0.08271 0.01630 - 0.00021
Nickel (Ni)	0.02	–	H: 5.59 BP: 8.41 P: 27.0 PR: 75.9 BW: 17.8 RJ: 1.70	0.00470 0.11719 0.19251 0.00380 - 0.00002
Arsenic (As)	0.0003	–	H: 1.66 BP: 13.60 P: 0.89 PR: 8.47 BW: 0.031 RJ: 0.074	0.09296 **12.63440** 0.42305 0.02823 - 0.00005
Zinc (Zn)	0.3	–	H: 40.1 BP: 159.3 P: 130.2 PR: 2790.0 BW: 729.0 RJ: 66.44	0.00225 0.14799 0.06189 0.00930 - 0.00005
Mercury (Hg)	0.003	SUP: 0.1	H: 1.25 BP: 0.006 P: 0.008 PR: 0.018 BW: <LOQ RJ: 0.036	0.00700 0.00056 0.00038 0.00001 - 0.00000
Cobalt (Co)	0.003	–	H: 0.166 BP: 0.06 PR: 0.08 BW: 0.16	0.00093 0.00557 0.00003 -
Iron (Fe)	0.007	–	H: 59.6 BP: 811 P: 565.0 PR: 2125 BW: 334.0 RJ: 23.37	0.14304 **32.28939** **11.50986** 0.30360 - 0.00070
*Pesticides (concentration in μg/kg)*				
Acetamiprid	0.005	1.0	H: 31 BP: 134 P: 17 PR: < LOQ RJ: < LOQ	0.00010 0.00747 0.00048 - -
Acrinathrin *	0.01	0.05	H: 2400 BP: 9.9 BW: 4447	0.00403 0.00028 -
Amitraz *	0.01	0.2	H: 555 BP: 1117 BW: 46,060	0.00093 0.03113 -
Atrazine *	0.1	0.05	H: 81 BP: 49 BW: 31	0.00001 0.00014 -
Azoxystrobin	0.18	0.05	H: 14 BP: 2900 P: 71.6 PR: < LOQ RJ: < LOQ BW: 278	0.00000 0.00449 0.00006 - - -
Bifenthrin	0.03	0.05	H: 241.7 BP: 180 P: 1462 BW: 56.1	0.00014 0.00167 0.00695 -
Biphenyl *	0.5	0.01	BP: 11 PR: 42 RJ: < LOQ	0.00001 0.00000 -
Boscalid	0.22	0.15/0.05	H: 10.0 BP: 1496 P: 50.4 PR: < LOQ RJ: < LOQ BW: 820	0.00000 0.00190 0.00003 - - -
Carbaryl *	0.01	0.05	H: 42 BP: 94,000 BW: 5.5	0.00007 **2.61978** -
Carbendazim *	0.02	1.0	H: 1273 BP: 4516 P: 99 BW: 133	0.00107 0.06293 0.00071 -
Chlorfenvinphos *	0–0.0005	0.01	H: 16 BP: 10.7 BW: 7620	0.00054 0.00596 -
Chlorpyrifos *	0–0.01	0.01	H: 22.3 BP: 956 P: 22.6 PR: 110 RJ: < LOQ BW: 890	0.00004 0.02664 0.00032 0.00001 - -
Chlorothalonil *	0.05	0.05	H: 10 BP: 98,900 BW: 53,700	0.00000 0.55127 -
Clothianidin *	0.1	0.05	H: 2.1 BP: 236.4	0.00000 0.00066
Coumaphos *	0.0007–0.0025	0.1	H: 2020 BP: 5828 BW: 94,900	0.04848 **2.32038** -
Cypermethrin	0.005	0.05	H: 92 BP: 1900 PR: < LOQ RJ < LOQ BW: 131	0.00031 0.10591
Cyprodinil	0.03	0.05	H: 31.0 BP: 1283 P: 1965 PR: < LOQ RJ < LOQ BW: 106	0.00002 0.01192 0.00934 - - -
Dichlorvos *	0.002	–	H: 8	0.00007
Dimethoate *	0.0002	0.01	H: 24 BP: 210 P: 1.2 PR: 31 BW: 133	0.00202 0.29264 0.00086 0.00016 -
Dimethomorph *	0.6	0.05	BP: 2678 BW: 133	0.00124 -
Endrin *	0.0003	0.01	H: 7 BP: 218.57	0.00039 0.20305
Fenhexamid	0.2	0.05	H: 274 BP: 7178 P: 11.9 BW: 9	0.00002 0.01000 0.00001 -
Fenpropathrin *	0.03	–	H: 1323 BP: 170 BW: 200	0.00074 0.00158 -
Fenvalerate *	0.011–0.025	0.05	H: 4655 BP: 58 P: 3726 PR: < LOQ RJ: < LOQ	0.00711 0.00147 0.04830 - -
Fipronil *	0.009	0.005	H: 2099 BP: 39 BW: 35.9	0.00392 0.00121 -
Fluopyram	0.5	0.05	H: 4050 BP: 134.3 P: 28	0.00014 0.00007 0.00001
Glyphosate	1.5	0.05	H: 5.5 P: 0.7 BW: 0.32	0.00000 0.00000 -
Hexaconazole *	0.02	–	BP: 369 P: 10.30	0.00514 0.00007
Imidacloprid *	0.08	0.05	H: 784.7 BP: 912 BW: 13.6	0.00016 0.00318 -
Malathion	0.3	0.05	H: 243 BP: 61 BW: 6000	0.00001 0.00006 -
Myclobutanil *	0.31	0.05	H: 334 BP: 4190 P: 12.4 BW: 5	0.00002 0.00377 0.00001 -
Pendimethalin	0.3	0.05	H: 1810 BP: 1730 PR: < LOQ RJ: < LOQ BW: 84	0.00010 0.00161 - - -
Phosmet *	0.001	0.05	H:42.1 BP: 16,556 BW: 209	0.00071 **4.61416** -
Propamocarb	1.0	0.05	BP: 180 PR: < LOQ RJ: < LOQ	0.00005 - -
Tau-fluvalinate Fluvalinate	0.05	0.05	H: 750 BP: 16,260 BW: 204,000	0.00025 0.09063 -
Tebuconazole	0.03	0.05	H: 4530 BP: 484.5 P: 59.7 PR: < LOQ RJ: < LOQ	0.00254 0.00450 0.00028 - -
Thiacloprid *	0.02	0.05	H: 337 BP: 1002 P: 36.6 BW: 7.8	0.00028 0.01396 0.00026 -
Thiamethoxam *	0.5	0.05	H: 15.6 BP: 53	0.00000 0.00003
Triadimefon *	0.08	0.05	BP: 66.0 P: 24.4 BW: 2	0.00023 0.00004 -

* Chemical compounds no longer approved for use under EU regulations but that remains present in the environment; H—honey, BP—bee pollen, P—perga, PR—propolis, RJ—royal jelly, BW—bee wax, LOQ—the limit of quantification that was given by the authors of the only publication that analysed a particular compound, SUP—maximum level permitted for food supplements. No HQ values were calculated for beeswax, as its consumption is negligible and any pesticide or heavy metal residues it contains are unlikely to pose a significant risk.

As shown in Table A2, the majority of the calculated HQ values are less than 1, which means that the metals present in honey and bee products do not pose a risk to consumers. This is consistent with the findings of other authors. It should be borne in mind, however, that the analysed honeys are often mixtures of different honeys, and therefore, the reported values are averages. It is consequently possible that, for example, during transport in unsuitable containers, a batch of a given bee product could become significantly contaminated, creating a more realistic risk to the consumer. The situation is similar for pesticide residues; studies show considerable variability in the concentrations detected, depending on the time of year, the location of the apiary and hives, or the specific season. It cannot be ruled out that honey or other bee products harvested too soon after spraying may contain significantly higher concentrations of pesticides. This risk is further compounded because, in addition to honey, pollen, propolis, etc., consumers also eat fruit and vegetables, which likewise introduce pesticide residues into the human body. This means that although the HQ for individual products does not indicate a risk, the cumulative HI may already reach warning or dangerous levels. The HQ values presented in this study may underestimate the risk for individuals with high intake levels who consume bee products from contaminated regions, as well as for particularly vulnerable groups (children and pregnant women). In the near future, it will be necessary to address these gaps, including by introducing regulations that not only require the monitoring of pesticide residue contamination but also prescribe a measurement method to facilitate the comparison of results.
